# Sequencing and Characterisation of Rearrangements in Three *S. pastorianus* Strains Reveals the Presence of Chimeric Genes and Gives Evidence of Breakpoint Reuse

**DOI:** 10.1371/journal.pone.0092203

**Published:** 2014-03-18

**Authors:** Sarah K. Hewitt, Ian J. Donaldson, Simon C. Lovell, Daniela Delneri

**Affiliations:** Faculty of Life Sciences, University of Manchester, Manchester, United Kingdom; The University of Nottingham, United Kingdom

## Abstract

Gross chromosomal rearrangements have the potential to be evolutionarily advantageous to an adapting organism. The generation of a hybrid species increases opportunity for recombination by bringing together two homologous genomes. We sought to define the location of genomic rearrangements in three strains of *Saccharomyces pastorianus,* a natural lager-brewing yeast hybrid of *Saccharomyces cerevisiae* and *Saccharomyces eubayanus*, using whole genome shotgun sequencing. Each strain of *S. pastorianus* has lost species-specific portions of its genome and has undergone extensive recombination, producing chimeric chromosomes. We predicted 30 breakpoints that we confirmed at the single nucleotide level by designing species-specific primers that flank each breakpoint, and then sequencing the PCR product. These rearrangements are the result of recombination between areas of homology between the two subgenomes, rather than repetitive elements such as transposons or tRNAs. Interestingly, 28/30 *S. cerevisiae*- *S. eubayanus* recombination breakpoints are located within genic regions, generating chimeric genes. Furthermore we show evidence for the reuse of two breakpoints, located in *HSP82* and *KEM1*, in strains of proposed independent origin.

## Introduction

Hybridisation in *Saccharomycetous* yeast occurs readily in natural and industrial environments [1], [2], [3], [4], [5], [6], [7], and may be a swift mechanism for evolutionary innovation. Investigating the genomics of successful natural hybrid species can provide valuable evolutionary insight into how the union of diverged genetic material can sculpt a genome more suited to its new environmental niche. These adaptations may include chromosomal rearrangements such as duplication, translocation, inversion and selective loss of genes or even whole chromosomes. The lager yeast *Saccharomyces pastorianus*, previously classified as *Saccharomyces carlsbergensis*, is a natural hybrid between *Saccharomyces cerevisiae* and a *Saccharomyces uvarum*-like species [4], [8], [9], [10]. The *S. uvarum-*like species has most recently been identified as the Argentinean-isolate *Saccharomyces eubayanus*, which shows 99.5% identity to the non-*S. cerevisiae* portion of *S. pastorianus* [11]. *S. pastorianus* is thought to have arisen by spontaneous hybridisation in brewery conditions, maintained by human selection for colder brewing temperatures, a preference that is conferred by its *S. uvarum-*like parent [12].

So far, there are only two whole genome lager yeast sequences available, Weihenstephan 34/70 [13] and CCY48–91, which has been recently deposited in Genbank [ID:ALJS00000000.1]. Much of our knowledge of the genome composition of these natural hybrids derives from previous array-based comparative genomic hybridisation studies (array-CGH) performed on 17 strains of *S. pastorianus* [10]. This particular work identified two groups of lager yeasts: Group 1 strains contain roughly one haploid *S. cerevisiae* and one haploid *S. eubayanus* genome with significant loss of *S. cerevisiae* genes, whereas Group 2 strains contain one haploid *S. eubayanus* genome and a diploid *S. cerevisiae* genome. The differences between these two groups suggest that they may have had independent evolutionary origins, a theory given weight by both the aforementioned array-CGH analysis [10] and the differing distribution of transposons between the two groups [14]. Additionally, strains within each group are highly variable in their patterns of chromosomal loss, aneuploidy and gross chromosomal rearrangements, probably reflecting either evolutionary pressure from diverse brewery conditions or random genetic drift [9], [10].

Lager yeast chromosomes have been shown to have undergone recombination, generating chimeric chromosomes composed of genetic material from both parental species [10], [13], [15], [16]. Typically, recombination between chromosomes within a non-hybrid yeast species is thought to be mediated primarily by transposons (Ty elements) [15], [17], [18], [19], tRNAs [17], [18], duplicated genes [18] or, as more recently proposed, origins of replication [20]. However, breakpoint formation in *S. pastorianus* is thought to be either Ty-mediated [10], [15] or the result of recombination between homologous regions [13]. Studies have also demonstrated the role of high stress brewery conditions in promoting genomic rearrangements, such as localised areas of gene amplification and recombination [21]. Significantly, chromosomal rearrangements have been shown to confer adaptive traits in both wild and industrial yeasts including highly sulphite-resistant wine yeast [22], *flor* wine yeast [23] and wild copper-tolerant yeast [24]. Furthermore, rearrangements have been shown to contribute to speciation between species of yeast [25].

We sequenced three *S. pastorianus* strains to both characterise genomic breakpoints and shed further light on their formation and retention. We chose strains that have been used in a previous microarray study to provide a source of validation for our sequencing [10]. These strains have also been pre-classified into one of the two aforementioned groupings of *S. pastorianus*: two of the chosen strains of *S. pastorianus* are of Group 1 (DBVPG 6033 and DBVPG 6261) and one is of Group 2 (DBVPG 6257) [10]. These particular strains have the greatest level of differential gene loss and therefore the least amount of redundancy. The latter group is thought to have an independent evolutionary origin from the former group, allowing us to investigate similarities between non-related strains.

We have confirmed the location of many *S. cerevisiae*- *S. eubayanus* breakpoints at the single nucleotide level and identified both nearby repetitive elements and regions of homology. Significantly, we found that the majority of genomic breakpoints occurred within protein coding regions, generating chimeric genes. Furthermore, the presence of identical breakpoints in *KEM1* and *HSP82* is evidence of breakpoint reuse between strains of proposed independent origin.

## Results and Discussion

### Genome Assembly and Analysis

**Figure 1:**
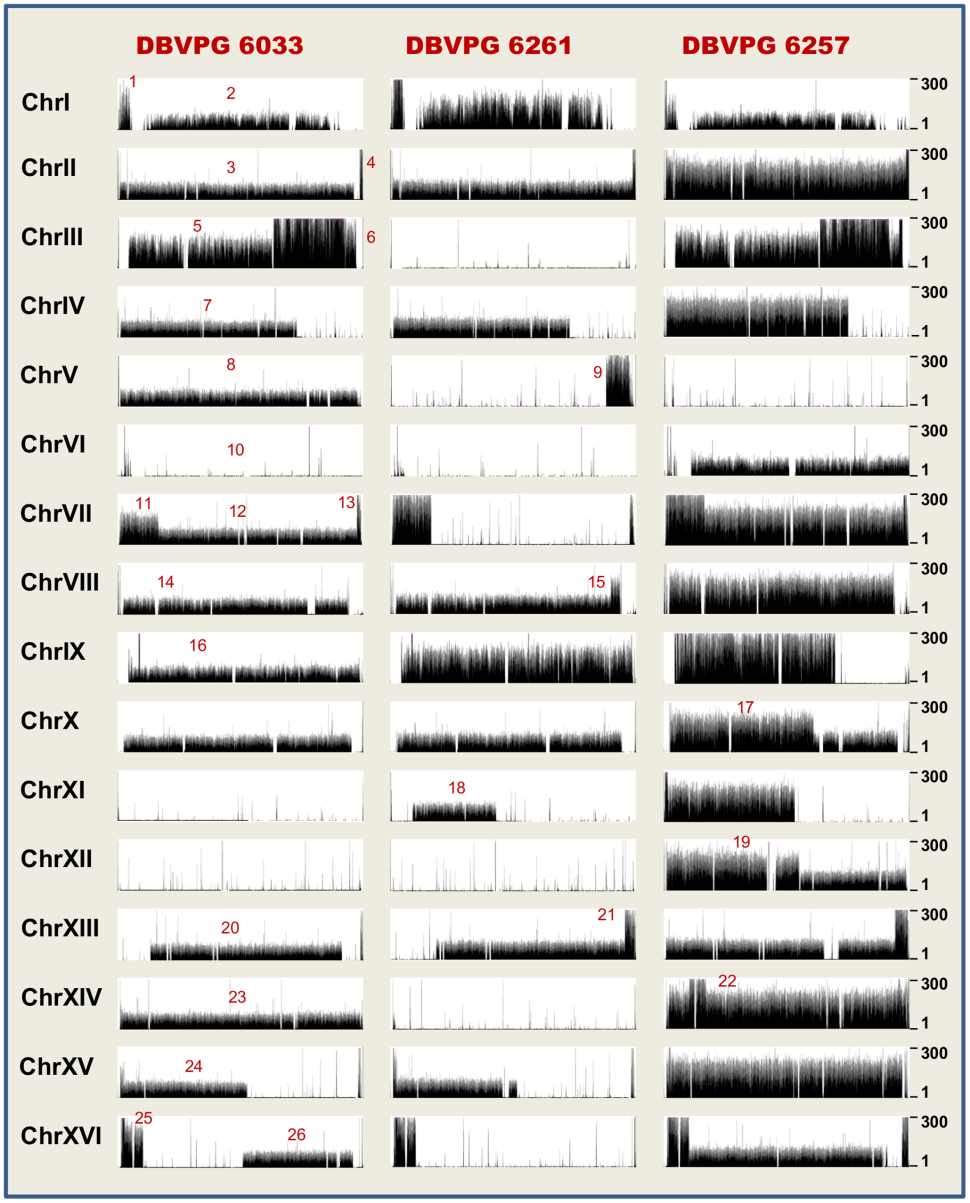
Representation of *S. pastorianus* reads mapped to *S. cerevisiae* chromosomes in the UCSC Genome Browser. Full set of *S. cerevisiae* chromosomes is displayed for each strain using the UCSC Genome Browser. Depth of track corresponds to read number. Track depth window is set to a read depth of 300 to accommodate three copies of a chromosome, since *S. cerevisiae* chromosome copy number in these strains generally varies between zero and three. Regions used in copy number analysis are labelled 1–26 in red.

The genomic DNA of three strains of *S. pastorianus*, DBVPG 6033 (*Saccharomyces carlsbergensis* type strain), DBVPG 6261 (*Saccharomyces monacensis* type strain) and DBVPG 6257 were sequenced using the SOLiD 4 Next Generation Sequencing platform and mapped to *S. cerevisiae* (sacCer2) and *S. uvarum* (sacBay MIT), which are representative of the *S. pastorianus* subgenomes. We used sacBay MIT as the reference genome for *S. eubayanus* due to its fully available sequence, which is purportedly 7% diverged from *S. eubayanus* [11]. Visualisation of the *S. cerevisiae* chromosomes in the UCSC Genome Browser (http://genome.ucsc.edu/) is reported in Figure 1. *S. eubayanus* reads mapped to contigs were viewed in the Integrative Genomics Viewer (http://www.broadinstitute.org/igv). SOLiD sequencing allowed us to ascertain the approximate chromosomal copy number in each strain using a hierarchical cluster analysis of relative median read depth across multiple regions (Tables S3–S4; Figure S3). In total, DBVPG 6033, 6261 and 6257 are estimated to have 31, 31 and 48 chromosomes respectively (Table 1). These chromosomes map to *S. cerevisiae*, *S. eubayanus* or a combination of both *S. cerevisiae* and *S. eubayanus* sequence (chimeric chromosomes).

**Table 1:** Estimation of chromosome copy number in *S. pastorianus.*

DBVPG strain	6033	6261	6257	6033	6261	6257	DBVPG strain	6033	6261	6257
Sc chromosome	*S. cerevisiae* copies			Chimeric copies			Se chromosome^a^	*S. eubayanus* copies		
I	1	2	1	0	0	0	I	0	0	2
II	0	1	2	1	0	0	II–IV	1	1	1
III	2	0	2	1	0	1	III	0	1	0
IV	0	0	0	1	1	2	IV-II	1	1	1
V	1	0	0	0	2	0	V	1	0	3
VI	0	0	1	0	0	0	X-VI	1	1	1
VII	1	0	2	1	3	1	VII	0	0	0
VIII	0	1	2	1	1	0	VIII–XV	1	1	1
IX	1	2	0	0	0	3	IX	1	0	0
X	1	1	1	0	0	1	VI–X	1	1	1
XI	0	0	0	0	1	2	XI	2	1	2
XII	0	0	1	0	0	0	XII	2	2	2
XIII	0	0	0	1	2	3	XIII	1	0	0
XIV	1	0	2	0	0	1	XIV	1	1	0
XV	0	0	2	1	1	0	XV-VIII	1	0	1
XVI	0	0	0	2	3	3	XVI	0	0	0
Total	8	7	16	9	14	17	Total	14	10	15
							**Total chromosomes**	**31**	**31**	**48**

An estimation of chromosome number based the depth of reads mapped to *S. cerevisiae* chromosomes and *S. uvarum* contigs.

Sc, *S. cerevisiae*; Se, *S. eubayanus*.

a *S. uvarum* chromosomes are known to have undergone reciprocal recombination [45]. We have assumed collinearity with *S. eubayanus*.

*S. pastorianus* shows a high degree of aneuploidy and the chromosomal composition between strains is highly variable. Strains DBVPG 6033, 6261 and 6257 have eight, seven and sixteen complete *S. cerevisiae* chromosomes, respectively (Table 1). They also have an estimated 14, 10 and 15 complete *S. eubayanus* chromosomes, and 9, 14 and 17 chimeric chromosomes, composed of both *S. cerevisiae* and *S. eubayanus* sequence (Table 1). The approximate number of total chromosomes in DBVPG 6033 and 6261 (Group 1) is 31 each, both roughly equal to a diploid (16×2 = 32). The total number of chromosomes in DBVPG 6257 (Group 2) is 48, which equates to a triploid (16×3 = 48). These data support previous estimates of Group 1 strains generally being diploid-derived and Group 2 strains being triploid-derived [10]. Both Group 1 strains have lost their *S. cerevisiae* copies of chromosomes VI and XII, i.e. there is no evidence of this sequence, even on a chimeric chromosome. DBVPG 6261 has additionally lost its *S. cerevisiae* chromosome III and XIV sequences, whereas DBVPG 6033 has lost its *S. cerevisiae* chromosome XI sequence. There was no detection of *S. cerevisiae* chromosome V in the Group 2 strain DBVPG 6257. All three strains of *S. pastorianus* show evidence of homologous recombination between *S. cerevisiae* and *S. eubayanus* chromosomes IV, VII, XIII and XVI. Additionally, chromosome VIII and XV are chimeric in both Group 1 strains. Chromosomes IX, X and XIV are also chimeric in Group 2 strain DBVPG 6257. Chromosomes I and VI remain largely stable, showing no evidence of *S. cerevisiae*- *S. eubayanus* recombination in any of the sequenced strains. Reciprocal recombination and inversion events could not be identified in this study since these rearrangements are copy-number neutral.

In agreement with previous analysis of these three strains of *S. pastorianus* [10], we did not detect any *S. cerevisiae* mitochondrial DNA. The restriction analysis of *COX2* in the three strains of *S. pastorianus* has indicated a *S. uvarum*-like mitochondrial sequence (data not shown), supporting the widely held notion that lager yeasts tend to inherit and/or retain only their *S. eubayanus* mitochondria [26]. The 2-micron plasmid maps to *S. cerevisiae* sequence in strains DBVPG 6033 and DBVPG 6257, but not DBVPG 6261 (Figure S1). It is unknown if any 2-micron plasmids are *S. eubayanus*-derived.

### Chromosomal Rearrangements

**Figure 2:**
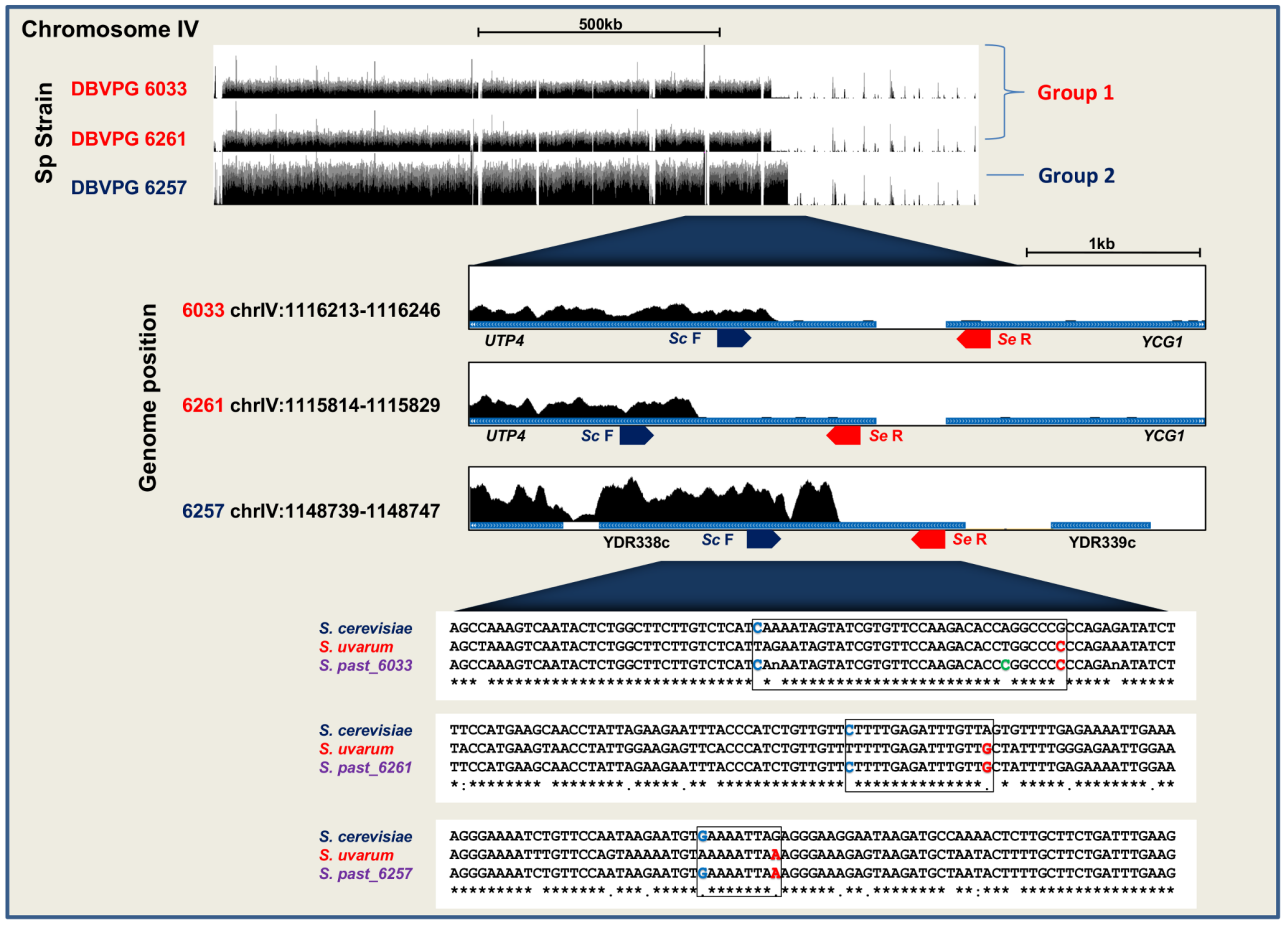
Diagram showing the experimental strategy to detect chromosomal rearrangements and the resolution at the nucleotide level of three breakpoints on chromosome IV. To establish the position of rearrangements in the three *S. pastorianus* strains (Sp), species-specific primers (Sc F: *S. cerevisiae* forward primer, Se F: *S. eubayanus* forward primer. Sc R: *S. cerevisiae* reverse primer. Se R: *S. eubayanus* reverse primer) were designed around putative breakpoints observed using the UCSC Genome Browser. The amplified products were then sequenced to locate the breakpoints at nucleotide level. *S. pastorianus* strains are labelled as Group 1 or Group 2 according to their previous assignment [10]. Nucleotide level sequence is shown for each rearrangement with the breakpoint region demarcated by a box flanked by the first unique *S. cerevisiae* nucleotide (blue) and the first unique *S. uvarum* nucleotide (red, *S. eubayanus* sequence unavailable).

We used the UCSC genome browser (http://genome.ucsc.edu/) to identify candidate breakpoints based on variations in *S. cerevisiae* read copy number across each chromosome (Figure 1). Using this technique, we were able to detect a total of 13 *S. cerevisiae- S. eubayanus* breakpoints in DBVPG 6033, 13 in DBVPG 6261 and 18 in DBVPG 6257. We used species-specific primers to confirm the presence of each breakpoint by PCR (Figure 2). Each successfully amplified PCR product was sequenced at GATC Biotech (Germany). All of the sequenced breakpoints were then aligned to the *S. cerevisiae* reference genome and either the *S. eubayanus* reference genome (where available) or the *S. uvarum* reference genome (Figure S2). A total of 9/13 *S. cerevisiae*- *S. eubayanus* breakpoints were confirmed by PCR in DBVPG 6033, another 11/13 in DBVPG 6261 and 10/18 in DBVPG 6257 (Table 2). In DBVPG 6033, three of these breakpoints were located on chromosome XVI, two on VIII and the remaining four on chromosomes II, IV, VII and XIII. In DBVPG 6261, three were located on chromosome XV, two on chromosome XI, two on chromosome XIII and the remaining four on chromosomes IV, V, VII and XVI. In DBVPG 6257, three breakpoints were located on chromosomes XVI, two on XIII and the remaining five on chromosomes IV, VII, IX, X and XI. It should be noted that the single-read sequencing strategy did not allow us to detect rearrangements that were copy number neutral (e.g. reciprocal recombination).

**Table 2:** Genomic location of *S. cerevisiae*- *S. eubayanus* breakpoints.

DBVPG strain	Genome position^a^	Systematic name	Standard name	Breakpoint location from startcodon in each chimeric gene^b^	Length ofchimeric gene^c^	Reading frame
6033	chrII:780898–780904	YBR289w	SNF5	*Sc> Se* at 1236–1242 bp	2694 bp	Intact
	chrIV:1116213–1116246	YDR324c	UTP4	*Se> Sc* at 444–477 bp	2259 bp	Intact
	chrVII:179643–179658	YGL173c	KEM1	*Se> Sc* at 462–477 bp	4587 bp	Intact
	chrVIII:433729–433738	YHR165c	PRP8	*Se> Sc* at 3222–3231 bp	7251 bp	Intact
	chrVIII:451249–451261	Intergenic	Intergenic	Intergenic*	N/A	N/A
	chrXIII:843622–843635	YMR287c	MSU1	*Se> Sc* at 1710–1723 bp	2910 bp	Intact
	chrXVI:97018–97048	YPL240c	HSP82	Se*> Sc* at 1578–1608 bp*	Incomplete**	Unknown**
	chrXVI:482999–483013	YPL036w	PMA2	*Se> Sc* at 121–134 bp	2805 bp	Intact
	chrXVI:906846–906880	Intergenic	Intergenic	Intergenic*	N/A	N/A
6261	chrIV:1115814–1115829	YDR324c	UTP4	*Se> Sc* at 861–876 bp*	2259 bp*	Intact
	chrV:507240–507255	YER164w	CHD1	*Se> Sc* at 1848–1863 bp	4401 bp	Intact
	chrVII:179643–179658	YGL173c	KEM1	*Se> Sc* at 462–477 bp	4587 bp	Intact
	chrXI:60182–60196	YKL203c	TOR2	*Sc> Se* at 3164–3179 bp	7425 bp	Intact
	chrXI:285492–285507	YKL080w	VMA5	*Sc*> *Se* at 819–834 bp	1179 bp	Intact
	chrXIII:172148–172154	YML051w	GAL80	*Se> Sc* at 555–561 bp	1308 bp	Intact
	chrXIII:882708–882717	YMR306w	FKS3	*Se> Sc* at 1551–1560 bp	5358 bp	Intact
	chrXV:496849–496867	YOR092w	ECM3	*Sc> Se* at 1722–1740 bp	1842 bp	Intact
	chrXV:526415–526427	YOR109w	INP53	*Se> Sc* at 1137–1149 bp	3324 bp	Intact
	chrXV:561420–561425	YOR127w	RGA1	*Sc> Se* at 250–255 bp	3024 bp	Intact
	chrXVI:97018–97048	YPL240c	HSP82	Se*> Sc* at 1578–1608 bp*	Incomplete**	Unknown**
6257	chrIV:1148739–1148747	YDR338c	YDR338c	*Se> Sc* at 715–723 bp	2088 bp	Intact
	chrVII:179643–179658	YGL173c	KEM1	*Se> Sc* at 462–477 bp	4587 bp	Intact
	chrIX:306348–306368	YIL026c	IRR1	*Se> Sc* at 1551–1571 bp	3444 bp	Intact
	chrX:453940–453961	YJR009c	TDH2	*Se> Sc* at 714–735 bp*	999 bp*	Intact
	chrXI:354012–354024	YKL045w	PRI2	*Sc*> *Se* at 877–888 bp	1587 bp	Intact
	chrXIII:602992–602998	YMR170c	ALD2	*Se> Sc* at 84–90 bp*	1521 bp*	Intact
	chrXIII:657834–657854	YMR196w	YMR196w	*Se> Sc* at 2790–2811 bp	3297 bp	Intact
	chrXVI:97018–97048	YPL240c	HSP82	Se*> Sc* at 1578–1608 bp*	Incomplete**	Unknown**
	chrXVI:862750–862765	YPR160w	GPH1	*Sc> Se* at 1449–1464 bp	2709 bp	Intact
	chrXVI:919949–919955	YPR191w	QCR2	*Se> Sc* at 574–579 bp	1107 bp	Intact

a Breakpoint position in genome based on *S. cerevisiae* sequence UCSC SacCer2 June 2008.

b Breakpoint region within hybrid gene indicated by the sequence overlap region (bp, base pairs) from the start of the gene. The direction of the sequence change is indicated i.e. If the gene is composed of *S. cerevisiae* (Sc) sequence before the breakpoint and *S. eubayanus* (Se) sequence after the breakpoint then the breakpoint is listed as Sc> Se (*S. cerevisiae*> *S. eubayanus*) and vice versa. Breakpoints labeled with an asterisk (*) were determined using *S. uvarum* sequence, due to the absence of available *S. eubayanus* sequence for these genes.

c Length of chimeric gene was determined by fusing the sequence of the two parental species (*S. cerevisiae* and either *S. eubayanus*, where available, or *S. uvarum* sequence) from either side of the breakpoint.

**The length of *HSP82* and the integrity of its reading frame could not be determined due to incomplete or absent parental sequence.

The majority of the sequenced *S. cerevisiae*- *S. eubayanus* breakpoints occur within coding regions (Table 2), despite breakpoints in yeast usually being located in intergenic rather than intragenic regions [27]. A total of seven, eleven and ten intragenic breakpoints were located in DBVPG 6033, 6261 and 6257 respectively. In each strain there were a small number of candidate breakpoints which could not be amplified (Table S1). Four of these unconfirmed breakpoints are in DBVPG 6033 (one of which was within in a coding region), two in DBVPG 6261 (both of which were within in a coding region) and eight in DBVPG 6257 (three of which were within a coding region). Notably, we detected a breakpoint at the MAT locus on chromosome III in strains DBVPG 6033 and 6257. Breakpoints to the right of the MAT locus in chromosome III have been noted previously in many strains of lager yeast [13], [15], [28]. There are two breakpoints that are in close proximity on chromosome X in strain DBVPG 6257, two on chromosome XIV and one on chromosome XII in strain DBVPG 6257, which are in the vicinity of Ty elements. A further three candidate breakpoints, one in each strain (at the far right of each chromosome X) could not be amplified, possibly due to their close proximity to both an AT-rich ARS element and the right telomeric region. The four remaining unsequenced candidate breakpoints are on chromosome XIII and XV in DBVPG 6033, chromosome VIII in DBVPG 6261 and chromosome XIII in DBVPG 6257. These sites of increased copy number, which we were unable to confirm, may represent amplification events rather than translocations. Since we have called the breakpoints based on the depth of *S. cerevisiae* coverage we cannot distinguish between these two events at the onset, but can confirm any true recombination events via Sanger sequencing. Furthermore, copy number changes of true non-reciprocal translocation events or gene conversion are usually matched in both subgenomes. The unamplified breakpoint on chromosome XII in DBVPG 6257 may constitute a case of amplification since there is an increase of read depth in the *S. cerevisiae* genome but no change in the *S. eubayanus* genome (Table S4). Likewise, the pair of closely located unamplified breakpoints on chromosome X in DBVPG 6257 may represent one deletion, since there are no reads mapped to this region of the *S. cerevisiae* genome, while read depths remains unchanged in the *S. eubayanus* genome. Interestingly, there are three other pairs of breakpoints that generate small gaps in the *S. cerevisiae* subgenome sequencing data (ALD2-YMR196w on chromosome XIII of DBVPG 6257; ECM3-INP52 on chromosome XV of DBVPG 6261 and PRP8-intergenic on chromosome VIII of DBVPG 6033). These may represent gene conversion from a small tract in the *S. eubayanus* to the *S. cerevisiae* subgenome rather than deletion, since each breakpoint generated a chimeric gene (Table 3).

**Table 3:** Copy number of chimeric genes and their parental homologues present in the *S. pastorianus* strains.

DBVPG strain	Chr.	Systematic name	Standard name	Chimeric	Sc	Se
6033	II	YBR289w	SNF5	1	0	1
	IV	YDR324c	UTP4	1	0	1
	VII	YGL173c	KEM1	1	1	1
	VIII	YHR165c	PRP8	1	0	1
	XIII	YMR287c	MSU1	1	0	1
	XVI	YPL240c	HSP82	2	0	0
	XVI	YPL036w	PMA2	1	0	1
6261	IV	YDR324c	UTP4	1	0	1
	V	YER164w	CHD1	3	0	0
	VII	YGL173c	KEM1	3	0	0
	XI	YKL203c	TOR2	1	0	1
	XI	YKL080w	VMA5	1	0	1
	XIII	YML051w	GAL80	1	0	1
	XIII	YMR306w	FKS3	2	1	0
	XV	YOR092w	ECM3	1	0	1
	XV	YOR109w	INP53	1	0	1
	XV	YOR127w	RGA1	1	0	1
	XVI	YPL240c	HSP82	3	0	0
6257	IV	YDR338c	YDR338c	2	0	1
	VII	YGL173c	KEM1	1	2	0
	IX	YIL026c	IRR1	3	0	0
	X	YJR009c	TDH2	1	1	1
	XI	YKL045w	PRI2	2	0	2
	XIII	YMR170c	ALD2	1	0	2
	XIII	YMR196w	YMR196w	1	0	2
	XVI	YPL240c	HSP82	3	0	0
	XVI	YPR160w	GPH1	1	0	2
	XVI	YPR191w	QCR2	3	0	0

Copy number of each chimeric gene and its *S. cerevisiae* (Sc) and *S. eubayanus* (Se) homologue is based on regional read depth analysis (Table S3 and S4) on either side of the breakpoint.

### Chimeric Genes

**Figure 3:**
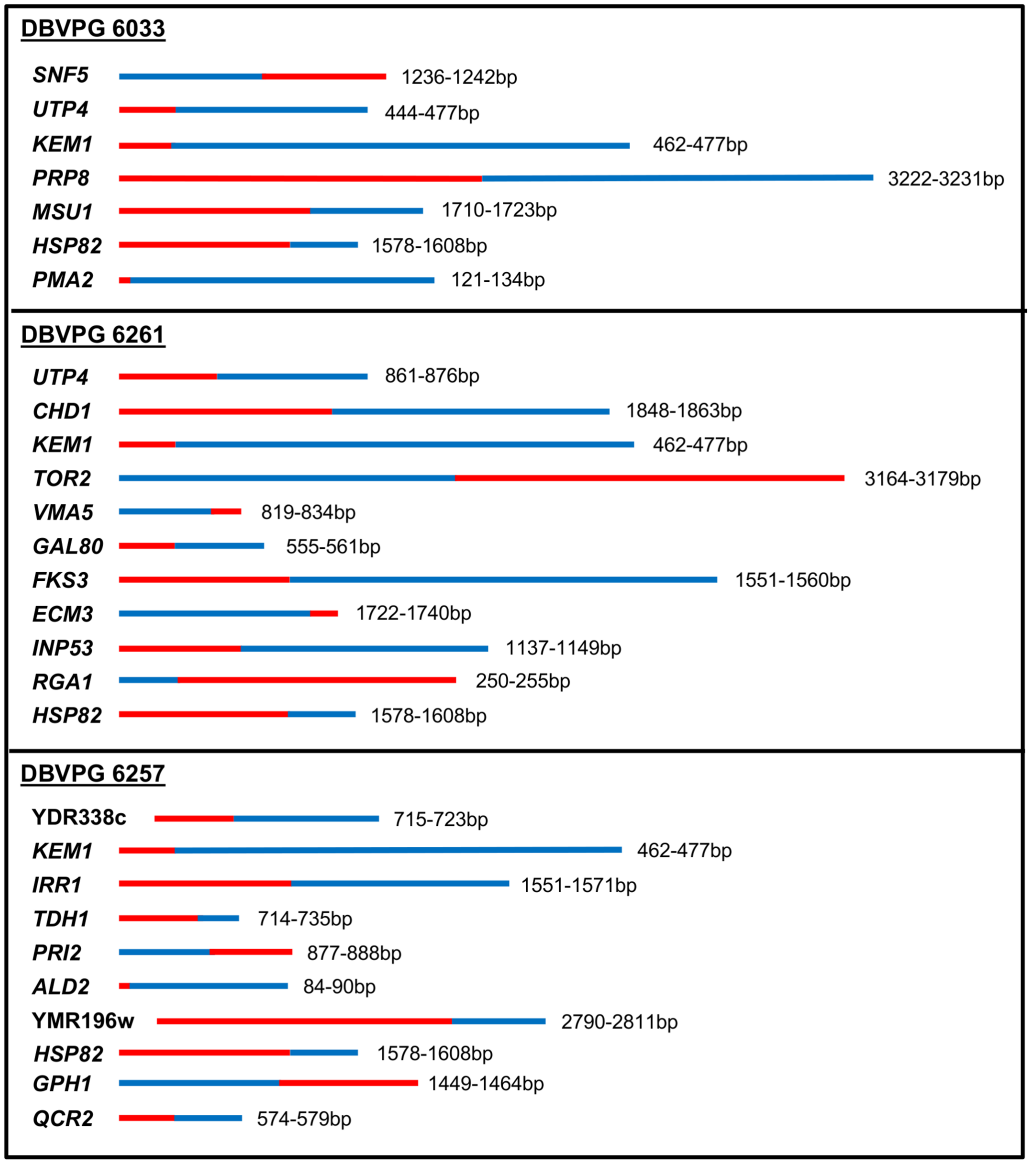
A visual representation of the chimeric genes in three strains of *S. pastorianus*. The *S. cerevisiae* and *S. eubayanus* portions of the gene are shown in blue and red respectively, and the position at which the breakpoint occurs within each gene is reported.

**Figure 4:**
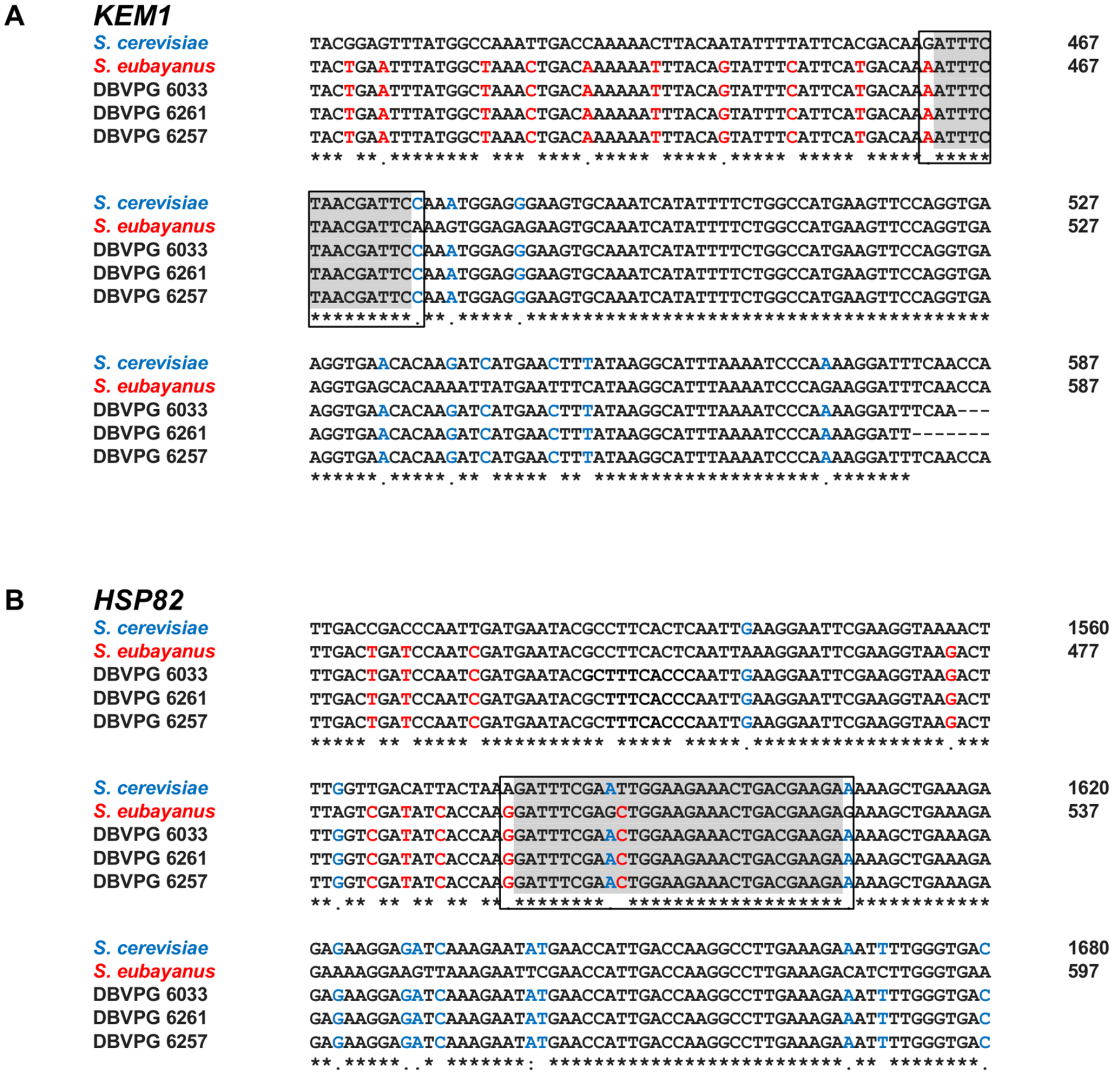
Sequence of the breakpoints within *KEM1* and *HSP82* in three strains of *S. pastorianus*. Panel A: Multiple sequence alignment of the breakpoint region within *KEM1* in three strains of *S. pastorianus* and the parental species *S. cerevisiae* and *S. eubayanus*. Panel B: Multiple alignment of the breakpoint region within *HSP82* in three strains of *S. pastorianus* and the parental species *S. cerevisiae* and *S. uvarum* (*S. eubayanus* sequence for this gene is unavailable). Unique nucleotide matches to *S. cerevisiae* are shown in blue while unique matches to *S. eubayanus* (*KEM1*) *or S. uvarum* (*HSP82*) are shown in red. The breakpoint region is demarcated by a box. Nucleotides shared between both parental species within the breakpoint region are shaded in grey.

As a result of homologous recombination between *S. cerevisiae* and *S. eubayanus* chromosomes, several chimeric genes were formed (Figure 3; Table 2; see Figure S2 for full sequence alignments). Two of these genes, *KEM1*, a 5′-3′ exonuclease, and *HSP82*, a molecular chaperone, are chimeric in all three strains. Interestingly, multiple sequence alignment using Clustal Omega showed that these two breakpoints occur in the same gene position in all three hybrids (Figure 4). The *KEM1 S. eubayanus*> *S. cerevisiae* breakpoint occurs within 462–477 bp after the start of the gene and the *HSP82 S. eubayanus*> *S. cerevisiae* breakpoint occurs within 1578–1608 bp after the start of the gene. Another chimeric gene, *UTP4*, is shared between both Group 1 strains, yet the position of the *S. eubayanus> S. cerevisiae* breakpoint differs between each strain: in DBVPG 6033, the breakpoint occurs within 444–477 bp after the start of the gene, whereas in DBVPG 6261, the breakpoint occurs within 861–876 bp after the start of the gene.

Using regional read depth data across each chromosome (Table S3 and Table S4), we investigated chimeric gene copy number and whether additional complete *S. cerevisiae* or *S. eubayanus* copies of each chimeric gene were present in the sequencing data (Table 3). There is only one copy of the majority of chimeric genes across all three strains. However, there are two copies of four chimeric genes and three copies of six chimeric genes. The majority of duplicate and triplicate genes occur towards the ends of the chromosomes (see Figure 1). These have likely formed through either gene conversion involving three chromosomes or duplication of sub-telomeric regions, which are known to be sites of rapid gene expansion [29].

There are no additional complete parental strain copies of seven of the sequenced chimeric genes (*HSP82* in all three strains, *CDH1*, *IRR1*, *KEM1* (DBVPG 6261) and *QCR2*) (Table 3). Interestingly, *IRR1* is an essential gene in *S. cerevisiae*, as well as being non-redundant. If it is presumed that *IRR1* is also essential in *S. pastorianus*, it can be deduced that *IRR1* is a functioning chimeric gene, given that this strain is viable. The remaining 21 chimeric genes have one or more non-chimeric homologues. Although only the chimeric form of *HSP82*, a molecular chaperone of protein complexes, is present in each strain of *S. pastorianus*, its homologue *HSC82* has been retained in both parental forms. Two chimeric genes, *KEM1* in DBVPG 6033 and *TDH2* in DBVPG 6257 have additional homologous copies of both *S. cerevisiae* and *S. eubayanus* genes present in the genome. A further two chimeric genes, *FKS3* and *KEM1* in DBVPG 6257 have at least one additional *S. cerevisiae* homologue remaining in the genome. The final 17 chimeric genes are complemented by at least one additional complete *S. eubayanus* homologue. The identification of chimeric gene copy number within *S. pastorianus* is of importance in phylogenetic analysis, since they have the potential to weaken phylogenetic signal and contribute to incongruence [30], [31].

Interestingly, two genes found in the chimeric form are involved in ethanol metabolism, a key biochemical pathway in lager fermentation. *ALD2* is involved in the oxidation of ethanol and *TDH2* is a component of the tetramer glyceraldehyde-3-phosphate dehydrogenase, which is required for gluconeogenesis. Two further chimeric genes also play a role in energy metabolism. *GPH1* is involved in glycogen mobilisation and *GAL80* is a repressor of GAL genes in the absence of galactose. With the large genetic redundancy in all three strains of *S. pastorianus*, especially DBVPG 6257, which contains approximately two *S. cerevisiae* sets of chromosomes, chimeric gene copies may or may not significantly affect the hybrid organism.

Previous studies on the functionality and fitness of chimeric genes show mixed results. The chimeric gene *GPH1* in the lager strain CMBS-33 contains a disruptive base insertion within its initial *S. eubayanus* sequence, and the resultant gene is not expressed [32]. However, a recent study located a recurrent in-frame breakpoint within *MEP2*, an ammonium permease, in clones of lab-created hybrids of *S. cerevisiae* and *S. uvarum* that were evolved under nitrogen-limiting conditions [33]. The experimentally evolved strains bearing the rearrangement were fitter than the non evolved strains in nitrogen-limiting competition experiments. A number of studies have also examined the fitness effects of rearrangements involving non-homologous genes. The *LG-FLO1* gene, involved in flocculation, appears to have been inactivated in non-flocculent lager yeast by a non-reciprocal translocation of *S. cerevisiae* YIL169c into its C-terminal region in various non-flocculent strains [16]. However, a sulphite-resistant gene found in wine yeast, *SSU1*-R was generated by recombination between the promoter regions of *SSU1* and *ECM34* genes and has been found to grant increased sulphite resistance compared to the wild type allele [22], [34].

### Comparison of Breakpoints in Different *S. pastorianus* Strains

**Figure 5:**
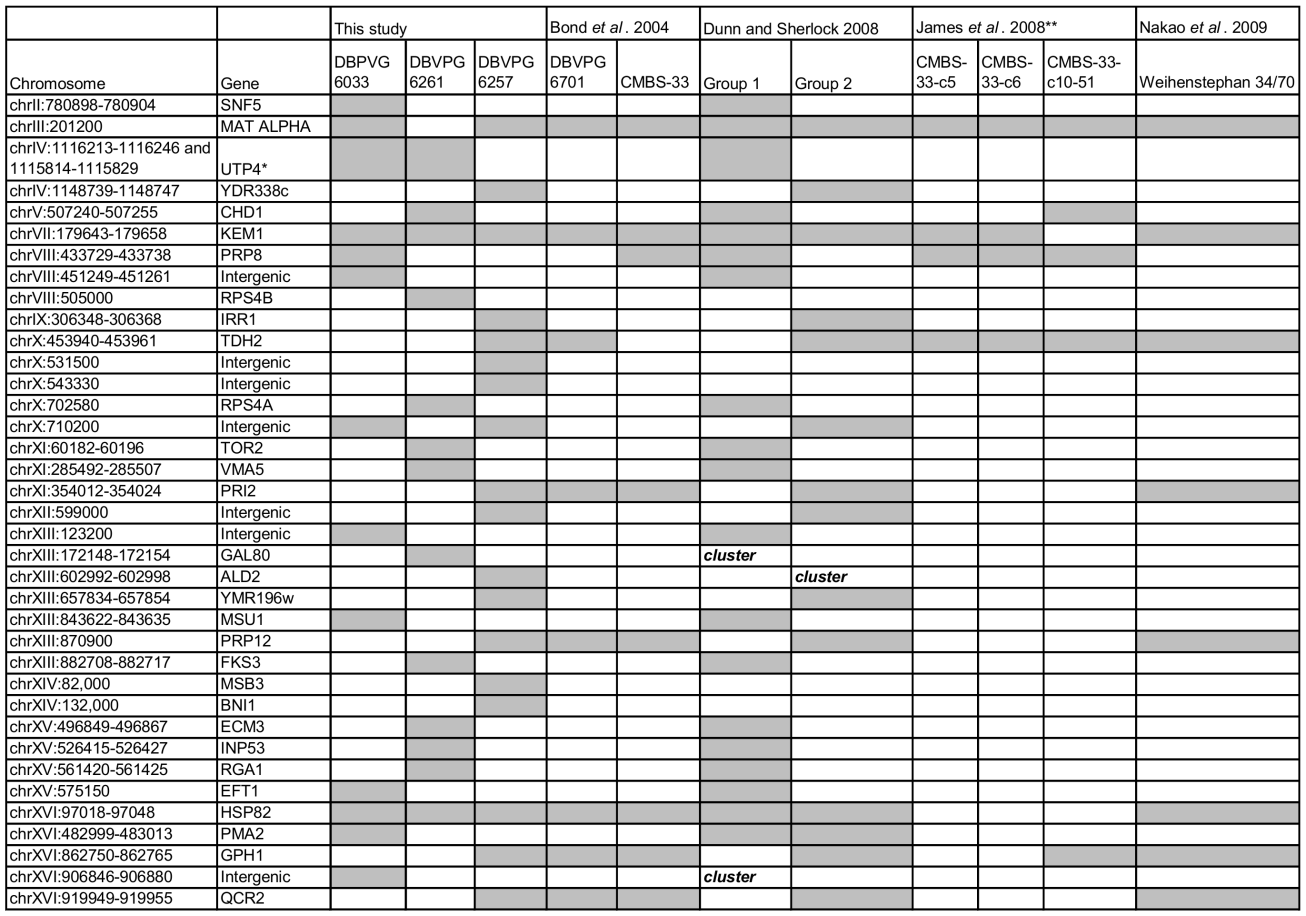
A comparison of breakpoints identified in our study with breakpoints found in other studies of *S. pastorianus*. We noted the presence of absence of similar breakpoints found in *S. pastorianus* strains analysed in four previous studies, Bond *et al*. 2004 [15], Dunn and Sherlock 2008 [10], James *et al*. 2008 [21] and Nakao *et al*. 2009 [13]. Grey shading indicates the presence of a breakpoint found within the same gene or a nearby/adjacent gene. *Breakpoints in both DBVPG 6033 and DBVPG 6261 fall within UTP4, but in differing locations. **The strains analysed in the study by James *et al*. 2008 are the product of mutagenesis and a laboratory evolution experiment. Cells labelled “cluster” within the Dunn and Sherlock (2008) analysis refer to breakpoints identified in our study which were not explicitly identified by the aforementioned study, but that fall within breakpoint cluster regions determined by the author across 17 strains of *S. pastorianus*.

Our data were compared to previous studies conducted on other *S. pastorianus* strains [10], [13], [15], [21]. The majority of the rearrangements detected in our study fall within the low resolution breakpoint regions determined previously (Figure 5). However, we also found new rearrangements in the strain DBVPG 6257 on chromosomes X, XIII and XIV; on chromosomes XVI in DBVPG 6033 and on chromosomes VIII and XIII in DBVPG 6261. A study by Bond and co-workers [15] conducted on Group 2 lager yeasts, namely DBVPG 6701 and CMBS-33, identified several breakpoints in common with the Group 2 strain DBVPG 6257 (Figure 5). However, one breakpoint unique to CMBS-33 was also found in the Group 1 strain DBVPG 6033. A previous whole genome sequencing of the Group 2 Weihenstephan 34/70 strain [13] identified a total of nine breakpoints, eight of which we also detected in our Group 2 strain DBVPG 6257. Moreover, two rearrangements, on chromosome VII (*KEM1*) and XVI (*HSP82*) were common to all the *S. pastorianus* strains analysed in our study (Figure 5). The sequencing of Weihenstephan 34/70 strain also showed a reciprocal breakpoint within *TDH2*, whereas we found altered copy number at this same site. Either there has been a chromosomal deletion in DBVPG 6257 after a reciprocal recombination event, or the original event was non-reciprocal. Furthermore, since reciprocal recombination events could not be detected in this study, it is also possible that the breakpoint may have occurred twice in this location, once as a reciprocal event and once as a non-reciprocal event.

### The Reuse of Breakpoints

The reuse of breakpoints is a relatively new hypothesis that challenges the long held random breakage model of chromosomal rearrangements [35] and is gaining momentum in studies of mammalian and fly genomes. Studies of mammalian genomes have indicated that breakpoint regions may be reused throughout evolution at a rate between 7.7% and 20% [36], [37], [38]. The term breakpoint reuse, first coined by Pevzner and Tesler [39], applies to regions of overlapping breakpoints and is not limited to breakpoints shared at the nucleotide level. Although it is unclear whether this overlap in usage is random or non-random, there is increasing evidence of association of these evolutionary breakpoint regions with fragile sites (heritable regions prone to breakage and reorganization) [39], [40], with telomeric and centromeric regions [36], with segmental duplications [36], [37], and gene dense regions [36], [38]. Moreover, fragile sites prone to breakage, rather than functional constraints on genes, are thought to have been instrumental in shaping gene organisation, at least in *Drosophila* [41]. In our study, we see the reuse of two breakpoints, *HSP82* and *KEM1* in all three sequenced strains of *S. pastorianus* (Table 2). These breakpoints may have occurred independently between the separate groups. Alternatively, there could have been genetic exchange between Group 1 and 2, although the differing geographic distributions of each group make this unlikely [10]. Mutagenized lager yeast strains selected under heat stress and high osmotic stress [21] showed a rearrangement in YGL172w, which is adjacent to *KEM1*. They also showed four other rearrangements in or around breakpoint genes that were identified in our study (Figure 5), a further indication of breakpoint reuse in these regions of the genome. Interestingly, a fully sequenced *S. cerevisiae*- *S. eubayanus* breakpoint within *GPH1*, in the lager strain CMBS-33, differs in location to a breakpoint found in the same gene in DBVPG 6257 in our study [32]. The breakpoint identified in our study occurs within 1449–1464 bp of the start of the gene, whereas the breakpoint in CMBS-33 occurs after the first 330–360 bp of the gene. Similarly in our study, a breakpoint present in both Group 1 strains in *UTP4* was resolved into two distinct breakpoints at the nucleotide level, which are in close proximity to a breakpoint approximately 30 kb upstream of this site in the ORF YDR338c of DBVPG 6257 (Table 2, Figure 2). This region on chromosome IV is potentially an additional site of independent evolutionary breakpoint reuse.

### Mechanisms of Breakpoint Formation

Chromosomal translocation requires the induction of double-stranded DNA breaks followed by incorrect repair of these breaks using an erroneous homologous and repetitive sequence [42]. A recent study found that the potential of a double stranded break in the genome to cause changes in genome copy number increases when the breakage occurs within non-repetitive DNA rather than repetitive DNA [43]. This effect was far more pronounced in hybrid diploids comparative to non-hybrid diploids. This would suggest that any breakpoint that was to randomly occur within a coding region may be more likely to promote a genomic rearrangement in *S. pastorianus* than if the break was to occur within a repetitive element.

We looked for the presence or absence of Ty elements, their flanking LTRs, tRNAs and origins of replication in proximity to each identified breakpoint. Using the sequence data mapped to each *S. cerevisiae* SacCer2 chromosome in UCSC genome browser (http://genome.ucsc.edu/), we manually recorded the nearest repetitive genomic feature to each breakpoint (Table S2). The proximity of our sequenced breakpoints to a repetitive element ranges between 0.6 kb and 39 kb with a mean of 11.4 kb. Five sequenced breakpoints were less than 5 kb from an element; eleven were between 5 and 10 kb away; eight were between 10 kb and 20 kb away and four were father than 20 kb away from a repetitive element. The majority of breakpoints, having occurred within coding regions, were not immediately flanked by repetitive elements. The lack of association between breakpoints and repetitive elements in lager yeast is in agreement with Nakao and co-workers [13] and for some of the breakpoint events studied by Bond and co-workers [15]. However, Dunn and Sherlock [10] have observed clustering of breakpoints near repetitive features in the genomes of lager yeast. It is possible that our sequencing strategy was unable to fully detect any breakpoints that may have occurred within repetitive regions. Furthermore, our analysis of the location of these elements is based on SacCer2, and not the *S. cerevisiae* progenitor strain, of which we have no information. Additionally, we do not have data concerning the distribution of *S. eubayanus* repetitive elements and we could not accurately assess the locality of sacBay MIT (*S. uvarum*) transposons, since this portion of the data is mapped only to contigs. Despite the lack of proximity of repetitive elements to breakpoints as a trend, one breakpoint gene, *TDH2* on chromosome XII in DBVPG 6257 is situated adjacent to an ARS, a feature known to promote chromosomal translocation [20]. Additionally we noticed a *S. uvarum* transposon present on a contig at the site of one breakpoint on chromosome XIII (Table S1).

Large areas of homology are known to induce recombination in yeast, and this mechanism is utilised widely for yeast gene deletion in the laboratory [44]. More recently however, very small areas of microhomology have also been indicated in the formation of chromosomal breakpoints in wine yeast [3], [22]. Since the reference parental species of *S. pastorianus* are closely related, with an average of 80% nucleotide identity in coding regions [45], we view the induction of recombination via homologous regions in lager yeast a likely hypothesis. Furthermore recombination has occurred more frequently in these coding regions than in non-coding regions, which have an average nucleotide identity of only 62% [45]. We examined the sequence surrounding each breakpoint using multiple alignments to the two parental subgenomes (Figure S2) and identified many cases of local large areas of identity and smaller areas of microhomology that are at the site of each sequenced breakpoint (Table S2).

Whatever the underlying sequence that facilitates breakpoint formation, it is likely that one or all of the following three events is potentiating breakpoint formation: the unstable nature of newly formed hybrids, an increase in the occurrence of double stranded breaks under stressful brewery conditions and/or an evolutionary pressure for recombination. The reoccurrence of known breakpoints in mutagenized lager strains which have been evolved under high stress brewery conditions [21] promotes the existence of breakpoint hotspots in the *S. pastorianus* genome and gives evidence for the role of stress in promoting and maintaining genomic breakpoints.

### Conclusion

Our whole genome sequencing of three strains of *S. pastorianus* allowed the identification of *S. cerevisiae*- *S. eubayanus* chromosomal breakpoints at a single nucleotide resolution. The majority of *S. cerevisiae*- *S. eubayanus* breakpoints are located within coding regions and were most likely formed as a result of homology and microhomology between the two parental subgenomes, rather than via repetitive elements in the genome. PCR sequencing of breakpoints enabled the further characterisation of these recombination-generated chimeric genes. The greater resolution granted by PCR sequencing allowed us to verify that the breakpoints within *HSP82* and *KEM1* have occurred at an identical genomic location in all three strains. We determined that two different breakpoints have occurred within *UTP4* in the two Group 1 strains. Although the breakpoints are in different positions, this will still be regarded as an example of breakpoint reuse. Interestingly, we note the presence of a chimeric gene *IRR1* in DBVPG 6257 of *S. pastorianus* that has lost both parental homologues. Since *IRR1* is also an essential gene, this indicates that the chimeric gene is efficiently utilised by the hybrid. The presence of chimeric genes in the genome also has the potential to weaken the phylogenetic signal of these genes, which could promote incongruence in phylogenetic analyses [31]. Future studies on the function and fitness of chimeric genes may reveal their evolutionary role in facilitating the adaption of *S. pastorianus* to high stress brewery conditions.

## Materials and Methods

### Strains and Media

*Saccharomyces pastorianus* strains DBVPG 6033 (GSY129), DBVPG 6261 (GSY134) and DBVPG 6257 (GSY132) were obtained from DBVPG Industrial Yeasts Collection, University of Perugia, Italy. Yeast was grown at 25°C, 200 rpm for 20 hours in YPD (1% yeast extract, 2% peptone, 2% glucose) and genomic DNA extracted using Wizard Genomic DNA Purification Kit (Promega).

Genome sequencing reference strain for *Saccharomyces cerevisiae,* sacCer2 was obtained via the UCSC Genome Browser (http://genome.ucsc.edu/). Genome sequencing reference strain for *Saccharomyces uvarum*, sacBay MIT, was obtained from the *Saccharomyces* Genome Database (SGD, http://www.yeastgenome.org).

### SOLiD Sequencing

The genomic DNA of three strains of *S. pastorianus* was sequenced using Next Generation Sequencing Applied Biosystems SOLiD 4 platform to generate 50 bp single-end reads. Using BFAST (http://sourceforge.net/projects/bfast/files/), the reads were mapped to the *S. cerevisiae* reference genome “sacCer2” obtained from UCSC (http://genome.ucsc.edu/), which includes 16 chromosomes, the mitochondrial genome and the 2 micron plasmid. The ‘-a 3’ flag of the post-process step was used to obtain unique best scoring alignments. The *S. cerevisiae* ORFs were used to find *S. eubayanus* consensus ORFs in the *S. eubayanus* reference strain “sacBay MIT” obtained from SGD (http://www.yeastgenome.org/). BFAST files were filtered to retrieve sets of reads with 0, 0–1 or 0–5 mismatches to each reference genome. Generally, 0 mismatches was found to be the most suitable cut-off value for the *S. cerevisiae* data and 0–5 mismatches for the *S. eubayanus* data, having the best agreement to previous microarray data by Dunn and Sherlock (2008).

### Chromosomal Copy Number Analysis and Breakpoint Identification

We used the *S. pastorianus* SOLiD sequence mapped to the pre-annotated *S. cerevisiae* genome sequence via the UCSC Genome Browser (http://genome.ucsc.edu/) to identify both *S. cerevisiae* chromosome copy number and potential chimeric chromosomes comprising both *S. cerevisiae* and *S. eubayanus* sequence. These candidate breakpoint regions were identified visually by their abrupt and sustained reduction in *S. cerevisiae* reads along a chromosome. Due to difficulties in mapping and analysing repetitive regions, telomeres were excluded from the analysis. Similarly, changes in read number due to the presence of a yeast transposon (Ty) or other repetitive element were excluded.

To estimate chromosomal copy number we first broke down each genome into regions. The *S. cerevisiae* genome was broken down into 26 regions (labeled in Figure 1) fully covering either side of each breakpoint based on the mapping to the UCSC Genome Browser (Table S3). Since *S. eubayanus* genomes is only present in contigs, we chose a representative selection of 47 regions across each chromosome to sample regions either side of each breakpoint observed in the mapped *S. cerevisiae* data (Figure 1, Table S4). Pre-existing rearrangements between chromosomes within the *S. uvarum* genome (chromosomes II–IV, VI–X and VIII–XV) [45] were taken into account when estimating copies of *S. eubayanus* chromosomes. We have assumed the *S. eubayanus* genome to be collinear with the highly related *S. uvarum* genome. The ‘coverage’ tool from the Bedtools suite of programs (http://bedtools.readthedocs.org/en/latest/) was used to obtain the read depth for every position in each of the regions under consideration. The analysis used the mapped reads at a cutoff of 0 mismatches for the *S. cerevisiae* data and 0–5 mismatches for the *S. eubayanus* data as previously described. For each region the median depth of coverage values were calculated using a custom Peal script excluding 0 coverage values.

To ascertain copy number of each *S. cerevisiae* and *S. eubayanus* regions the read depth data of each region were grouped into clusters using hierarchical cluster analysis software [45]. The analyses were performed separately for each strain of *S. pastorianus* using Ward’s method to generate read depth clusters which are displayed as dendrograms (Figure S3). Using the mean depth of the regions within each cluster the copy number was then assigned (Table S3 and Table S4). The regional copy numbers, in conjunction with breakpoint data, was used to calculate *S. cerevisiae*, *S. eubayanus* and chimeric chromosomal copy number.

### PCR Amplification and Sequencing of Breakpoints

We used the *S. pastorianus* SOLiD sequence mapped to the pre-annotated *S. cerevisiae* genome sequence via the UCSC Genome Browser (http://genome.ucsc.edu/) to identify potential chimeric chromosomes comprising both *S. cerevisiae* and *S. eubayanus* sequence. These candidate breakpoint regions were identified by their abrupt and sustained reduction in *S. cerevisiae* read depth along a chromosome. Species-specific primers were designed to flank each predicted breakpoint area. The *S. cerevisiae* primers were designed using *S. cerevisiae* sequence obtained directly from the UCSC Genome Browser (http://genome.ucsc.edu/). The *S. eubayanus* primers were designed by finding the *S. uvarum* orthologue of the nearest *S. cerevisiae* gene using the SGD Synteny Viewer (http://www.yeastgenome.org). This orthologue was then mapped to *S. pastorianus* to find consensus sequences for the *S. eubayanus* portion of the *S. pastorianus* genome. The *S. eubayanus*-specific primer was then designed within this consensus sequence. Candidate primers were generated for both *S. cerevisiae* and *S. eubayanus* sequences using Primer 3 (http://frodo.wi.mit.edu). To circumvent the potential for non-specific binding between the two closely related subgenomes, these primers were then carefully selected for species*-*specificity using the Fungal BLAST tool in SGD (http://www.yeastgenome.org). For ease of amplification, primers were designed to anneal no more than a few thousand base pairs apart but with sufficient sequence either side of the breakpoint for clear identification of each subgenome. Primer sequences are available in Table S5.

PCR conditions were optimised for each breakpoint to obtain pure homogeneous chimeric sequence. The PCR product was separated by electrophoresis on 1% (w/v) agarose gel. PCR products were purified prior to sequencing using QIAquick PCR Purification Kit (Qiagen, UK). The purified PCR products were Sanger sequenced at GATC Biotech (Germany).

### Multiple Alignment of Sanger Sequenced Breakpoints to Parental Subgenomes

Each sequence covering each breakpoint in each *S. pastorianus* strain was aligned to *S. cerevisiae* (Scer S288C, Saccharomyces Genome Database) and *S. eubayanus* (FM318, http://hittinger.genetics.wisc.edu/index.html), where available, or *S. uvarum* (MIT_Sbay or WashU_Sbay, Saccharomyces Genome Database) using Clustal Omega (http://www.clustal.org/). Low quality ends of breakpoint sequences were trimmed before alignment. The breakpoint region in each sequence was determined as the area of identical nucleotides between the parental species flanked by *S. cerevisiae*-like sequence on one side and *S. eubayanus*-like sequence on the other side of the region. The start of *S. cerevisiae*-like sequence was determined by nucleotide match to *S. cerevisiae* but mismatch to either *S. eubayanus* or *S. uvarum*. The start of *S. eubayanus*-like sequence was determined by nucleotide match to *S. eubayanus* or *S. uvarum* but mismatch to *S. cerevisiae*.

### Analysis of Chimeric Gene Length and Reading Frame

The proposed length of each chimeric gene was determined by merging *S. cerevisiae* sequence and *S. eubayanus* or *S. uvarum* sequence at the junction of the pre-determined breakpoint region. Reading frame was checked using Expasy Translate (http://web.expasy.org/translate/).

### Analysis of Sequence Identity

Percentage nucleotide identity between each subgenome and *S. pastorianus* was calculated using *S. cerevisiae* and either *S. eubayanus* (obtained from http://hittinger.genetics.wisc.edu/, where available) or *S. uvarum* sequences obtained from SGD (http://www.yeastgenome.org/) and Clustal Omega (http://www.ebi.ac.uk/Tools/msa/clustalo/) Amino acid identity was calculated similarly. Chimeric nucleotide sequences were first converted to protein sequences using Expasy Translate Tool (http://web.expasy.org/translate/).

### Data Deposition

Raw reads from this study have been deposited at the European Nucleotide Archive under the accession number PRJEB4654 at http://www.ebi.ac.uk/ena/data/view/PRJEB4654; Sanger sequenced reads covering the breakpoint regions have been submitted to the European Nucleotide Archive under the accession numbers HG803141–HG803169 at http://www.ebi.ac.uk/ena/data/view/HG803141-HG803169.

## Supporting Information

Figure S1 **Mapping of the 2-micron plasmid to** ***S. cerevisiae*** **sequence.** The 2-micron plasmid DNA from each strain of *S. pastorianus* is mapped to *S. cerevisiae* sequence using the UCSC Genome Browser. The scale on the Y axis is capped at a read depth of 4500.
(TIF)

Figure S2 **Multiple alignment of each** ***S. pastorianus*** **breakpoint sequence to the parental species.** The region sequenced over each breakpoint in each *S. pastorianus* strain was aligned with *S. cerevisiae (*Scer, *Saccharomyces* Genome Database) and either *S. eubayanus* (FM318, http://hittinger.genetics.wisc.edu/index.html) or *S. uvarum* (MIT_Sbay or WashU_Sbay, *Saccharomyces* Genome Database) ORF sequences, using Clustal Omega. The two intergenic breakpoints were aligned using nucleotide sequence upstream or downstream from the nearest ORF. The ORF sequences obtained from the *Saccharomyces* Genome Database are taken from Cliften *et al.* [46] and Kellis *et al*. [45]. Any low quality ends of each breakpoint sequence were trimmed before alignment. Breakpoint area is demarcated by underlined sequence. Nucleotides shared between both parental species are highlighted in grey and are flanked by the first unique *S. cerevisiae* nucleotide (shown in blue) and the first unique *S. eubayanus* or *S. uvarum* nucleotide (shown in red). All gene sequences are 5′-3′.
(PDF)

Figure S3 **Hierarchical cluster analysis of read depth of** ***S. pastorianus*** **chromosomes.** The median read depth for 26 regions covering the *S. cerevisiae*-like chromosomes (shown in Figure 1) and 47 regions across the *S. eubayanus*-like chromosomes (sample contigs across each chromosome) were clustered independently and for each strain of *S. pastorianus*. Results from each hierarchical cluster analysis using Ward’s method are shown as a dendrogram. The blue boxes indicate the copy number assigned to that cluster.
(PDF)

Table S1 **Breakpoints which were not successfully amplified by PCR.**
(DOC)

Table S2 **Analysis of the breakpoint region in each strain of** ***S. pastorianus*** **.**
(DOC)

Table S3 **Analysis of the copy number of** ***S. cerevisiae*** **-derived chromosomes in each strain of** ***S. pastorianus*** **using median read depth across multiple regions.**
(XLSX)

Table S4 **Analysis of the copy number of** ***S. eubayanus*** **-derived chromosomes in each strain of** ***S. pastorianus*** **using median read depth across multiple regions.**
(XLSX)

Table S5 **Primer sequences for amplification of breakpoint regions.**
(DOC)
