# Supplementary figures and images for: Sequencing and Characterisation of Rearrangements in Three S. pastorianus Strains Reveals the Presence of Chimeric Genes and Gives Evidence of Breakpoint Reuse
Source: PLoS One. 2014 Mar 18;9(3):e92203. doi: 10.1371/journal.pone.0092203 (PMC3958482; doi:10.1371/journal.pone.0092203)

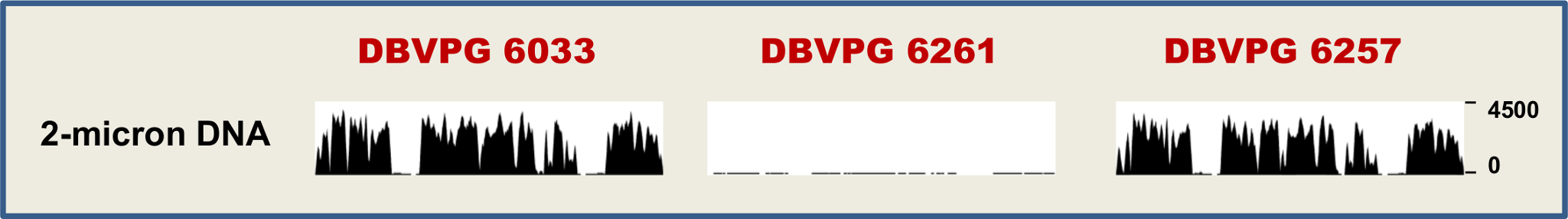

Supplement: Figure S1 — Mapping of the 2-micron plasmid to S. cerevisiae sequence. The 2-micron plasmid DNA from each strain of S. pastorianus is mapped to S. cerevisiae sequence using the UCSC Genome Browser. The scale on the Y axis is capped at a read depth of 4500. (TIF) [file pone.0092203.s001.tif]
