# Supplementary material for: Sequencing and Characterisation of Rearrangements in Three S. pastorianus Strains Reveals the Presence of Chimeric Genes and Gives Evidence of Breakpoint Reuse
Source: PLoS One. 2014 Mar 18;9(3):e92203. doi: 10.1371/journal.pone.0092203 (PMC3958482; doi:10.1371/journal.pone.0092203)
Supplement: Figure S2 — Multiple alignment of each S. pastorianus breakpoint sequence to the parental species. The region sequenced over each breakpoint in each S. pastorianus strain was aligned with S. cerevisiae (Scer, Saccharomyces Genome Database) and either S. eubayanus (FM318, http://hittinger.genetics.wisc.edu/index.html) or S. uvarum (MIT_Sbay or WashU_Sbay, Saccharomyces Genome Database) ORF sequences, using Clustal Omega. The two intergenic breakpoints were aligned using nucleotide sequence upstream or downstream from the nearest ORF. The ORF sequences obtained from the Saccharomyces Genome Database are taken from Cliften et al. [46] and Kellis et al. [45]. Any low quality ends of each breakpoint sequence were trimmed before alignment. Breakpoint area is demarcated by underlined sequence. Nucleotides shared between both parental species are highlighted in grey and are flanked by the first unique S. cerevisiae nucleotide (shown in blue) and the first unique S. eubayanus or S. uvarum nucleotide (shown in red). All gene sequences are 5′-3′. (PDF) [file pone.0092203.s002.pdf]

DBVPG 6033

SNF5/YBR289w, *Sc> Se* at 1236-1242bp

```
SGD_Scer_SNF5/YBR289W    CCAACTATTGGCCAACTTCCTCAACTTCCAAAATTAAACTTACCCAAGTACCAAACTATT    936
FM1318/1-2685            CCTACTGTTGGACAACTTCCTCAATTACCTAAGCTAAACTTACCTAAGTTTCAAACGATT    927
6033_SNF5                -------------------------------AATTAAACTTACCCAAGTACCAAACTATT    29
                                                        *. ********** ****: ***** ***

SGD_Scer_SNF5/YBR289W    CAATACGATCCACCAGAAACCAAGCTACCATATCCAACCTATTGGTCAGACAAAAAAGCA    996
FM1318/1-2685            CAGTACGACCCACCGGAATCCAAGTTACCATACCCAACGTATTGGTCAGATAAAGGAGCA    987
6033_SNF5                CAATACGATCCACCAGAAACCAAGCTACCATATCCAACCTATTGGTCAGACAAAAAAGCA    89
                         **.***** *****.***:***** ******* ***** *********** ***..****

SGD_Scer_SNF5/YBR289W    GATACGGATACTTTGTTGTACGAACAAATTATCCAGCGTGATAAAATTAACAAATATTCG    1056
FM1318/1-2685            GACACAGATACTTTATTGTATGAACAAATAATTCAGCGTGACAAAATTAACAAGTTCTCC    1047
6033_SNF5                GATACGGATACTTTGTTGTACGAACAAATTATCCAGCGTGATAAAATTAACAAATATTCG    149
                         ** **.********.***** ********:** ******** ***********.*: **

SGD_Scer_SNF5/YBR289W    CTAATAAGAGAAACCAATGGTTACGATCCGTTTAGCATTTATGGATTTAGTAATAAAGAG    1116
FM1318/1-2685            TTAGTGAGGGAAACTAACGGTTACGATCCATTCAGTATTTATGGATTCAGTAATAAAGAA    1107
6033_SNF5                CTAATAAGAGAAACCAATGGTTACGATCCGTTTAGCATTTATGGATTTAGTAATAAAGAG    209
                          **.*.**.***** ** ***********.** ** *********** ***********.

SGD_Scer_SNF5/YBR289W    TATATTAGTAGACTGTGGCATACACTGAAGTATTATCAAGATTTGAAGAACACTAGAATG    1176
FM1318/1-2685            TATATAAGCAGACTGTGGCACACATTAAAATATTATCAGGACTTAAAAAACACCAGGATG    1167
6033_SNF5                TATATTAGTAGACTGTGGCATACACTGAAGTATTATCAAGATTTGAAGAACACTAGAATG    269
                         *****:** *********** *** *.**.********.** **.**.***** **.***

SGD_Scer_SNF5/YBR289W    AAATCTATCACAAGCACTTCTCAGAAGATTCCTTCGGCAAGTATTTGGGGAAATGGTTAC    1236
FM1318/1-2685            AAATCTATAACTAACACCTCACAGAAGATTTCATCAGCAAGTATCTGGGGGAATGGTTAT    1227
6033_SNF5                AAATCTATCACAAGCACTTCTCAGAAGATTCCTTCGGCAAGTATTTGGGGAAATGGTTAC    329
                         ********.**:*.*** **:********* *:**.******** *****.********

SGD_Scer_SNF5/YBR289W    TCAGGGTATGGTAATGGGATTACGAATACAACTACCAGAGTTATTCCACAAGTAGAAGTT    1296
FM1318/1-2685            TCAGGATACGGGAACGGAATCACAAATACAACCACAAGAGTTATACCTCAAGTTGAAGTT    1287
6033_SNF5                TCAGGATACGGGAACGGAATCACAAATACAACCACAAGAGTTATACCTCAAGTTGAAGTT    389
                         *****.** ** ** **.** **.******** **.********:**:*****:******

SGD_Scer_SNF5/YBR289W    GGAAATAGGAAGCATTACCTAGAGGATAAATTAAAAGTCTATAAACAGGCCATGAATGAG    1356
FM1318/1-2685            GATAATAGAAAACACTACCTTGAAGATAAGTTGAGAGTTTATAAACAGGCAATGAGTGAA    1347
6033_SNF5                GATAATAGAAAACACTACCTTGAAGATAAGTTGAGAGTTTATAAACAGGCAATGAGTGAA    449
                         *.:*****.**.** *****:**.*****.**.*.*** ***********.****.***.

SGD_Scer_SNF5/YBR289W    ACATCGGAACAGTTAGTTCCCATAAGATTGGAGTTCGATCAAGATCGTGACAGATTCTTC    1416
FM1318/1-2685            ACAACAGAGGAATTGGTTCCGATAAGATTGGAATTCGACCAAGATCGTGACAAATTCTTC    1407
6033_SNF5                ACAACAGAGGAATTGGTTCCGATAAGATTGGAATTCGACCAAGATCGTGACAAATTCTTC    509
                         ***:*.**. *.**.***** ***********.***** *************.*******

SGD_Scer_SNF5/YBR289W    CTCAGGGACACTTTGTTATGGAACAAAAATGACAAGCTTATTAAAATTGAAGACTTTGTG    1476
FM1318/1-2685            CTAAGAGATACATTGCTATGGAACAAAAATGATGAACTTATCAAGATTGAAGAATTTGTG    1467
6033_SNF5                CTAAGAGATACATTGCTATGGAACAAAAATGATGAACTTATCAAGATTGAAGAATTTGTG    569
                         **.**.** **:*** **************** .*.***** **.********.******

SGD_Scer_SNF5/YBR289W    GACGACATGTTGCGAGATTACCGATTTGAGGACGCTACGAGAGAGCAACACATTGATACT    1536
FM1318/1-2685            GATGACATGATGCGAGATTATCGATTTGAAGATTCGACCAGGGAGCAACACATAGATACC    1527
6033_SNF5                GATGACATGATGCGAGATTATCGATTTGAAGATTCGACCAGGGAGCAACACATAGATACC    629
                         ** ******:********** ********.**  * ** **.***********:*****

SGD_Scer_SNF5/YBR289W    ATTTGTCAATCTATACAAGAGCAGATTCAGGAGTTTCAAGGAAATCCATATATAGAGTTG    1596
FM1318/1-2685            ATATGTCAGTCCATACAAGAACAAATCCAAGAATTTCAAGGAAATCCATATTTAGAATTG    1587
6033_SNF5                ATATGTCAGTCCATACAAGAACAAATCCAAGAATTTCAAGGAAATCCATATTTAGAATTG    689
                         **:*****.** ********.**.** **.**.******************:****.***

SGD_Scer_SNF5/YBR289W    AATCAGGACCGTCTAGGCGGTGATGACTTGAGAATTAGAATCAAGCTGGATATTGTCGTG    1656
FM1318/1-2685            AATCAGGATCGCTTGGGTGGTGATGATTTACGAATTAGAATCAAGCTGGATATTGTGGTG    1647
6033_SNF5                AATCAGGATCGCTTGGGTGGTGATGATTTACGAATTAGAATCAAGCTGGATATTGTGGTG    749
                         ******** **  *.** ******** **..************************* ***

SGD_Scer_SNF5/YBR289W    GGACAAAACCAGTTAATCGATCAATTTGAGTGGGACATCTCTAATAGTGATAACTGTCCA    1716
FM1318/1-2685            GGACAAAACCAACTTATCGATCAATTTGAATGGGATATCTCTAATAGTGATAATTGTCCC    1707
6033_SNF5                GGACAAAACCAACTTATCGATCAATTTGAATGGGATATCTCTAATANTGATAATTGTCCC    809
                         ***********. *:**************.***** ********** ****** *****.
```

```
SGD_Scer_SNF5/YBR289W      GAAGAGTTTGCAGAGTCCATGTGTCAAGAATTAGAACTACCAGGTGAGTTTG--TGACTG      1774
FM1318/1-2685              GAAGAATTTGCAGAATCCATGTGCCAAGAACTGGAATTGCCCGGTGAGTT--TGTTACAT      1765
6033_SNF5                  GAAGAATTTGCAGAATCCNTGTGCCCAGAACTGGAATTGCCCGGGTGAGTTCNNTTACNT      869
                           *****.*********.*** **** *.**** *.*** *.**.**   .. *    * **

SGD_Scer_SNF5/YBR289W      CCATTGCTCACTCCATAAGAGAGCAAGTTCATATGTATCATAAATCACTGGCACTGTTAG      1834
FM1318/1-2685              CCATTGCACACTCGATAAGAGAACAGGTTCACATGTACCACAAATCGCTGGCATTGTTAG      1825
6033_SNF5                  CCATTGCACACTCGATAAGAGAACAGGTTCACATGTAC----------------------      907
                           *******:***** ********.**.***** *****
```

UTP4/YDR324c, *Se> Sc* at 444-477bp *

```
SGD_Scer_UTP4/YDR324C      ACTGTGGTAACAGAATGGGATTTAGCAACAGGTTTACCATTAAGAAACTATGATTGCAAT      420
MIT_Sbay_c442_4963         ACTGTCGTTACAGAATGGGACTTGGCCACAGGTTTACCGTTAAGAAATTACGACTGTAAT      348
UTP4                       -------------AATGGGAcTTGGcTACGGGTTTACCATTAAGAAATTATGACTgTAAT      47
                                        ******* **.*  **.********.******** ** ** *  ***

SGD_Scer_UTP4/YDR324C      TCAGGTGTGATATGGTCTATTTCCATCAACGATTCACAAGACAAGCTGTCTGTGGGTTGC      480
MIT_Sbay_c442_4963         GCAGGTGTGATCTGGTCTATTGCCATCAACGATTCCCAAGATAAACTGTCTGTGGGTTGC      408
6033_UTP4                  GCAGGCGTGATCTGGTCTATTGCCATAAATGAAtCCCAAGATAAGCTGTCAGTGGGCTGC      107
                            **** *****.********* ****.** **: *.***** **.*****:***** ***

SGD_Scer_UTP4/YDR324C      GATAATGGGACGGTGGTCCTCATAGATATCTCTGGCGGGCCTGGTGTCTTGGAACACGAT      540
MIT_Sbay_c442_4963         GATAATGGGACTGTTGTACTTATAGATATTTCTGGGGGGCCAGGTGTCTTGGAACACGAT      468
6033_UTP4                  GATAATGGGACTGTGGTACTTatAGATAtntCTGgGGGGCCGGGTGTCTTGGAACACGAT      167
                           *********** ** **.**   *****   ***  ***** *****************

SGD_Scer_UTP4/YDR324C      ACTATTTTGATGAGACAAGAAGCCAGAGTATTGACTTTGGCTTGGAAAAAGGATGACTTC      600
MIT_Sbay_c442_4963         ACTATTCTAATGAGACAAGAAGCCAGAGTATTGACTTTAGCTTGGAAGAAGGATGACTTT      528
6033_UTP4                  ACTATTnTGATGAGACAAGAAGCCAGAGTATTGACTTTGGCTTGGAAAAAGGATGACTTC      227
                           ****** *.*****************************.********.***********

SGD_Scer_UTP4/YDR324C      GTGATTGGTGGTTGTTCTGATGGTAGAATAAGGATTTGGTCTGCACAAAAAAAATGACGAA      660
MIT_Sbay_c442_4963         GTTATTGGTGGTTGTTCTGATGGTAGAATAAGGATTTGGTCGGCTCAAAAGGGTGAAGAA      588
6033_UTP4                  GTGATTGGTGGTTGTTCTGATGGTAGAATAAGGATTTGGTCTGCACAAAAAAAATGACGAA      287
                           ** ************************************** **:*****...***.***

SGD_Scer_UTP4/YDR324C      AACATGGGTCGTCTATTACACACTATGAAGGTCGACAAGGCCAAAAAAGAATCAACTCTA      720
MIT_Sbay_c442_4963         AACATGGGCCGTCTATTGCATACCATGAAAGTCGACAAAGCCAAGAGAGAATCAACTTTA      648
6033_UTP4                  AACATGGGTCGTCTATTACACACTATGAAGGTCGACAAGGCCAAAAAAGAATCAACTCTA      347
                           ******** ********.** ** *****.********.*****.*.********** **

SGD_Scer_UTP4/YDR324C      GTTTGGTCAGTTATATATTTACCAAGAACTGATCAGATTGCCTCTGGTGATTCTACAGGC      780
MIT_Sbay_c442_4963         GTTTGGTCCGTTATCTATTTGCCAAATACTGATCAAATCGCATCTGGTGATTCTACCGGC      708
6033_UTP4                  GTTTGGTCAGTTATATATTTACCAAGAACTGATCAGATTGCCTCTGGTGATTCTACAGGC      407
                           ********.*****.*****.****.:********.** ** **.***************.***

SGD_Scer_UTP4/YDR324C      TCCATTAAATTCTGGGATTTCCAGTTTGCCACGCTAAACCAGTCATTTAAGGCGCACGAT      840
MIT_Sbay_c442_4963         TCCATAAAGTTCTGGGATTTCCAATTCGCTACATTGAACCAGTCTTTTAAAGCACATGAT      768
6033_UTP4                  TCCATTAAATTCTGGGATTTCCAGTTTGCCACGCTAAACCAGTCATTTAAGGCGCACGAT      467
                           *****:**.**************.** ** ** .  *.********:*****.**.** ***

SGD_Scer_UTP4/YDR324C      GCAGACGTACTGTGTCTAACTACCGATACTGATAATAATTATGTTTTTAGTGCTGGTGTG      900
MIT_Sbay_c442_4963         GCAGACGTCTTATGCCTAACTACTGATATTGATAACAATTACGTGTTCAGTGCTGGTGTG      828
6033_UTP4                  GCAGACGTACTGTGTCTAACTACCGATACTGATAATAATTATGTTTTTAGTGCTGGTGTG      527
                           ********.  *.** ******** **** ****** ***** ** ** ************

SGD_Scer_UTP4/YDR324C      GACAGAAAAATCTTTCAATTTTCTCAAAACACTAACAAATCTCAAAAGAACAACAGATGG      960
MIT_Sbay_c442_4963         GATAGAAAAATCTTCCAATTCTCCCAAAATAGCAACAAATCTCAAAAAAACAACAGATGG      888
6033_UTP4                  GACAGAAAAATCTTTCAATTTTCTCAAAACACTAACAAATCTCAAAAGAACAACAGATGG      587
                           ** *********** ***** ** *****  *   ***************.************

SGD_Scer_UTP4/YDR324C      GTAAATTCTTCTAATAGGTTGCTTCATGGAAACGACATTAGAGCAATATGTGCATACCAA      1020
MIT_Sbay_c442_4963         GTGAACTCTTCCAATAGGTTACTTCATGGTAACGAA------------------------      924
6033_UTP4                  GTAAATTCTTCTAATAGGTTGCTTCATGGAAACGACATTAGAGCAATATGTGCATACCAA      647
                           **.** ***** ********.********.*****:*****.
```

```
SGD_Scer_UTP4/YDR324C      TCTAAAGGTGCAGATTTTCTAGTTTCAGGAGGTGTTGAAAAAACACTAGTCATCAACTCA      1080
MIT_Sbay_c442_4963         ------------------------------------------------------------      924
6033_UTP4                  TCTAAAGGTGCAGATTTTCTAGTTTCAGGAGGTGTTGAAAAAACACTAGTCATCAACTCA      707


SGD_Scer_UTP4/YDR324C      CTTACTTCTTTTTCTAATGGAAACTACAGGAAGATGCCAACTGTCGAACCTTATTCAAAG      1140
MIT_Sbay_c442_4963         ------------------------------------------------------------      924
6033_UTP4                  CTTACTTCTTTTTCTAATGGAAACTaCAGGAAGATGCCAACTGTCGAACCTTATTCAAAG      767


SGD_Scer_UTP4/YDR324C      AATGTTTTAGTTAACAAAGAGCAACGCCTTGTTGTTTCATGGAGCGAATCTACTGTTAAG      1200
MIT_Sbay_c442_4963         ------------------------------------------------------------      924
6033_UTP4                  AATGTTTTAGTTAACAAAGAGCAACGCCTTGTTGTTTCATGGAGCGAATCTACTGTTAAG      827


SGD_Scer_UTP4/YDR324C      ATATGGACAATGGGAACCGATTCTAGTACAGAACAGAATTATAAGCTAGTTTGCAAGTTA      1260
MIT_Sbay_c442_4963         ------------------------------------------------------------      924
6033_UTP4                  ATATGGACAATGGGAACCGATTCtAGtACAGA----------------------------      859
```

KEM1(XRN1)/YGL173c, *Se> Sc* at 462-477bp

```
SGD_Scer_XRN1/YGL173C      -----------------------------------------------ATGGGTATTCCAA      13
FM1318/1-4548              -----------------------------------------------ATGGGTATTCCAA      13
6033_YGL173C               AAAAAGCAGCAACTGTAAAAACAGTAACCAACATATACTAGTACAGCATGGGTATTCCGA      120
                                                                          ***********.*

SGD_Scer_XRN1/YGL173C      AATTTTTCAGGTACATCTCAGAAAGATGGCCCATGATTTTACAGCTTATTGAGGGAACAC      73
FM1318/1-4548              AATTTTTCAGGTACATCTCAGAAAGATGGCCCATGATTCTACAACTTATTGAAGGGACTC      73
6033_YGL173C               AATTTTTCAGGTACATCTCAGAAAGATGGCCCATGATTCTACAACTTATTGAAGGGACTC      180
                           ************************************** ****.********.**.**:*

SGD_Scer_XRN1/YGL173C      AGATTCCTGAGTTTGATAACTTATACCTGGATATGAATTCGATTTTACATAATTGTACGC      133
FM1318/1-4548              AGATTCCCGAGTTTGATAACCTATATCTGGATATGAATTCGATTTTACATAACTGTACAC      133
6033_YGL173C               AGATTCCTGAGTTTGATAACCTATATCTGGATATGAATTCGATTTTACATAACTGTACAC      240
                           ******* ************ **** ************************** *****.*

SGD_Scer_XRN1/YGL173C      ATGGTAACGACGATGATGTAACCAAGCGATTAACTGAAGAAGAGGTTTTTGCAAAAATCT      193
FM1318/1-4548              ATGGTAACGACGATGACGTGACGAAGCGATTAACTGAAGAAGAGGTTTTTGCAAAGATCT      193
6033_YGL173C               ATGGTAACGACGATGACGTGACGAAGCGATTAACTGAAGAAGAGGTTTTTGCAAAGATCT      300
                           **************** **.** ********************************.****

SGD_Scer_XRN1/YGL173C      GTACGTATATCGATCACCTTTTTCAAACAATCAAGCCCAAGAAGATTTTCTACATGGCTA      253
FM1318/1-4548              GCACGTATATTGATCATCTTTTTCAAACTATCAAGCCCAAGCAAATTTTTTACATGGCTA      253
6033_YGL173C               GCACGTATATTGATCATCTTTTTCAAACTATCAAGCCCAAGCAAATTTTTTACATGGCTA      360
                           * ******** ***** ***********:************.*.***** **********

SGD_Scer_XRN1/YGL173C      TTGATGGTGTGGCCCCTCGTGCCAAGATGAATCAACAAAGAGCTCGTAGATTCAGAACCG      313
FM1318/1-4548              TCGATGGTGTGGCCCCCCGTGCGAAGATGAATCAACAGAGAGCTCGTAGATTTAGAACTG      313
6033_YGL173C               TCGATGGTGTGGCCCCCCGCGCGAAGATGAATCAACAGAGAGCTCGTAGATTTAGAACTG      420
                           * ************** ** ** **************.************** ***** *

SGD_Scer_XRN1/YGL173C      CTATGGATGCAGAAAAAGCCTTGAAGAAGGCTATTGAGAATGGTGACGAGATTCCTAAGG      373
FM1318/1-4548              CCATGGACGCCGAAAAAGCTATGAAAAAAGCTATTGAAAATGGTGACGAAATTCCTAAAG      373
6033_YGL173C               CCATGGACGCCGAAAAAGCTATGAAAAAAGCTATTGAAAATGGTGACGAAATTCCTAAAG      480
                           * ***** **.******** :****.**.********.***********.********.*

SGD_Scer_XRN1/YGL173C      GTGAGCCATTTGATTCGAATTCTATTACTCCAGGTACGGAGTTTATGGCCAAATTGACCA      433
FM1318/1-4548              GTGAGCCGTTCGACTCAAACTGTATTACTCCAGGTACTGAATTTATGGCTAAACTGACAA      433
6033_YGL173C               GTGAGCCGTTCGACTCAAACTGTATTACTCCAGGTACTGAATTTATGGCTAAACTGACAA      540
                           *******.** ** **.** * *************** **.******** *** ****.*

SGD_Scer_XRN1/YGL173C      AAAACTTACAATATTTTATTCACGACAAGATTTCTAACGATTCCAAATGGAGGGAAGTGC      493
FM1318/1-4548              AAAATTTACAGTATTTCATTCATGACAAAATTTCTAACGATTCAAAGTGGAGAGAAGTGC      493
6033_YGL173C               AAAATTTACAGTATTTCATTCATGACAAAATTTCTAACGATTCCAAATGGAGGGAAGTGC      600
                           **** *****.***** ***** *****.**************.**.*****.*******

SGD_Scer_XRN1/YGL173C      AAATCATATTTTCTGGCCATGAAGTTCCAGGTGAAGGTGAACACAAGATCATGAACTTTA      553
FM1318/1-4548              AAATCATATTTTCTGGCCATGAAGTTCCAGGTGAAGGTGAGCACAAAATTATGAATTTCA      553
6033_YGL173C               AAATCATATTTTCTGGCCATGAAGTTCCAGGTGAAGGTGAACACAAGATCATGAACTTTA      660
                           ****************************************.*****.** ***** ** *
```

```
SGD_Scer_XRN1/YGL173C       TAAGGCATTTAAAATCCCAAAAGGATTTCAACCAGAATACGAGACATTGTATTTACGGTC      613
FM1318/1-4548               TAAGGCATTTAAAATCCCAGAAGGATTTCAACCAAAATACGAGACACTGTATTTATGGTC      613
6033_YGL173C                TAAGGCATTTAAAATCCCAAAAGGATTTCAA-----------------------------      691
                            *******************.***********
```

PRP8/YHR165c, *Se*> *Sc* at 3222-3231bp

```
SGD_Scer_PRP8/YHR165C       TCACCAATACCATTTCCACCTTTAACTTACAAAAATGATACTAAGATATTAGTTCTTGCC      2745
FM1318_YHR165C              TCGCCAATCCCATTTCCACCTCTAACTTACAAAAATGATACCAAAATTTTGGTTCTTGCA      2754
6033_YHR165C                -----------------------AACTTAcAAAAATGATACCAAAATnTTGGTtntTGCA      37
                                                   ****** *********** **.** **.**   ***.

SGD_Scer_PRP8/YHR165C       CTCGAAGACCTAAAAGATGTCTACGCATCAAAAGTACGTTTAAATGCATCTGAAAGGGAA      2805
FM1318_YHR165C              TTAGAAAATCTAAAGGACGTTTATGCATCAAAAGTACGTTTAAATGCATCTGAAAGGGAA      2814
6033_YHR165C                TTAGAAAAtctAAAGGACGTTTATGCATCAAAAGTACGTTTAAATGCAtcTGAAAGGGAA      97
                             *.***.*   ***.** ** ** ************************  **********

SGD_Scer_PRP8/YHR165C       GAACTTGCGTTGATAGAGGAGGCTTATGATAATCCTCACGATACTTTGAACAGAATCAAA      2865
FM1318_YHR165C              GAACTGGCGTTAATAGAGGAAGCTTATGATAACCCCCACGATACTTTGAATAGAATAAAA      2874
6033_YHR165C                GAACTGGCGTTAATAGAGGAAGCTTATGATAACCCCCACGATACTTTGAATAGAATAAAA      157
                            ***** *****.********.*********** ** ************** *****.***

SGD_Scer_PRP8/YHR165C       AAGTACTTGTTGACCCAGCGAGTTTTTAAGCCTGTTGATATAACCATGATGGAAAACTAT      2925
FM1318_YHR165C              AAATACTTGTTGACGCAACGTGTTTTTAAGCCTGTTGATTTGACTATGATGGAAAACTAT      2934
6033_YHR165C                AAATACTTGTTGACGCAACGTGTTTTTAAGCCTGTTGATTTGACTATGATGGAAAACTAT      217
                            **.*********** **.**:******************:*.** ***************

SGD_Scer_PRP8/YHR165C       CAAAACATTTCCCCTGTTTATTCAGTTGATCCCTTGGAAAAGATTACCGATGCATATCTT      2985
FM1318_YHR165C              CAAAATATATCTCCTGTTTATGCAGTCGATCCCTTGGAAAAAATCACCGATGCATATCTT      2994
6033_YHR165C                CAAAATATAtctCCTGTTTATGCAGTCGATCCCTTGGAAAAAATCACCGATGCATATCTT      277
                            ***** **:   ********* **** **************.** ***************

SGD_Scer_PRP8/YHR165C       GATCAGTATTTATGGTACGAAGCTGACCAACGAAAGCTTTTCCCAAACTGGATTAAACCA      3045
FM1318_YHR165C              GATCAGTATTTGTGGTACGAAGCCGATCAAAGGAAGCTTTTCCCCAACTGGATAAAACCA      3054
6033_YHR165C                GATCAGTATTTGTGGTACGAAGCCGATCAAAGGAAGCTTTTCCCCAACTGGATAAAACCA      337
                            ***********.*********** ** ***.*.***********.********:******

SGD_Scer_PRP8/YHR165C       AGTGATTCAGAGATACCACCTCTTCTGGTATACAAGTGGACTCAGGGTATAAACAACCTA      3105
FM1318_YHR165C              AGTGATTCAGAGATACCACCTCTTCTGGTATACAAGTGGTCTCAAGGCATAAACAATCTA      3114
6033_YHR165C                AGTGATTCAGAGATACCACCTCTTCTGGTATACAAGTGGTCTCAAGGCATAAACAATCTA      397
                            ***************************************:****.** ******** ***

SGD_Scer_PRP8/YHR165C       TCTGAAATTTGGGATGTATCCAGAGGCCAATCTGCAGTTTTGCTTGAGACTACTTTGGGT      3165
FM1318_YHR165C              TCTGACATTTGGGATGTCTCTAAAGGTCAATCCACAGTTTTGCTCGAGACCACGTTAGGG      3174
6033_YHR165C                TCTGACATTTGGGATGTCTCTAAAGGTCAATCCACAGTTTTGCTCGAGACCACGTTAAGG      457
                            *****.***********.** *.*** ***** .********** ***** ** **..*

SGD_Scer_PRP8/YHR165C       GAAATGGCCGAAAAAAATTGACTTTACTTTACTTAATAGATTACTTCGCCTGATTGTAGAT      3225
FM1318_YHR165C              GAAATGGCTGAAAAGATTGATTTTACTTTGTTAAATAGATTACTTCGTCTGATTGTGGAC      3234
6033_YHR165C                GAAATGGCTGAAAAGATTGATTTTACTTTGTTAAATAGATTACTTCGTCTGATTGTAGAT      517
                            ******** *****.***** ********. *:************** ********.**

SGD_Scer_PRP8/YHR165C       CCTAATATTGCTGACTATATCACTGCAAAAAATAATGTTGTTATCAACTTTAAAGATATG      3285
FM1318_YHR165C              CCCAATATTGCTGACTACATTACTGCAAAAAACAATGTTATGATCAACTTTAAGGATATG      3294
6033_YHR165C                CCTAATATTGCTGACTATATTACTGCAAAAAATAATGTTGTTATCAACTTTAAAGATATG      577
                            ** ************** ** *********** ******.* ***********.******

SGD_Scer_PRP8/YHR165C       AGTCACGTCAACAAATATGGCTTAATACGCGGGTTGAAGTTCGCTTCTTTCATATTCCAA      3345
FM1318_YHR165C              AGTCACGTTAACAAATACGGTCTAGTACGCGGATTACAGTTTGCCTCTTTTATATTCCAA      3354
6033_YHR165C                AGTCACGTCAACAAATATGGCTTAATACGCGGGTTGAAGTTCGCTTCTTTCATATTCCAA      637
                            ******** ******** **  **.*******.**..**** ** ***** *********

SGD_Scer_PRP8/YHR165C       TATTACGGACTAGTTATAGATCTTTTACTATTGGGTCAGGAAAGGGCTACAGATTTGGCT      3405
FM1318_YHR165C              TATTATGGACTAGTTGTTGATCTTTTGTTGTTGGGCCAAGAAAGGGCTGCTGATTTGGCT      3414
6033_YHR165C                TATTACGGACTAGTTATAGATCTTTTACTATTGGGTCAGGAAAGGGCTACAGATTTGGCT      697
                            ***** *********.*:********. *.***** **.*********.*:*********
```

```
SGD_Scer_PRP8/YHR165C       GGTCCAGCTAACAATCCAAATGAATTTATGCAATTCAAGAGCAAAGAAGTAGAAAAGGCA      3465
FM1318_YHR165C              GGCCCTGCCAACAATCCAAATGATTTTATGCAATTCAAAAGCAGAGAAACAGAAAAGACA      3474
6033_YHR165C                GGTCCAGCTAACAATCCAAATGAATTTATGCAATTCAAGAGCAAAGAAGTAGAAAAGGCA      757
                            ** **:** **************:**************.****.****. *******.**

SGD_Scer_PRP8/YHR165C       CATCCGATCAGACTTTACACCAGATATTTAGATCGTATATATATGCTTTTTCACTTTGAA      3525
FM1318_YHR165C              CATCCGATCAGGCTCTATACCAGATATTTGGATCGCATTTATATGGTCTTTCGCTTTGAA      3534
6033_YHR165C                CATCCGATCAGACTTTACACCAGATATTTAGATCGTATATATATGCTTTTTCACTTTGAA      817
                            ***********.** ** ***********.***** **:****** * ****.*******

SGD_Scer_PRP8/YHR165C       GAAGATGAGGGAGAGGAATTGACTGATGAATACTTGGCAGAGAATCCAGATCCAAACTTT      3585
FM1318_YHR165C              GAAAGTGAGGGAAATGAACTGACAGATGAATATTTAGCGGAAAACCCAGATCCAAACTTT      3594
6033_YHR165C                GAAGATGAGGGAGAGGAATTGACTGATGAATACTTGGCAGAGAATCCAGATCCAAACTTT      877
                            ***..*******.* *** ****:******** **.**.**.** ***************

SGD_Scer_PRP8/YHR165C       GAAAACAGCATCGGATACAATAATAGAAAATGCTGGCCCAAAGATTCGCGCATGAGGCTA      3645
FM1318_YHR165C              GAAAATAGCATTGGATACAATAACAGAAAATGCTGGCCTAAGGACTCACGCATGAGGCTA      3654
6033_YHR165C                GAAAACAGCATCGGATacAATAAtagAAAATGTTGGCCCAAAGATTCGCGCATGAGGCTA      937
                            ***** ***** ****  *****   ****** ***** **.** **.************

SGD_Scer_PRP8/YHR165C       ATACGTCAGGACGTTAACCTAGGGCGAGCAGTGTTTTGGGAAATTCAAAGTAGGGTGCCA      3705
FM1318_YHR165C              ATCCGCCAGGATGTAAACTTGGGACGTGCAGTGTTTTGGGAAGTTCAAGGCAGAGTGCCT      3714
6033_YHR165C                ATACGtcAGGACGTA---------------------------------------------      952
                            **.**  **** **:
```

Intergenic (chrVIII:451249-451261)

```
SGD_Scer_ENO2/YHR174W       TTTGCCATATGCAAAATTC------ATGAAGTGTGATACCAAGTCAGCATACACCTCACT      662
MIT_SBAY_C173_10953         CTTTTCAAGTGCAAAGCTCACGAAATTTCGAATTGGTACCAGGTCAGCATACACACTACT      663
6033_chrVIII_int            --------------------------------TTGGTACCAGGTCAGCATACACACTACT      28
                                                             **.*****.************.  ***

SGD_Scer_ENO2/YHR174W       AGGGTAGTTTCTTTGGTTGTATTGATCATTTGGTTCATCGTGGTTCATTAAT---TTTTT      719
MIT_SBAY_C173_10953         AGGGTAGTTTCTTTGGCTGTATTGATCATTTGATTCAACGTGGTACAGCATTTTTTTTTT      723
6033_chrVIII_int            AGGGTAGTTTCTTTGGCTGTATTGATCNTTTGGTTCAACGTGGTCCTACATT---TTTTT      85
                            **************** ********** ****.****:****** *:  *:*   *****

SGD_Scer_ENO2/YHR174W       TTCTCCATTGCTTTCTGGCTTTGATCTTACTATCATTTGGATTTTTGTCGAAGGTTGTAG      779
MIT_SBAY_C173_10953         TTTTCCATTGCATTCTGGCTTTGAACTTATAATCATTTGGAGCTCTGTCGAAGGTCGTGG      783
6033_chrVIII_int            TTTTCCATTGCATTCGGGCTTTCAACTTATTGTCTTTTGGATCTCTGTCGAAGGTCGGGG      145
                            ** ********:*** ****** *:**** :.**:******  * ********** * .*

SGD_Scer_ENO2/YHR174W       AATTGTATGTGACAAGTGGCACCAAGCATATATAAAAAAAAAAAGCATTATCTTCCTACC      839
MIT_SBAY_C173_10953         AACTGTATTGTGAGAAATTGGCACGAACATATAT-AACGAAATGCATTATCTTTTTTTTT      842
6033_chrVIII_int            AAGTGTATTGTGAGGAATTGG--CACGAATATTTATAACGAAATGCTTTATCTTTTTTTT      203
                            ** *****   .....:   .  ..  .:::::: :*. .**:  .:*::  **  *:

SGD_Scer_ENO2/YHR174W       AGAGTTGATTGTTAAAAACGTATTTATAGCAAACGCAATTGTAATTAATTCTTATTTTGT      899
MIT_SBAY_C173_10953         TAGGTTGATTGTTAGAAACGTATTTATAGCTAATGCAATTGTAATTAATTCTCATTTGT-      901
6033_chrVIII_int            TAGGTTGATTGTTAAAAACGTTTTTATAGCTAAGGCAATTGTAATTAATTCTCATTTGT-      262
                            :..***********.******:********:** ****************** ****

SGD_Scer_ENO2/YHR174W       ATCTTTTCTTCCCTTGTCTCAATCTTTTATTTTTATTTTATTTTTCTTTTCTTAGTTTCT      959
MIT_SBAY_C173_10953         ATCTTCTCTTCTTTTGTTTCCTTATCTTATATT--CTTTATTTAATTTTATTCTATTCCT      959
6033_chrVIII_int            ATCTTCCCTTCTTTTATTTTCTTATATTTTCTTATTTATTTTTCTTTTATCTTATTTTCT      322
                            *****  ****  **.* * .:*.* **:* **   *:*:*** : **:: * : ** **

SGD_Scer_ENO2/YHR174W       TTCATAACACCAAGCAACTAATACTATAACATACAATAATAATGGCTGTCTCTAAAGTTT      1019
MIT_SBAY_C173_10953         TTCATATCTCCAAGCAACTAATACTATATCAAACAATAATAATGGCTGTCTCTAAAGTTT      1019
6033_chrVIII_int            TTCATAACCCCAAGCAACTAATACTATAACATACAATAATAAGGGCTGTCTCTAAAGTTT      382
                            ******:* *******************:**:********** *****************

SGD_Scer_ENO2/YHR174W       ACGCTAGATCCGTCTACGACTCCCGTGGTAACCCAACCGTCGAAGTCGAATTAACCACCG      1079
MIT_SBAY_C173_10953         ACGCTAGATCCGTCTACGACTCCCGTGGTAACCCAACCGTCGAAGTCGAATTAACCACCG      1079
6033_chrVIII_int            ACCCTAAATCCGTCTACNACTCCCGGGGNAACCCAACCGTCAAANTCAAATTANCCCCCG      442
                            ** ***.********** ******* **.************.** **.***** **.***

SGD_Scer_ENO2/YHR174W       AAAAGGGTGTTTTCAGATCCATTGTTCCATCTGGTGCCTCCACCGGTGTCCACGAAGCTT      1139
MIT_SBAY_C173_10953         AAAACGGTGTTTTCAGATCCATGGTCCCATCTGGTGCCTCCACTGGTATCCACGAAGCCT      1139
6033_chrVIII_int            AAAAGGGGGTTTTCAAATCCNTTGTTCCNTCNGGNGCCTCCACCGGTGTCCACAAANCTT      502
                            **** ** *******.**** * ** ** **.**.******** ***.*****.** * *
```

```
SGD_Scer_ENO2/YHR174W       TGGAAATGAGAGATGAAGACAAATCCAAGTGGATGGGTAAGGGTGTTATGAACGCTGTCA          1199
MIT_SBAY_C173_10953         TGGAAATGAGAGATGGTGACAAATCTAAATGGTTAGGTCAAGGTGTCTTGAACGCTGTTA          1199
6033_chrVIII_int            TGGAAATGAAANATGAAAACAAATCCAAGTGNATGGGTAAGGGTGTTATGAACGCTGTCA          562
                            *********.* ***.:.******* **.** :*.***.*.***** :********** *

SGD_Scer_ENO2/YHR174W       ACAACGTCAACAACGTCATTGCTGCTGCTTTCGTCAAGGCCAACCTAGATGTTAAGGACC          1259
MIT_SBAY_C173_10953         AGAACGTCAACGATGTTGTCGCTCCAGCTTTCGTTAAGGCTAACCTAGATGTCAAGAACC          1259
6033_chrVIII_int            ACAACGTCAACAACGTCTTTGCTGCTGCTTTCGTCAAGGCCAACCTANATGTTAAGGACC          622
                            * *********.* **  * *** *:******** ***** ****** **** ***.***

SGD_Scer_ENO2/YHR174W       AAAAGGCCGTCGATGACTTCTTGTTGTCTTTGGATGGTACCGCCAACAAGTCCAAGTTGG          1319
MIT_SBAY_C173_10953         AATCCGCTGTCGATGACTTCTTGTTGTCTTTGGACGGTACCGCTAACAAGTCCAAGTTGG          1319
6033_chrVIII_int            AAAAGGCCGTCAATGACTTCTTGTTGTCTTTGGATGGTACCGCCAACAAGTCCAAGTTGG          682
                            **:. ** ***.********************** ******** ****************

SGD_Scer_ENO2/YHR174W       GTGCTAACG-CTA-TCTTGGGTGTCTCCATGGCCGCTGCTAGAGCCGCTGCTGCTGAA-A          1376
MIT_SBAY_C173_10953         GTGCTAACGCTA--TCTTGGGTGTTTCCATGGCCGCTGCTAGAGCCGCTGCCGCTGAA-A          1376
6033_chrVIII_int            GGTGCTAACGCTATCTTGGGGTGTCTCCATGGCCGCTGCTAGAGCCGCTGCTGCTNAAAA          742
                            *    :*.   :    * ****** ************************** *** ** *

SGD_Scer_ENO2/YHR174W       AGAACGTCCCATTGTACCAACATTTGGCTGACTTGTCTAAGTCCAAGACCTCTC-CATAC          1435
MIT_SBAY_C173_10953         AGAACGTCCCATTGTACCAACATTTGGCTGACTTGTCCAAGTCTAAGACTTCTC-CATTC          1435
6033_chrVIII_int            AGAACGTCCCATTGTACCAACNTTTGGCTGACTTGTCTAAGTCCAAGACCTNCTCCATAC          802
                            ********************* *************** ***** ***** *    ***:*

SGD_Scer_ENO2/YHR174W       GTTTTGCCAGTTCCATTCTTGAACGTTTTGA---ACGGTGGTTCCCACGCTGGTGGTGCT          1492
MIT_SBAY_C173_10953         GTTTTGCCAGTTCCATTCTTGAACGTTTTG---AACGGTGGTTCCCACGCTGGTGGTGCT          1492
6033_chrVIII_int            GTTTTGCCAGTTCCATTCTTGGAACGTTTTNNAACGGGNGGTNCCCACGCTGGTGGNNGN          862
                            *********************.*.  ***     . **.***.*************.  .

SGD_Scer_ENO2/YHR174W       TTGGCTTTGCAAGAATTCATGATTGCTCCAACTGGTGCTAAGACCTTCGCTGAAGCCATG          1552
MIT_SBAY_C173_10953         TTGGCTTTGCAAGAATTCATGATTGCTCCAACTGGTGCCAAGACCTTCGCCGAAGCTTTG          1552
6033_chrVIII_int            TTNGNCTTNCANGAANTCATGATTGCTCNNCTGGGT------------------------          898
                            ** *  ** ** ***.************  .  ***
```

MSU1(DSS1)/YMR287c, *Se>Sc* at 1710-1723bp

```
SGD_Scer_DSS1/YMR287C       GAAATCCATCTGAATACAGCGTTGCTGTCGCCTATATCAGTAACGATTATTCCCTTAAAA          1140
FM1318                      AAAATACACTTGAATANNNNNNNNNNNNNNNNNNNNNNNNNNNTACAATCATCCCTTTGAAA          1140
6033_YMR287C                -------------------------------ctACATCAGTTACAATCATCCCTTTGAAA          29
                                                                .   .:**.** ** ** **.***

SGD_Scer_DSS1/YMR287C       TCACAACATTTATATTATGCGCAGGTGATAGAGAAGCTAGAAGCCAACAGTTATAGAGAG          1200
FM1318                      TCCCAACACTTATACTATGCACAGGTAATAGAGAAGCTAGAAGCTGACAACTACAAGGAC          1200
6033_YMR287C                TCCCAACACTTATACTACGCACAGGTAATAGAGAAGCTAGAAGCTGACAACTACAAGGAC          89
                            **.***** ***** ** **.*****.***************** .***. ** *..**

SGD_Scer_DSS1/YMR287C       GTCAATAAGTTTGTAAAATTGGTGAATGAAAGGAAATATCGCGATATATCAGCTTTATAT          1260
FM1318                      ATCGACCGATTTGTAAAGTTGGTGAACGAAAGAAAATATAAAGATATCTCATCTTTATAC          1260
6033_YMR287C                ATCGACCGATTTGTAAAGTTGGTGAACGAAAGAAAATATAAAGATATCTCATCTTTATAC          149
                            .**.* ...********.******** *****.******...*****.*** *******

SGD_Scer_DSS1/YMR287C       CCTTCTGTGATCCAATTATTGAAAGATTTTGCTGCTGGGAATTTTCACAATAACGGAATT          1320
FM1318                      CCTTCTGTCATTCAGCTGCTGAAAGATTTTGCCGCGGGAAACTTCCACAATAATGGAGTT          1320
6033_YMR287C                CCTTCTGTCATTCAGCTGCTGAAAGATTTTGCCGCGGGAAACTTCCACAATAATGGAGTT          209
                            ******** ** **. *. ************* ** **.** ** ******** ***.**

SGD_Scer_DSS1/YMR287C       ATAGTAGCTTTGATCTCAAAAATATTCAGAAAGATAGAACGCTATAAGGATTGTGACATC          1380
FM1318                      ATAGTCACCTTAATCTCCAAAATATTTAGAAAGATAGAACTCTATAAGAGTTTTGATATA          1380
6033_YMR287C                ATAGTCACCTTAATCTCCAAAATATTTAGAAAGATAGAACTCTATAAGAGTTTTGATATA          269
                            *****..* **.*****.******** ************* *******..** *** **.

SGD_Scer_DSS1/YMR287C       ACAAGAGATATATGTCAAGATTTAATCAATGAGATAACACCCAACTCAATACCCAATCCA          1440
FM1318                      ACAAGAGATATATGTCAAGATCTGGTCAATGAAATTTTACCCAATAAAATGATCAATCCG          1440
6033_YMR287C                ACAAGAGATATATGTCAAGATCTGGTCAATGAAATTTTACCCAATAAAATGATCAATCCG          329
                            ********************* *..*******.**:: ****** :.***.. ******.

SGD_Scer_DSS1/YMR287C       TTGTTACTTAACATGGATTTAGCATTACCTGCTTCCTCGAAGTTAGTGAAATGGCAACAA          1500
FM1318                      TTGTTACTGAATATGGATTTAGCTTTGCCTGCATCTTCGAAATTGGGGCAATCACAGCAA          1500
```

```
6033_YMR287C                 TTGTTACTGAATATGGATTTAGCTTTGCCTGCATCTTCGAAATTGGGGCAATCACAGCAA      389
                             ******** ** ***********:**.*****:** *****.**.* *.*** .**.***

SGD_Scer_DSS1/YMR287C        AAACTCTACGACTTAACTAATATAGAAGAATTGCAATGGAAAAAATCCGGCACTGATGAC      1560
FM1318                       AAGCTTTATGAGTTGACAGACANNNNNNNNNNNNNNNNNNNNNNNCCAATATTAATGGT      1560
6033_YMR287C                 AAGCTTTATGAGTTGACAGACATAGAAAACCTGCAAGCAAGTAATTCCAATATTAATGGT      449
                             **.** ** ** **.**:.* *.         .              .**.. * *.***.

SGD_Scer_DSS1/YMR287C        GATAGGTACGATTTTGGCGATCTTAGGGTTTTTTGTATAGACTCTGAAACTGCGCATGAG      1620
FM1318                       GATAGATATGATTTTGGTGATCTTAAGGTTTTTTGTATAGATTCGGAGACTGCGCATGAG      1620
6033_YMR287C                 GATAGATATGATTTTGGTGATCTTAAGGTTTTTTGTATAGATTCGGAGACTGCGCATGAG      509
                             *****.** ******** *******.*************** ** **.************

SGD_Scer_DSS1/YMR287C        ATTGATGACGGCGTGTCGGTGAAAAACTATGGTAGAGATGGACTGTACACTTTATATATT      1680
FM1318                       ATTGACGATGGAATCTCGATAGAAAACCACAGAAAGGACGGATTGTACACTTTACATATC      1680
6033_YMR287C                 ATTGACGATGGAATCTCGATAGAAAACCACAAAAAGGACGGATTGTACACTTTACATATC      569
                             ***** ** **..* ***.*..***** * ..:*..** *** *********** ****

SGD_Scer_DSS1/YMR287C        CATATTGCGGATCCAACTTCTATGTTTCCGGAAAGCACTAATGTTGATATTGAGGGTATA      1740
FM1318                       CATATTGCAGATCCTACTTCTTTGTTTCCAGAAAGCACTAATCTTGATTCTCAGGGTATT      1740
6033_YMR287C                 CATATTGCAGATCCTACTTCTTTGTTTCCAGAAAGCACTAATGTTGATATTGAGGGTATA      629
                             ********.*****:******:*******.************ *****: * *******:

SGD_Scer_DSS1/YMR287C        AGTACAGATATCCTAAATGTTGCCTTGAAAAGATCATTTACCACATATTTACCAGATACG      1800
FM1318                       ACCACGGATGTTCTAAATGTTGCATTAAAAAGGTCGTTCACCACATATTTGCCTGATTTG      1800
6033_YMR287C                 AGTACAGATATCCTAAATGTTGCCTTGAAAAGATCATTTACCACATATTTACCAGATACG      689
                             *  **.***.* ***********.**.*****.**.** ***********.**:***: *

SGD_Scer_DSS1/YMR287C        GTTGTTCCTATGTTACCTCAATCTATTTGTCACTTATCAGATTTGGGGAAACAAGGACAA      1860
FM1318                       GTTGTTCCCATGCTACNNNNNNNNNNNNNNNNNNNNNNNNNNNNTGGGAAAGCAAGGGCAA      1860
6033_YMR287C                 GTTGTTCCTATGTTACCTCAATCTATTTGTCACTTATCAGATTTGGGGAAACAAGGACAA      749
                             ******** *** *** .   . . ... .   .. .    ..****.**.*****.***

SGD_Scer_DSS1/YMR287C        AGGACGAAAACTATATCCTTCTCTGTTGATGTTAAAATCACTTCTAAATGCAGTGGAAAA      1920
FM1318                       AGGACAAGAACAATATCGTTTTCTGTTGACGTTAAAATTCTCCCTAAAAGCACTGGAGAA      1920
6033_YMR287C                 AGGACGAAAACTATATCCTTCTCTGTTGATGTTAAAATCACTTCTAAATGCAGTGGAAAA      809
                             *****.*.***:***** ** ******** ******** .   *****:*** ****.**

SGD_Scer_DSS1/YMR287C        TCAATTGAAATTATGTATGACAGTTTTAAAATCCGGAAAGGCATTGTGTCCAACTTCCCA      1980
FM1318                       CCCATTGAAATTATGTATGATAGCCTTAAAATTCGGAAAGGTATTGTGTCGAATTTCCCT      1980
6033_YMR287C                 TCAATTGAAATTATGTATGACAGTTtaaAATCCGGAAAGGCAnT----------------      853
                              *.***************** **     **:   *.**.*   *
```

HSP82/YPL240c, Se> *Sc* at 1578-1608bp*

```
SGD_Scer_HSP82/YPL240C       GAAGACCCATTGTACGTTAAGCATTTCTCCGTTGAAGGTCAATTGGAATTTAGAGCTATC      960
MIT_Sbay_c60_24336           ------------------------------------------------------------      0
6033_YPL240C                 ----------------------------------AAGGTCAGTtAGAATTCAGAGCCAtc      26

SGD_Scer_HSP82/YPL240C       TTATTCATTCCAAAGAGAGCACCATTCGACTTGTTTGAGAGTAAAAAGAAGAAGAATAAT      1020
MIT_Sbay_c60_24336           ------------------------------------------------------------      0
6033_YPL240C                 tTGTACATtCCAAAGAGAnntCCATTTGantTATTcGAAAGTAAGAAGAagaagaAcAAc      86

SGD_Scer_HSP82/YPL240C       ATCAAGTTGTACGTTCGTCGTGTTTTCATCACTGATGAAGCTGAAGACTTGATTCCAGAG      1080
MIT_Sbay_c60_24336           ------------------------------------------------------------      0
6033_YPL240C                 ATCAAGTTGTATGTTCGTCGTGTTtTcntCACCGACGAAGCTGAAGACTTGATCCCAGAA      146

SGD_Scer_HSP82/YPL240C       TGGTTATCTTTCGTCAAGGGTGTTGTTGACTCTGAGGATTTACCATTGAATTTGTCCAGA      1140
MIT_Sbay_c60_24336           ---ATGTCATTCGTCAAGGGTGTTGTTGACTCCGAAGATTTACCATTGAACTTGTCCAGA      57
6033_YPL240C                 TGGATGTCCTTTGTCAAGGGTGTTGTTGACTCTGAAGACTTACCAtTGAACCTGTCCAGA      206
                                :*.** ** ******************* **.** ****** ****  ********
```

```
SGD_Scer_HSP82/YPL240C      GAAATGTTACAACAAAATAAGATCATGAAGGTTATTAGAAAGAACATTGTCAAAAAGTTG      1200
MIT_Sbay_c60_24336          GAAATGTTGCAACAAAACAAGATCATGAAGGTCATCAGAAAGAACATCGTTAAGAAGATG      117
6033_YPL240C                GAAATGCTACAACAAAACAAGATCATGAAGGTTATCAGAAAGAACATCGTCAAGAAGGTA      266
                            ****** *.******** ************** ** *********** ** **.*** *.

SGD_Scer_HSP82/YPL240C      ATTGAAGCCTTCAACGAAATTGCTGAAGACTCTGAACAATTTGAAAAGTTCTACTCGGCT      1260
MIT_Sbay_c60_24336          ATTGAATCCTTCAACGAAATCGCTGAAGACTCTGAACAATTCGAAAAGTTCTACTCTGCC      177
6033_YPL240C                ATTGAAGCTTTCAACGAAATTGCTGAAGACTCTGAACAATTCGAAAAGTTCTACTCTGCC      326
                            ****** * *********** ******************** ************** **

SGD_Scer_HSP82/YPL240C      TTCTCCAAAAATATCAAGTTGGGTGTACATGAAGATACCCAAAACAGGGCTGCTTTGGCT      1320
MIT_Sbay_c60_24336          TTCGCTAAGAACATCAAATTGGGTGTTCATGAAGACACTCAAAACAGAGCTGCCTTGGCC      237
6033_YPL240C                TTCGCTAAGAACATCAAATTGGGTGTCCACGAAGACACTCAAAACAGAGCTGCCTTGGCT      386
                            *** * **.** *****.******** ** ***** ** ********.***** *****

SGD_Scer_HSP82/YPL240C      AAGTTGTTACGTTACAACTCTACCAAGTCCGTAGATGAGTTGACTTCCTTAACTGATTAC      1380
MIT_Sbay_c60_24336          AAATTGCTACGTTACAACTCCACCAAGTCCGTTGACGAATTGACTTCTTTGACTGATTAT      297
6033_YPL240C                AAGTTACTACGTTACAACTCCACCAAGTCCGTCGACGAATTAACTTCTTTGACCGATTAC      446
                            **.**. ************* *********** ** **.**.***** **.** *****

SGD_Scer_HSP82/YPL240C      GTTACCAGAATGCCAGAACACCAAAAGAACATCTACTACATCACTGGTGAATCTCTAAAG      1440
MIT_Sbay_c60_24336          ATTACCAGAATGCCAGAACACCAAAAGAACATCTACTATATCACAGGTGAGTCTTTGAAG      357
6033_YPL240C                ATCACCAGAATGCCAGAACACCAAAAGAACATTTACTACATCACAGGTGAATCTCTAAAG      506
                            .* ***************************** ***** *****:*****.*** *.***

SGD_Scer_HSP82/YPL240C      GCTGTCGAAAAGTCTCCATTTTTGGATGCCTTGAAGGCTAAAAACTTCGAGGTTTTGTTC      1500
MIT_Sbay_c60_24336          GCTGTTGAAAAAATCCCCATTCTTAGACGCTTTGAAAGCTAAGAACTTTGAAGTTTTGTTC      417
6033_YPL240C                GCCGTTGAAAAAATCCCCATTCTTGGACGCTTTGAAGGCTAAGAACTTTGAAGTTTTGTTC      566
                            ** ** *****.** ***** **.** ** *****.*****.***** **.*********

SGD_Scer_HSP82/YPL240C      TTGACCGACCCAATTGATGAATACGCCTTCACTCAATTGAAGGAATTCGAAGGTAAAACT      1560
MIT_Sbay_c60_24336          TTGACTGATCCAATCGATGAATACGCCTTCACTCAATTAAAGGAATTCGAAGGTAAGACT      477
6033_YPL240C                TTGACTGATCCAATCGATGAATACGCTTTCACCCAATTGAAGGAATTCGAAGGTAAGACT      626
                            ***** ** ***** *********** ***** *****.*****************.***

SGD_Scer_HSP82/YPL240C      TTGGTTGACATTACTAAAGATTTCGAATTGGAAGAAACTGACGAAGAAAAAGCTGAAAGA      1620
MIT_Sbay_c60_24336          TTAGTCGATATCACCAAGGATTTCGAGCTGGAAGAAACTGACGAAGAGAAAGCTGAAAGA      537
6033_YPL240C                TTGGTCGATATCACCAAGGATTTCGAACTGGAAGAAACTGACGAAGAAAAAGCTGAAAGA      686
                            **.** ** ** ** **.********. *******************.************

SGD_Scer_HSP82/YPL240C      GAGAAGGAGATCAAAGAATATGAACCATTGACCAAGGCCTTGAAAGAAATTTTGGGTGAC      1680
MIT_Sbay_c60_24336          GAAAAGGAAGTTAAAGAATTCGAACCATTGACCAAGGCCTTGAAAGACATCTTGGGTGAA      597
6033_YPL240C                GAGAAGGAGATCAAAGAATATGAACCATTGACCAAGGCCTTGAAAGAAATTTTGGGTGAC      746
                            **.*****..* *******: ***************************.** ********.

SGD_Scer_HSP82/YPL240C      CAAGTGGAGAAAGTTGTTGTTTCTTACAAATTGTTGGATGCCCCAGCTGCTATCAGAACT      1740
MIT_Sbay_c60_24336          CAAGTTGAAAAGGTTGTTGTCTCTTACAAACTAGTGGATGCCCCAGCTGCCATTAGAACT      657
6033_YPL240C                CAAGTGGAGAAAGTTGTTGTTTCTTACAAATTGTTGGATGCCCCAGCTGCTATCAGAACT      806
                            ***** **.**.******** ********* *. **************** ** ******

SGD_Scer_HSP82/YPL240C      GGTCAATTTGGTTGGTCTGCTAACATGGAAAGAATCATGAAGGCTCAAGCCTTGAGAGAC      1800
MIT_Sbay_c60_24336          GGCCAATTCGGTTGGTCCGCTAACATGGAAAGAATCATGAAGGCTCAAGCTTTGAGAGAC      717
6033_YPL240C                GGTCAATTTGGTTGGTCTGCTAACATGGAAAGAATCATGAAGGCTCAAGCCTTGAGAGAC      866
                            ** ***** ******** ******************************** *********

SGD_Scer_HSP82/YPL240C      TCTTCCATGTCCTCCTACATGTCTTCCAAGAAGACTTTCGAAATTTCTCCAAAATCTCCA      1860
MIT_Sbay_c60_24336          TCTTCCATGTCCTCTTACATGTCCTCCAAGAAGACTTTCGAAATCTCTCCAAAATCTCCA      777
6033_YPL240C                TCTTCCATGTCCTCCTACATGTCTTCCAAGAAGACTTTCGAAATTTCTCCAAAATCTCCA      926
                            ************** ******** ******************** ***************

SGD_Scer_HSP82/YPL240C      ATTATCAAGGAATTGAAAAAGAGAGTTGACGAAGGTGGTGCTCAAGACAAGACTGTCAAG      1920
MIT_Sbay_c60_24336          ATTATCAAGGAATTGAAAAAGAGAGTTGATGAAGGCGGTGCTCAAGATAAGACTGTCAAG      837
6033_YPL240C                ATTATCAAGGAATTGAAAAAGAGAGTTGACGAAGGTGGTGCTCAAGACAAGACTGTcAAG      986
                            ***************************** ***** *********** ********* ***

SGD_Scer_HSP82/YPL240C      GACTTGACTAAGTTATTATATGAAACTGCTTTGTTGACTTCCGGCTTCAGTTTGGACGAA      1980
MIT_Sbay_c60_24336          GATTTGACCAACTTATTATTCGAAACCGCTCTGTTAACTTCTGGTTTCAGTCTGGAAGAA      897
6033_YPL240C                GAC---------------------------------------------------------      989
                            **
```

Intergenic (chrXVI:906846-906880)

```
SGD_Scer_GDB1/YPR184W      CCCTATCTCTATTGATAATAAAACAAATAAAAATCGTATATTTTTGTATATGTATTATCG   5779
WASHU_SBAY_CONTIG654.29    CTGTTAA---------TAAAACGTTCGAATTCGTATACCCACCTGTGTCTATTACTATCG   5763
6033_chrXVI_int            --------------------------------------------TGTATATGTATTATCG   16
                                                                       :  :: ** *****

SGD_Scer_GDB1/YPR184W      TTTTGAGGAGCGAGAGCGAAAACGAAAATAAAAGAAATAAAGGGAACACACGTGAAAGTG   5839
WASHU_SBAY_CONTIG654.29    TTTTGAGGAGCGAGAGCGAAAACGAAACTAAAAGAAATAAAGGGAACACACGTGAAAGTG   5823
6033_chrXVI_int            TTTTGAGGAGCGAGAGCGAAAACGAAAATTAAAGAAATAAAGGGAACACACGTGAAAGTG   76
                           ***************************.*:******************************

SGD_Scer_GDB1/YPR184W      AAGGACAGCACCAACCAAGGGGGACAAAACGCGTCAGAGCAAGAGGTGAAAGGGTTGCCA   5899
WASHU_SBAY_CONTIG654.29    AGAAACAAAAGCTACCAAAGGGGT-AAAACACGTCGGAACGAGTGGCAAAAGCGCTGTAA   5882
6033_chrXVI_int            AGAGACAAAAGTTACCAAAGGGGC-AAGGCACGTCGGAACGAGTGGCAAAAGGGCTGTAA   135
                           *...***..*   :*****.****  **..*.****.**.*.**:**  .**** * **  .*

SGD_Scer_GDB1/YPR184W      CGTACGTATTTGAACCGGAGTGAGCCGTTTGCGTGTTACAATCAATTCGATATCTTTGTT   5959
WASHU_SBAY_CONTIG654.29    CGTACGTATTGAAAGAGGATAGCGAGTGATTTGTGTAATATAGGCAGTCAGTATCCTTGT   5942
6033_chrXVI_int            CGTACGTAATAGAAGAGGATAGAGAGCCATTTGTGTAATACAGGCAGTTAGTATCCTTAT   195
                           ********:* .** .*** :*.*.   :*  ****:* * : ..:   * ::   *  *

SGD_Scer_GDB1/YPR184W      GTTTTGTTTAATTCGCTAAGATCTAAGTTTTTAACTAAGTGAGCAGCGGCAGTTACAGAA   6019
WASHU_SBAY_CONTIG654.29    TGTTTGTTAAGTTCGCTTGGATTTAGATCTCTAGCTAAGTAAACAGCGGCAGTTTAAGAA   6002
6033_chrXVI_int            TTTTTGTTTAGTTCGCTTGGATTTAGATCTTTAGCCAAGTAAACAGCGGCAGGTAAAGAT   255
                             ******:*.******:.*** **..* * **.* ****.*.********* *:.***:

SGD_Scer_GDB1/YPR184W      AGAGGCGAGAAGTAGAAACAAGGGTGTATAGATTTTCGTTACCTCACATAGATATAGAAC   6079
WASHU_SBAY_CONTIG654.29    AGAAAAAAAAAAGCACA-ACAAAGGTGTGAAGATTCCCATTCTGTCACATAGTTATAGAGC   6061
6033_chrXVI_int            AGAGAAAAAAAAGCACA-ACAAAGGTGTGGAGATTCCCATTCTGTCACATAGTTATAGAGC   314
                           ***....*.*** * * ****.*****. *****  *.**.  ********:******.*

SGD_Scer_GDB1/YPR184W      GAATTCATAGGTACCAAATACATTCC-ACTTACA-CCTTCCAGGCTCAAGTCTTGAAAAG   6137
WASHU_SBAY_CONTIG654.29    CAACTTTATAGAAATAAAGTGCATCTCCTCTACGTTTTTGAAAGTTCGGGTGTTGGACAA   6121
6033_chrXVI_int            CAACTTTACGGGAATAAAGTGCATCCCATTTACGCTTTCGAAAAGAG--GTGTTGTACAA   372
                            ** * :: .* *. *** : .:**  .  ***.   *  .*.. :   ** *** *.*.

SGD_Scer_GDB1/YPR184W      AAAGCAGAACATACAGCCCGGTTGAATAGCATGAGTCATGGTTGCCGAAGAGGACATCGA   6197
WASHU_SBAY_CONTIG654.29    AACTAAAGATATACATCTCGATCGAGTAGCATGAATCATGGTTGCCAATCTGGATAACGA   6181
6033_chrXVI_int            AATAATAGACATACCACTCGATCGAGTAGTATGAATCATGGTTGCCAATCTGAATAACGA   432
                           **  .:..* ****. * **.* **.*** ****.***********.*: :*.* *:***

SGD_Scer_GDB1/YPR184W      GAAGCAAGTCCTTCAATTGATAGACAGCTTTTTTCTGAAGACTACACTACTAATATGCTC   6257
WASHU_SBAY_CONTIG654.29    GAAAGAGGTTCTCCAATTGATAGATAGTTTTTTTCTTGAAAGCTACATTGCTAATATGCTC   6241
6033_chrXVI_int            GAAAGAGGTTCTTCAATTGATAGATAGTTTTTTTCTTGAAAGCAACATTGCTAATATGTTC   492
                           ***. *.** ** *********** ** *****  ****..*:*** *.******** **

SGD_Scer_GDB1/YPR184W      CACCGAATCAAGTCGATACCAGTCTTCTACAGAAAATATATTCCTATTTGACGACACATG   6317
WASHU_SBAY_CONTIG654.29    TACAGAATCGAGCCGATACCAGTCTTCTACGGAGAATATACCTTTATTTGACGATACATG   6301
6033_chrXVI_int            TACAGNNNNGAGCCGATACCAATCTTCTACGGAG--------------------------   526
                            **.*   . .** ********.********.**.
```

PMA2/YPL036w, *Se> Sc* at 121-134bp

```
SGD_Scer_PMA2/YPL036W    ------------------------------------------------------------    0
FM1318/1-2805            ------------------------------------------------------------    0
6033_YPL036W             aatnngGCACGAGtGcgGATTAGgGAaGGCAgtgACAAAAAGAAACCCATGCAAGAATAA    60

SGD_Scer_PMA2/YPL036W    ------------------------------------------------------------    0
FM1318/1-2805            ------------------------------------------------------------    0
6033_YPL036W             GAAAGCATTGCTGCAAGGAAAAGGAAAGCCGGCGTAAGGTcTCCTTTACTCCAAAAATCG    120

SGD_Scer_PMA2/YPL036W    ------------------------------------------------------------    0
FM1318/1-2805            ------------------------------------------------------------    0
6033_YPL036W             TCGAGGGAGAACAAaAAGAATntctTGTTTAAGGGAATTGCAAAGTCAATtctCGGGGCA    180
```

```
SGD_Scer_PMA2/YPL036W     ------------------------------------------------------------    0
FM1318/1-2805             ------------------------------------------------------------    0
6033_YPL036W              GCCTGCACTGAAAAAGGGAGGATGAACGTCGACGAACGAATGTTGAAGGGGGAAATGGTT    240

SGD_Scer_PMA2/YPL036W     ------------------------------------------------------------    0
FM1318/1-2805             ------------------------------------------------------------    0
6033_YPL036W              ATCAATGGACAAACACATAGCAATGAACACACTCTTGTCAACGTCCCCTAAACTGTGATT    300

SGD_Scer_PMA2/YPL036W     ------------------------------------------------------------    0
FM1318/1-2805             ------------------------------------------------------------    0
6033_YPL036W              GGTAGCAGTGGTGATGGTTCGCTTACTGTACTTGCGCTTATATAAAGGCGTATCGTGTCT    360

SGD_Scer_PMA2/YPL036W     ------------------------------------------------------------    0
FM1318/1-2805             ------------------------------------------------------------    0
6033_YPL036W              CCTTTCCTGTCCATCTCTCTTGTTCTTTCTGTATGGCGTATGCTGCTCAAGCTACTGTGC    420

SGD_Scer_PMA2/YPL036W     ----------ATGTCTTCCACTGAAGCAAAGCAATACAAGGA---GAAACCCTC------    41
FM1318/1-2805             ----------ATGTCTTCTAGTGGTGC---------------------------------    17
6033_YPL036W              TTTCTTTTTCATTTTTAACTGTGTAGCATTGCGATTCAAGAAAGAGAAAACATTTTTGTA    480
                                    ** * *:.  : ** :**

SGD_Scer_PMA2/YPL036W     ----GAAAGAGTACCTCCATGCCAGTGATGGCGATGACCCTGCAAATA---ATTCTGCCG    94
FM1318/1-2805             -------------------AAAGCCATACCGAGAGAAGCCTGCAGCGA---ACAATGGTG    55
6033_YPL036W              TTATGTCTTCTAGTGGTGCAAAGCCATACCGAGAGAAGCCTGCAGCGAACAATGGTGCTT    540
                                             :.. . : *  *.** .* ******.. *   *   **

SGD_Scer_PMA2/YPL036W     CTTCTTCGTCATCTTCGTCTTCTACATCAACTTCCGCCTCGTCATCGGCTGCAGCCGTTC    154
FM1318/1-2805             CTTCGTCNTCNTCGTCTTCGTCTTCTTCTACATCGGCCTCGCCATCGCCTGCAGCCGCTC    115
6033_YPL036W              CGTCCTCGTCTTCGTCTTCGTCTTCTTCTACATCGGCCTCGCCATCGCCTGCAGCCGCTC    600
                          * ** ** ** ** ** ** ***:*:**:**:** ****** ***** ********* **

SGD_Scer_PMA2/YPL036W     CACGGAAGGCCGCAGCCGCTTCTGCCGCTGATGATTCTGACTCAGATGAAGATATAGACC    214
FM1318/1-2805             CACGTAAGGCCGCAGCCGTGCCTGCTGCTAACGACTCCGACTCCGATGAGGATATTGATG    175
6033_YPL036W              CACGTAAGGCCGCAGCCGCTTCTGCCGCTGATGATTCTGACTCAGATGAAGATATAGACC    660
                          **** *************   **** ***.* ** ** *****.*****.*****:**

SGD_Scer_PMA2/YPL036W     AATTGATTGATGAACTACAATCTAACTACGGTGAGGGTGATGAATCTGGTGAAGAAGAAG    274
FM1318/1-2805             GATTGATTGAAGAATTGCAATCGAACTACGGAGAGAGCGAAGAATCCAGTGAAGAAGAGA    235
6033_YPL036W              AATTGATTGATGAACTACAATCTAACTACGGTGAGGGTGATGAATCTGGTGAAGAAGAAA    720
                          .*********:*** *.***** ********:***.* **:***** .**********..

SGD_Scer_PMA2/YPL036W     TACGTACTGATGGGGTGCACGCTGGCCAAAGGGTTGTTCCTGAAAAGGACCTTTCTACGG    334
FM1318/1-2805             AGCATACTGATGAGGCCCACGCTGGTCAAAGGGTGATTCCCGAAAAGGACCTTTCGACAG    295
6033_YPL036W              TACGTACTGATGGGGTGCACGCTGGCCAAAGGGTTGTTCCTGAAAAGGACCTTTCTACGG    780
                          :.*.********.**  ******** ******** .**** ************** **.*

SGD_Scer_PMA2/YPL036W     ACCCTGCGTATGGTTTGACTTCGGATGAAGTCGCCAGGAGAAGAAAGAAATATGGGTTAA    394
FM1318/1-2805             ATCCCGCCTATGGTCTGACTTCGGACGAAGTCACCAGGAGAAGAAAGAAGTACGGTTTGA    355
6033_YPL036W              acCCTGCGTATGGTTTGACTTCGGATGAAGTCGCCAGGAGAAGAAAGAAATATGGGTTAA    840
                            ** ** ****** ********** ******.****************.** ** **.*

SGD_Scer_PMA2/YPL036W     ATCAAATGGCTGAGGAGAATGAATCGTTGATTGTGAAGTTTTTGATGTTCTTCGTAGGGC    454
FM1318/1-2805             ATCAAATGGCTGAAAACAATGAATCTTTAGTTGTTAAATTCATCATGTTTTTCGTNGGNC    415
6033_YPL036W              ATCAAATGGCTGAGGAGAATGAATCGTTGATTGTGAAGTTTTTGATGTTCTTCGTAGGGC    900
                          *************..* ******** **..**** **.** :* ***** ***** ** *

SGD_Scer_PMA2/YPL036W     CTATTCAATTCGTTATGGAGGCTGCTGCTATTTTGGCTGCCGGTTTGTCTGATTGGGTTG    514
FM1318/1-2805             CAATNCAATTCGTTATGGAAGCAGCCGCTATCTTGGCTGCCGGTTTGTCTGATTGGGTCG    475
6033_YPL036W              CGATTCAATTCGTTATGGAGGCAGCTGCTATTTTGGCTGCCGGTtgtcTGAttgGtnGnA    960
                          * **.**************.**:** ***** ************      .   *    .

SGD_Scer_PMA2/YPL036W     ATGTCGGTGTCATCTGTGCTTTACTGCTATTAAACGCATCTGTCGGATTTATTCAAGAAT    574
FM1318/1-2805             ATTTCGGTGTCATCTGTGCCCTACTACTGTTGAATGCTTCGGTCGGGTTCATCCAAGAGT    535
6033_YPL036W              t-----------------------------------------------------------    961
```

DBVPG 6261

UTP4/YDR324c, *Se> Sc* at 861- 876bp *

```
SGD_Scer_UTP4/YDR324C       TCCATTAAATTCTGGGATTTCCAGTTTGCCACGCTAAACCAGTCATTTAAGGCGCACGAT     840
MIT_Sbay_c442_4963          TCCATAAAGTTCTGGGATTTCCAATTCGCTACATTGAACCAGTCTTTTAAAGCACATGAT     768
6261_YDR324C                ---------------------------------------------------GCACATGAT     9
                                                                               **.** ***

SGD_Scer_UTP4/YDR324C       GCAGACGTACTGTGTCTAACTACCGATACTGATAATAATTATGTTTTTAGTGCTGGTGTG     900
MIT_Sbay_c442_4963          GCAGACGTCTTATGCCTAACTACTGATATTGATAACAATTACGTGTTCAGTGCTGGTGTG     828
6261_YDR324C                GCAGacgtctTAtgccTAACTActGATATTGaTAACAATTACGTgttTAGTgatGGTGTG     69
                            ****      *.    ******  **** ** *** ***** **    ***   ******

SGD_Scer_UTP4/YDR324C       GACAGAAAAATCTTTCAATTTTCTCAAAACACTAACAAATCTCAAAAGAACAACAGATGG     960
MIT_Sbay_c442_4963          GATAGAAAAATCTTCCAATTCTCCCAAAATAGCAACAAATCTCAAAAAAACAACAGATGG     888
6261_YDR324C                GATAGAAAGATCTTCCAATTTTCTCAAAATAGCAACAAATCTCAAAAGAACAACAGATGG     129
                            ** *****.***** ***** ** ***** *  **************.************

SGD_Scer_UTP4/YDR324C       GTAAATTCTTCTAATAGGTTGCTTCATGGAAACGACATTAGAGCAATATGTGCATACCAA     1020
MIT_Sbay_c442_4963          GTGAACTCTTCCAATAGGTTACTTCATGGTAACGAA------------------------     924
6261_YDR324C                GTAAATTCTTCTAATAGGTTGCTTCATGGAAACGACATTAGAGCAATATGTGCATACCAA     189
                            **.** ***** ********.********:*****.

SGD_Scer_UTP4/YDR324C       TCTAAAGGTGCAGATTTTCTAGTTTCAGGAGGTGTTGAAAAAACACTAGTCATCAACTCA     1080
MIT_Sbay_c442_4963          ------------------------------------------------------------     924
6261_YDR324C                TCTAAAGGTGCAGATTTTCTAGTTTCAGGAGGTGTTGAAAAAACACTAGTCATCAACTCA     249

SGD_Scer_UTP4/YDR324C       CTTACTTCTTTTTCTAATGGAAACTACAGGAAGATGCCAACTGTCGAACCTTATTCAAAG     1140
MIT_Sbay_c442_4963          ------------------------------------------------------------     924
6261_YDR324C                CTTACTTCTTTTtctAATGGAAACTACAGGAAGATGCCAACTGTcgAAcctTATTCAAAG     309

SGD_Scer_UTP4/YDR324C       AATGTTTTAGTTAACAAAGAGCAACGCCTTGTTGTTTCATGGAGCGAATCTACTGTTAAG     1200
MIT_Sbay_c442_4963          ------------------------------------------------------------     924
6261_YDR324C                AATGTTTTAGTTAACAAAGAGCAACGCCTTGTTGTTTCATGgagagAAtcTACTGTtaag     369

SGD_Scer_UTP4/YDR324C       ATATGGACAATGGGAACCGATTCTAGTACAGAACAGAATTATAAGCTAGTTTGCAAGTTA     1260
MIT_Sbay_c442_4963          ------------------------------------------------------------     924
6261_YDR324C                ATAtgGACAATGG-----------------------------------------------     382
```

CHD1/YER164w, *Se> Sc* at 1848-1863bp

```
SGD_Scer_CHD1/YER164W       TTTGAGAAGTTAAGCGTGCAACCTCCGTTCATTAAAGGTGGGGAATTAAGAGATTTTCAA     1140
FM1318/1-4407               TTCGAGAAGTTAAGTGTACAGCCACCTTTCATAAAGGGCGGAGAACTGAGAGATTTCCAA     1134
6261_YER164W                -------------------AGCCACCTTTCATAAAGGGCGGAGAACTGAGAGATTTCCAA     41
                                               *.**:** *****:**.** **.*** *.******** ***

SGD_Scer_CHD1/YER164W       CTAACTGGTATTAATTGGATGGCATTTTTGTGGTCCAAAGGTGATAATGGTATACTGGCA     1200
FM1318/1-4407               CTAACCGGTATCAATTGGATGGCATTTTTATGGTCTAAAGGCGATAATGGTATCTTAGCA     1194
6261_YER164W                CTAACCGGTACTAATtGGATGGCATTnTTATGGTCTAAAGGCGATAATGGTAtCTTAGCA     101
                            ***** ****  *** ********** **.***** ***** ********** . *.***

SGD_Scer_CHD1/YER164W       GATGAGATGGGCCTGGGAAAAACGGTCCAGACTGTCGCCTTTATCAGTTGGCTGATATTT     1260
FM1318/1-4407               GACGAAATGGGGCTAGGAAAAACCGTTCAGACCGTGGCATTTATCAGTTGGCTGATTTTT     1254
6261_YER164W                GACGAAATGGGGCTAGGAAAAACCGTTCAGACCGTGGCATTTATCAGTTGGCTGATTnTT     161
                            ** **.***** **.******** ** ***** ** **.*****************: **

SGD_Scer_CHD1/YER164W       GCTCGTAGACAAAACGGACCTCACATCATTGTCGTTCCTTTATCGACAATGCCTGCCTGG     1320
FM1318/1-4407               GCACGTAGGCAAAACGGTCCTCACATTGTTGTGGTTCCTTTGTCTACAATGCCTGCTTGG     1314
6261_YER164W                GCACGTAGGCAaAACGGTCCTCACATTGTTGTGGTTCCTTTGTCTACAATGCCTGCTTGG     221
                            **:*****.** *****:******** .**** ********.** *********** ***

SGD_Scer_CHD1/YER164W       TTGGATACTTTTGAGAAATGGGCGCCTGATTTGAATTGTATATGCTATATGGGCAACCAA     1380
FM1318/1-4407               TTAGATACATTTGAGAGATGGTCACCTGATTTGAATTGTATTTGCTATATGGGTAACCAA     1374
6261_YER164W                TTAGATACATTTGAGAGATGGTCACCTGATTTGAATTGTATTTGCTATATGGGTAACCAA     281
                            **.*****:*******.**** *.*****************:*********** ******
```

```
SGD_Scer_CHD1/YER164W      AAATCAAGAGATACCATTCGAGAATATGAATTTTACACCAATCCAAGGGCAAAAGGGAAA      1440
FM1318/1-4407              AAATCAAGAGATACCCTTAGGGAATACGAATTCTACACCAATCCACAGGCAAAGGGGAAG      1434
6261_YER164W               AAATCAAGAGATACCCTTAGGGAATACGAATTCTACACCAATCCACAGGCAAAGGGGAAG      341
                           ***************.**.*.***** ***** ************..******.*****.

SGD_Scer_CHD1/YER164W      AAAACAATGAAATTTAATGTTTTATTAACAACATACGAGTACATCTTAAAGGATCGTGCT      1500
FM1318/1-4407              AAAACAATGAAATTTAACGTTTTATTAACAACATACGAATATATCTTAAAGGACCGTGCT      1494
6261_YER164W               AAAACAATGAAATTTAACGTTTTATTAACAACATACGAATATATCTTAAAGGACCGTGCT      401
                           ***************** ********************.** *********** ******

SGD_Scer_CHD1/YER164W      GAATTAGGAAGTATAAAATGGCAATTTATGGCCGTTGACGAAGCTCATAGACTAAAAAAT      1560
FM1318/1-4407              GAATTAGGTGGCATCAAATGGCAATTTATGGCCGTTGATGAAGCTCATAGATTAAAAAAT      1554
6261_YER164W               GAATTAGGTGGCATCAAATGGCAATTTATGGCCGTTGATGAAGCTCATAGATTAAAAAAC      461
                           ********:.* **.*********************** ************ *******

SGD_Scer_CHD1/YER164W      GCTGAATCATCCCTTTATGAATCATTAAACAGTTTCAAGGTCGCCAACCGTATGTTAATC      1620
FM1318/1-4407              GCAGAATCATCTCTTTATGAATCATTGAACAGTTTCAAGGCTGCGAACCGTATGTTGATC      1614
6261_YER164W               GCAGAATCATCTCTTTATGAATCATTGAACAGTTTCAAGGCTGCGAACCGTATGTTGATC      521
                           **:******** **************.*************  ** ***********.***

SGD_Scer_CHD1/YER164W      ACAGGCACACCTCTTCAGAATAATATTAAAGAGTTAGCTGCGTTGGTTAATTTCCTAATG      1680
FM1318/1-4407              ACAGGTACTCCTCTTCAAAATAATATTAAAGAACTGGCTGCATTAATTAATTTCTTAATG      1674
6261_YER164W               ACAGGTACTCCTCTTCAAAATAATATTAAAGAACTGGCTGCATTAATTAATTTCTTAATG      581
                           ***** **:********.**************. *.*****.**..******** *****

SGD_Scer_CHD1/YER164W      CCCGGAAGGTTTACGATTGATCAGGAGATTGATTTTGAAAACCAAGATGAAGAGCAAGAA      1740
FM1318/1-4407              CCTGGAAGATTTACTATTGATCAAGAAATTGATTTTGAGAATCAAGATGCCGAACAAGAA      1734
6261_YER164W               CCTGGAAGATTTACTATTGATCAAGAAATTGATTTTGAGAATCAAGATGCCGAACAAGAA      641
                           ** *****.***** ********.**.***********.** *******..**.******

SGD_Scer_CHD1/YER164W      GAATATATTCATGATTTACACCGAAGAATACAGCCTTTTATTCTTCGTCGGTTGAAGAAA      1800
FM1318/1-4407              GAATATATCCATGATTTACATAGGAGGCTTCAGCCTTTCATTCTTCGTCGTTTAAAGAAA      1794
6261_YER164W               GAATATATCCATGATTTACATAGGAGGCTTCAGCCTTTCATTCTTCGTCGTTTAAAGAAA      701
                           ******** *********** .*.**..*:********* *********** **.******

SGD_Scer_CHD1/YER164W      GACGTAGAAAAATCACTTCCATCAAAGACAGAGCGTATTTTAAGAGTTGAATTGTCCGAC      1860
FM1318/1-4407              GATGTAGAAAAGTCATTGCCTTCAAAGACAGAACGTATTTTGAGAGTCGAACTATCCGAC      1854
6261_YER164W               GATGTAGAAAAGTCATTGCCTTCAAAGACAGAACGTATTTTGAGAGTCGAACTATCCGAC      761
                           ** ********.*** * **:***********.********.***** *** *.******

SGD_Scer_CHD1/YER164W      GTACAGACTGAGTACTATAAAAATATTCTGACTAAAAACTACTCTGCTTTAACTGCTGGA      1920
FM1318/1-4407              GTACAGACGGAGTACTATAAGAATATTTTAACAAAAAACTATTCTGCTCTTACTGCTGGT      1914
6261_YER164W               GTACAGACTGAGTACTATAAAAATATTCTGACTAAAAACTACTCTGCTTTAACTGCTGGA      821
                           ******** ***********.****** *.**:********* ****** *:********:

SGD_Scer_CHD1/YER164W      GCTAAAGGGGGTCATTTCTCTTTACTGAATATTATGAACGAGTTGAAAAAGGCATCGAAC      1980
FM1318/1-4407              CCTAAGGGTGGTCACTTTTCTTTATTGAATATTATGAATGAACTGAAAAAGGCATCGAAC      1974
6261_YER164W               GCTAAAGGGGGTCATTTCTCTTTACTGAATATTATGAACGAGTTGAAAAAGGCATCGAAC      881
                            ****.** ***** ** ****** ************* **. *****************

SGD_Scer_CHD1/YER164W      CATCCATATCTCTTCGATAATGCTGAAGAGCGCGTCTTACAGAAATTTGGGGATGGTAAA      2040
FM1318/1-4407              CATCCGTATCTATTCGACAATGCTGAAGACCGGGTTTTACAAAGTTTGGAGATGGTAAA      2034
6261_YER164W               CATCCATATCTCTTCGATAATGCTGAAGAGCGCGTCTTACAGAAATTTGGGGATGGTAAA      941
                           *****.*****.***** *********** ** ** *****.**.*****.*********

SGD_Scer_CHD1/YER164W      ATGACTCGAGAAAACGTACTAAGAGGTTTGATCATGTCTTCGGGTAAGATGGTTCTTTTA      2100
FM1318/1-4407              ATGACCCGTGAAAACATTTTAAGAGGGCTGATCATGTCTTCAGGTAAAATGGTTCTTTTG      2094
6261_YER164W               ATGACTCGAGAAAACGTACTAAGAGGTTTGATCATGTCTTCGGGTAAGATGGTTCtTTTA      1001
                           ***** **:******.*: *******  **************.*****.******* ***.

SGD_Scer_CHD1/YER164W      GACCAATTATTGACCAGATTGAAGAAAGATGGGCACCGCGTGTTGATTTTTTCACAAATG      2160
FM1318/1-4407              GATCAATTATTGACCAGATTGAAGAAAGATGGACATCGTGTGTTAATTTTCTCTCAAATG      2154
6261_YER164W               GACCAATtAtGACCAGAtGaAgnAAG----------------------------------      1027
                           ** **** *   ..*...    .   .*.
```

KEM1/YGL173c, *Se> Sc* at 462-477bp

```
SGD_Scer_XRN1/YGL173C      --------ATGGGTATTCCAAAATTTTTCAGGTACATCTCAGAAAGATGGCCCATGATTT      52
FM1318/1-4548              --------ATGGGTATTCCAAAATTTTTCAGGTACATCTCAGAAAGATGGCCCATGATTC      52
6261_YGL173C               AGTACAGCATGGGTATTCCGAAATTTTTCAGGTACATCTCAGAAAGATGGCCCATGATTC      180
                                   ***********.***************************************

SGD_Scer_XRN1/YGL173C      TACAGCTTATTGAGGGAACACAGATTCCTGAGTTTGATAACTTATACCTGGATATGAATT      112
FM1318/1-4548              TACAACTTATTGAAGGGACTCAGATTCCCGAGTTTGATAACCTATATCTGGATATGAATT      112
6261_YGL173C               TACAACTTATTGAAGGGACTCAGATTCCTGAGTTTGATAACCTATATCTGGATATGAATT      240
                           ****.********.**.**:******** ************ **** *************

SGD_Scer_XRN1/YGL173C      CGATTTTACATAATTGTACGCATGGTAACGACGATGATGTAACCAAGCGATTAACTGAAG      172
FM1318/1-4548              CGATTTTACATAACTGTACACATGGTAACGACGATGACGTGACGAAGCGATTAACTGAAG      172
6261_YGL173C               CGATTTTACATAACTGTACACATGGTAACGACGATGACGTGACGAAGCGATTAACTGAAG      300
                           ************* *****.***************** **.** ****************

SGD_Scer_XRN1/YGL173C      AAGAGGTTTTTGCAAAAATCTGTACGTATATCGATCACCTTTTTCAAACAATCAAGCCCA      232
FM1318/1-4548              AAGAGGTTTTTGCAAAGATCTGCACGTATATTGATCATCTTTTTCAAACTATCAAGCCCA      232
6261_YGL173C               AAGAGGTTTTTGCAAAGATCTGCACGTATATTGATCATCTTTTTCAAACTATCAAGCCCA      360
                           ****************.***** ******** ***** ***********:**********

SGD_Scer_XRN1/YGL173C      AGAAGATTTTCTACATGGCTATTGATGGTGTGGCCCCTCGTGCCAAGATGAATCAACAAA      292
FM1318/1-4548              AGCAAATTTTTTACATGGCTATCGATGGTGTGGCCCCCCGTGCGAAGATGAATCAACAGA      292
6261_YGL173C               AGCAAATTTTTTACATGGCTATCGATGGTGTGGCCCCCCGCGCGAAGATGAATCAACAGA      420
                           **.*.***** *********** ************** ** ** **************.*

SGD_Scer_XRN1/YGL173C      GAGCTCGTAGATTCAGAACCGCTATGGATGCAGAAAAAGCCTTGAAGAAGGCTATTGAGA      352
FM1318/1-4548              GAGCTCGTAGATTTAGAACTGCCATGGACGCCGAAAAAGCTATGAAAAAAGCTATTGAAA      352
6261_YGL173C               GAGCTCGTAGATTTAGAACTGCCATGGACGCCGAAAAAGCTATGAAAAAAGCTATTGAAA      480
                           ************* ***** ** ***** **.******** :****.**.********.*

SGD_Scer_XRN1/YGL173C      ATGGTGACGAGATTCCTAAGGGTGAGCCATTTGATTCGAATTCTATTACTCCAGGTACGG      412
FM1318/1-4548              ATGGTGACGAAATTCCTAAAGGTGAGCCGTTCGACTCAAACTGTATTACTCCAGGTACTG      412
6261_YGL173C               ATGGTGACGAAATTCCTAAAGGTGAGCCGTTCGACTCAAACTGTATTACTCCAGGTACTG      540
                           **********.********.********.** ** **.** * *************** *

SGD_Scer_XRN1/YGL173C      AGTTTATGGCCAAATTGACCAAAAACTTACAATATTTTATTCACGACAAGATTTCTAACG      472
FM1318/1-4548              AATTTATGGCTAAACTGACAAAAAAATTTACAGTATTTCATTCATGACAAAATTTCTAACG      472
6261_YGL173C               AATTTATGGCTAAACTGACAAAAAAATTTACAGTATTTCATTCATGACAAAATTTCTAACG      600
                           *.******** *** ****.***** ***** *****.***** *****.**********

SGD_Scer_XRN1/YGL173C      ATTCCAAATGGAGGGAAGTGCAAATCATATTTTCTGGCCATGAAGTTCCAGGTGAAGGTG      532
FM1318/1-4548              ATTCAAAGTGGAGAGAAGTGCAAATCATATTTTCTGGCCATGAAGTTCCAGGTGAAGGTG      532
6261_YGL173C               ATTCCAAATGGAGGGAAGTGCAAATCATATTTTCTGGCCATGAAGTTCCAGGTGAAGGTG      660
                           ****.**.*****.**********************************************

SGD_Scer_XRN1/YGL173C      AACACAAGATCATGAACTTTATAAGGCATTTAAAATCCCAAAAGGATTTCAACCAGAATA      592
FM1318/1-4548              AGCACAAAATTATGAATTTCATAAGGCATTTAAAATCCCAGAAGGATTTCAACCAAAATA      592
6261_YGL173C               AACACAAGATCATGAACTTTATAAGGCATTTAAAatcCCaAAAGGAtTCAACCAGAatAc      720
                           *.*****.** ***** ** **************   ** .***** * .*.*...  :

SGD_Scer_XRN1/YGL173C      CGAGACATTGTATTTACGGTCTTGACGCAGATTTGATTATGCTGGGTTTGTCTACTCATG      652
FM1318/1-4548              CGAGACACTGTATTTATGGTCTGGATGCGGATTTGATTATGCTTGGTTTATCTACTCACG      652
6261_YGL173C               nAGaCAtntA--------------------------------------------------      730
                            .. ..   .
```

TOR2/YKL203c, *Sc> Se* at 3164-3179bp

```
SGD_Scer_TOR2/YKL203C      GTAGTTATCCACAATCTGATGAAGATATTGAATGATCCATCGTTGTCAATCCATCACACG      2820
FM1318/1-7422              GTAGTCATTCATAACCTTATGAAGATTTTAAATGACCCGTCTTTATCAAGTCATCATAC-      2819
6261_YKL203C               ------------AATCTGATGaAGATATTGAATGATCCATCGtTGTCAATCCATCACAcn      48
                                       ** ** *** ****:**.***** **.**  *.****  ***** *
```

```
SGD_Scer_TOR2/YKL203C      -GCTGCTATTCAAGCTATTATGCATATTTTTCAAAACCTTGGTTTACGATGTGTCTCCTT      2879
FM1318/1-7422              AGCGTCTATTCAAGCAATTATGCATATCTTTCAAAGCCTAGGTTTACGTTGTGTCTCATT      2879
6261_YKL203C               ncctgcnaTtCAAGCTATTATGCATATTTTTCAAAACCTTGCTTTACGATGTGTCTCCTT      108
                                   * *****:*********** *******.***:* ******:********.**

SGD_Scer_TOR2/YKL203C      TTTGGATCAAATTATTCCAGGTATCATTTTAGTCATGCGTTCATGCCCGCCGTCCCAACT      2939
FM1318/1-7422              TCTAGATCAGATAATTCCAGGTATCATCTTGGTGATGCGTTCATGTCCACCATCACAACT      2939
6261_YKL203C               TTTGGATCAAATTATTCCAGGTATCATTTTAGTCATGCGTTCGTGCCCGCTGTCCCAACT      168
                           * *.*****.**:************** **.** ********.** **.*  .**.*****

SGD_Scer_TOR2/YKL203C      TGACTTTTATTTTCAGCAACTGGGATCTCTCATCTCAATTGTCAAGCAACATATTAGGCC      2999
FM1318/1-7422              GGAATTCTATTTTCAACAACTTTGTTCACTTGTATCAATTGTCAAACAGCACATTAGGCC      2999
6261_YKL203C               TGACTTTTATTTTCAGCAACTGGGATCTCTCATCTCAATTGTCAAGCAACATATTAGGCC      228
                            **.** ********.*****   *:**:**  .*.***********.**.** ********

SGD_Scer_TOR2/YKL203C      CCATGTCGAGAAAATTTATGGTGTGATCAGGGAGTTTTTCCCGATCATTAAACTACAAAT      3059
FM1318/1-7422              TCATGTGGAGAAAATATATAGTGTAATCAAGGAATTTTTCACAATCATTAAATTACAAAT      3059
6261_YKL203C               CCATGTCGAGAAAATTTATGGTGTGATCAGGGAGTTTTTCCCGATCATTAAACTACAAAT      288
                            ***** ********:***.****.****.***.******.*.********* *******

SGD_Scer_TOR2/YKL203C      CACAATTATTTCTGTCATAGAATCGATATCTAAGGCTCTGGAAGGTGAGTTTAAAAGATT      3119
FM1318/1-7422              CACAATAATATCGGTTATTGAATCGATATCAAAAGCGTTGGAAGGTGAGTTCAAGAGATT      3119
6261_YKL203C               CACAATTATTTCTGTCATAGAATCGATATCTAAGGCTCTGGAAGGTGAGTTTAAAAGATT      348
                           ******:**:** ** **:***********:**.**  ************** **.*****

SGD_Scer_TOR2/YKL203C      TGTTCCCGAGACTCTAACCTTTTTCCTTGATATTCTTGAGAACGACCAGTCTAATAAAAG      3179
FM1318/1-7422              CGTTCCAGAAACTTTGACATTTTTCCTTGACATCCTTGAGAACGATCAGTCTAATAAAAA      3179
6261_YKL203C               TGTTCCCGAGACCCTAACCTTTTTCCTTGATATTCTTGAGAACGACCAGTCTAATAAAAA      408
                            *****.**.**  *.**.*********** ** **********  *************.

SGD_Scer_TOR2/YKL203C      GATCGTTCCGATTCGTATATTAAAATCTTTGGTTACTTTTGGGCCGAATCTAGAAGACTA      3239
FM1318/1-7422              AATTGTTTCTATCCGTATACTGAAGTCCTTAGTGACTTTTGGATCCAATTTAGAGGATTA      3239
6261_YKL203C               AATTGTTTCTATCCGTATACTGAAGTCCTTAGTGACTTTTGGATCCAATTTAGAGGATTA      468
                           .** *** * ** ****** *.**.** **.** ********. * *** ****.** **

SGD_Scer_TOR2/YKL203C      TTCCCATTTGATTATGCCTATCGTTGTTAGAATGACTGAGTATTCTGCTGGAAGTCTAAA      3299
FM1318/1-7422              CTCCCATTTAATTATGCCCGTAGTCGTCAGAATGACTGAATACTCCGCTGGAAGTTTAAA      3299
6261_YKL203C               CTCCCATTTAATTATGCCCGTAGTCGTCAGAATGACTGAATACTCCGCTGGAAGTTTAAA      528
                            ********.********  .*.** ** ***********.** ** ********* ****

SGD_Scer_TOR2/YKL203C      GAAAATCTCCATTATAACTTTGGGTAGATTAGCAAAGAATATCAACCTCTCTGAAATGTC      3359
FM1318/1-7422              AAAAATTGCAATCATTACGTTGGGGAGATTAGCAAAGAATATCAACCTCTCAGAAATGTC      3359
6261_YKL203C               AAAAaTTGCAATCATTACGTTGGGGAGATTAGCAAAGAATATCAACCTCTCAGAAATGTC      588
                           .*** *  *.** **:** ***** **************************:********

SGD_Scer_TOR2/YKL203C      ATCAAGAATTGTTCAGGCGTTGGTAAGAATTTTGAATAATGGGGATAGAGAACTAACAAA      3419
FM1318/1-7422              ATCAAGGATTGTTCAGGCACTGGTGCGAATCTTGAATAACGGGGACAGAGAACTAACAAA      3419
6261_YKL203C               ATCAAGGATTGTTCAGGCACTGGTGCGAATCTTGAATAacGGGGACAGAGAACTAACAAA      648
                           ******.***********. ****..**** ******* ***** ***************

SGD_Scer_TOR2/YKL203C      AGCAACCATGAATACGCTAAGTTTGCTCCTTTTACAACTAGGTACCGACTTTGTGGTCTT      3479
FM1318/1-7422              AGCAACCATGAATACATTGAGTTTGCTTCTTTTGCAATTGGGTACCGACTTCGTCATATT      3479
6261_YKL203C               agCaACCATGAATACATTGAGTTTGCTTCTttTGCAATTGGGTACCG-------------      695
                             * ***********. *.******** **  *.*** *.*******
```

VMA5/YKL080w, *Sc> Se* at 819-834bp

```
SGD_Scer_VMA5/YKL080W      TTCAAGTTAGATAAGTCTATCAAAGATTTGATAACATTGATTTCTAATGAATCTTCTCAA      420
FM1318/1-1179              TTTAAGTTGGATAAGTCTATTAAAGATTTGATTACATTGATTTCTGACGAATCATCTCAA      420
6261_YKL080W               ------------------atcAAAGAtTtgaTAACaTTGATTTCtAATGaAtCTTCTCAA      42
                                                ***** *   *:** ******** .* * * *:******

SGD_Scer_VMA5/YKL080W      TTAGACGCCGACGTCAGAGCTACTTATGCAAATTACAACAGCGCTAAAACTAACTTGGCT      480
FM1318/1-1179              TTGGATGCTGATGTGAGAGCTACATTTGCAAATTACAATAGTGCCAAAACCAACCTGGCT      480
6261_YKL080W               TTAGACGCCGACGTCAGAGCTACTTATGCAAATTACAACAGCGCTAAAACTAACTTGGCT      102
                           **.** ** ** ** ********:*:************ ** ** ***** *** *****

SGD_Scer_VMA5/YKL080W      GCTGCTGAGAGAAAGAAGACGGGTGACCTTTCTGTCAGATCCTTGCATGATATTGTCAAG      540
FM1318/1-1179              GCTGCTGAAAGAAAGAAAACTGGTGACCTTTCTGTTAGATCCTTACATGATATTGTCAAG      540
6261_YKL080W               GCTGCTGAGAGAAAGAAGACGGGTGACCTTTCTGTCAGATCCTTGCATGATATTGTCAAG      162
                           ********.********.** ************** ********.***************
```

```
SGD_Scer_VMA5/YKL080W      CCCGAAGACTTCGTTCTTAATTCTGAACATTTAACTACTGTTCTAGTAGCAGTTCCCAAA      600
FM1318/1-1179              CCAGAAAATTTTGTTCTACATTCTGAACACTTGACCACTGTTCTTGTAGCAGTTCCAAAA      600
6261_YKL080W               CCCGAAGACTTCGTTCTTAATTCTGAACATTTAACTACTGTTCTAGTAGCAGTTCCCAAA      222
                           **.***.* ** *****:.********** **.** ********:***********.***

SGD_Scer_VMA5/YKL080W      AGTTTAAAATCCGATTTCGAAAAATCGTACGAAACTTTATCCAAGAACGTTGTACCAGCA      660
FM1318/1-1179              AGTTTGAAATCTGATTTTGAGAATTGTTATGAAACTTTGTCCAAGAACGTTGTACCAGCA      660
6261_YKL080W               AGTTTAAAATCCGATTTCGAAAAATCGTACGAAACTTTATCCAAGAACGTTGTACCAGCA      282
                           *****.***** ***** **.**:*  ** ********.*********************

SGD_Scer_VMA5/YKL080W      TCTGCCAGCGTGATTGCAGAGGATGCTGAGTATGTTTTGTTCAATGTTCATTTGTTCAAG      720
FM1318/1-1179              TCTGCAGGTGTAATTGCCGAAGATGCTGAGTATGTCTTATTCAATGTTCACTTGTTCAAG      720
6261_YKL080W               TCTGCCAGCGTGATTGCAGAGGATGCTGAGTATGTTTTGTTCAATGTTCATTTGTTCAAG      342
                           *****..* **.*****.**.************** **.*********** *********

SGD_Scer_VMA5/YKL080W      AAAAACGTTCAAGAATTCACAACAGCTGCTAGAGAGAAGAAATTCATTCCTCGTGAATTT      780
FM1318/1-1179              AAAAATGTCCAAGAGTTCACAGCGGCTGCAAGGGAGAAGAAGTTTATCCCTCGTGAATTC      780
6261_YKL080W               AAAAACGTTCAAGAATTCACAACAGCTGCTAGAGAGAAGAAATTCATTCCTCGTGAATTT      402
                           ***** ** *****.******.*.*****:**.********.** ** ***********

SGD_Scer_VMA5/YKL080W      AACTACTCGGAGGAATTAATTGACCAGTTGAAAAAAGAGCATGACTCTGCTGCCAGTTTA      840
FM1318/1-1179              AATTACTCCGAGGAACTAATTGACCAATTGAAAAAGGAACATGACTCTGCTGCAAGTTTA      840
6261_YKL080W               AACTACTCGGAGGAATTAATTGACCAGTTGAAAAAAGAGCATGACTCTGCTGCAAGTTTA      462
                           ** ***** ****** **********.********.**.**************.******

SGD_Scer_VMA5/YKL080W      GAACAATCTTTGCGCGTCCAGTTGGTAAGATTGGCCAAGACAGCTTATGTCGATGTTTTT      900
FM1318/1-1179              GAGCAATCTTTACGTGTCCAACTAGTTAGATTGGCCAAGACTGCTTATGTTGATGTCTTC      900
6261_YKL080W               GAGCAATCTTTacgTGTCCAACTAGTTAGATTGGCCAAGACTGCTTATGTTGATGTCt--      520
                           **.********    *****. *.**:**************:******** *****

SGD_Scer_VMA5/YKL080W      ATAAATTGGTTCCACATCAAGGCCTTGAGAGTTTACGTGGAATCTGTTTTGCGTTACGGG      960
FM1318/1-1179              ATAAATTGGTTCCACATCAAGGCCTTGAGAGTGTACGTGGAATCCGTGTTGCGTTATGGT      960
6261_YKL080W               ------------------------------------------------------------      520
```

GAL80/YML051w, *Se> Sc* at 555-561bp

```
SGD_Scer_GAL80/YML051W     --------------------------------------------------ATGGACTACAAC      12
FM1318/1-1308              --------------------------------------------------ATGGACTATAAC      12
6261_YML051W               CACTTCCGTATACAAGTTCGACCTCTAGTTTTCTCGTGCCTTCCAGTCATGGACTATAAC     240
                                                                             ******** ***

SGD_Scer_GAL80/YML051W     AAGAGATCTTCGGTCTCAACCGTGCCTAATGCAGCTCCCATAAGAGTCGGATTCGTCGGT      72
FM1318/1-1308              AAGAGATCTTCCGTCTCTACAGTACCCAATGCAGCCCCCATAAGAGTCGGATTCATTGGC      72
6261_YML051W               AAGAGATCTTCCGTCTCTACAGTACCCAATGCAGCCCCCATAAGAGTCGGATTCATTGGC     300
                           *********** *****:**.**.** ******** ******************.* **

SGD_Scer_GAL80/YML051W     CTCAACGCAGCCAAAGGATGGGCAATCAAGACACATTACCCCGCCATACTGCAACTATCG     132
FM1318/1-1308              CTCAACGCTACCAAAGGGTGGGCGATCAAAACTCATTATCCCGCCATACTGCAGCTATCG     132
6261_YML051W               CTCAACGCTACCAAAGGGTGGGCGATCAAAACTCATTATCCCGCCATACTGCAGCTATCG     360
                           ********:.*******.*****.*****.**:***** **************.******

SGD_Scer_GAL80/YML051W     TCACAATTTCAAATCACTGCCTTATACAGTCCAAAAATTGAGACTTCTATTGCCACCATT     192
FM1318/1-1308              TCCCAATTTCAAATTACTGCCTTATACAGCCCAAAGATAGAGACCTCCATTGCCACTATC     192
6261_YML051W               TCCCAATTTCAAATTACTGCCTTATACAACCCAAAGATAGAGACCTCCATTGCCACTATC     420
                           **.*********** *************. *****.**:***** ** ******** **

SGD_Scer_GAL80/YML051W     CAGCGTCTAAAATTGAGTAATGCCACTGCTTTTCCCACTTTAGAGTCATTTGCATCATCT     252
FM1318/1-1308              CAACAGCTGAAACTCAGCAATGCCACGGCCTTCCCCACTTTAGAATCATTTGCATCTTCT     252
6261_YML051W               CAACAACTGAAACTTAGCAATGCCACGGCCTTCCCCACTTTAGAATCATTTGCATCTTCT     480
                           **.*. **.*** * ** ******** ** ** ***********.***********:***

SGD_Scer_GAL80/YML051W     TCCACTATAGATATGATAGTGATAGCTATCCAAGTGGCCAGCCATTATGAAGTTGTTATG     312
FM1318/1-1308              GCCACCGTGGACATGATAGTGATAACCATCCAGGTGGCCAGCCATTATGAGGTTCTAATG     312
6261_YML051W               GCCACCGTGGACATGATAGTGATAACCATCCAGGTGGCCAGCCATTATGAGGTTCTAATG     540
                            ****  .*.** ************.* *****.*****************.*** *:***
```

```
SGD_Scer_GAL80/YML051W      CCTCTCTTGGAATTCTCCAAAAATAATCCGAACCTCAAGTATCTTTTCGTAGAATGGGCC      372
FM1318/1-1308               CCCCTGTTGAAATACTCCCAAAATAATCCGAACCTCAAGTATCTTTTCGTAGAATGGGCC      372
6261_YML051W                CCCCTGTTGAAATACTCCCAAAATAATCCGAATCTCAAGTATCTTTTCGTAGAATGGGCC      600
                            ** ** ***.***:****.************* ***************************

SGD_Scer_GAL80/YML051W      CTTGCATGTTCACTAGATCAAGCCGAATCCATTTATAAGGCTGCTGCTGAACGTGGGGTT      432
FM1318/1-1308               CTTGCATGTTCTCTGGATCAAGCAGAATCGATTTATAAGGCAGCTGCCGAACGTGGACTA      432
6261_YML051W                CTTGCATGTTCTCTGGATCAAGCAGAATCGATTTATAAGGCAGCTGCCGAACGTGCACTA      660
                            ***********:**.********.***** ***********:***** ******* . *:

SGD_Scer_GAL80/YML051W      CAAACCATCATCTCTTTACAAGGTCGTAAATCACCATATATTTTGAGAGCAAAAGAATTA      492
FM1318/1-1308               CAAACTATTATTTCTTTACAAGGCCGTAAATCACCATACATCTTGAGAGCCAAAGAACTA      492
6261_YML051W                CAAACTATTATTTCTTTACAAGGCCGTAAATCACCATACATCTTGAGAGCCAAAGAACTA      720
                            ***** ** ** *********** ************** ** ********.****** **

SGD_Scer_GAL80/YML051W      ATATCTCAAGGCTATATCGGCGACATTAATTCGATCGAGATTGCTGGAAATGGCGGTTGG      552
FM1318/1-1308               ATTTCTGAAGGTTACATTGGCGACATCAACTCCATAGAAATTGCAGGAAACGGCGGTTGG      552
6261_YML051W                ATTTCTGAAGGTTACATTGGCGACATCAACTCCATAGAAATTGCAGGAAACGGCGGTTGG      780
                            **:*** **** ** ** ******** ** ** **.**.*****:***** *********

SGD_Scer_GAL80/YML051W      TACGGCTACGAAAGGCCTGTTAAATCACCAAAATACATCTATGAAATCGGGAACGGTGTA      612
FM1318/1-1308               TATGGCTATGAAAGACCCATCAAATCGCCCAACTATATTTATGAAATGGGCAATGGTGTA      612
6261_YML051W                TATGGCTACGAAAGGCCTGTTAAATCACCAAAATACATCTATGAAATCGGGAACGGTGTA      840
                            ** ***** *****.** .* *****.**.**.** ** ******** ** ** ******

SGD_Scer_GAL80/YML051W      GATCTGGTAACCACAACATTTGGTCACACAATCGATATTTTACAATACATGACAAGTTCG      672
FM1318/1-1308               GATCTGGTAACCACCACATTTGGCCACACCATTGACCTTTTGCAGTACATGACGAGCTCG      672
6261_YML051W                GATCTGGTAACCACAACATTTGGTCACACAATCGATATTTTACAATACATGACAAGTTCG      900
                            **************.******** *****.** ** .****.**.*********.** ***

SGD_Scer_GAL80/YML051W      TACTTTTCCAGGATAAATGCAATGGTTTTCAATAATATTCCAGAGCAAGAGCTGATAGAT      732
FM1318/1-1308               TATTTTTCTAGAATAAACGCAATGATGTTCAATAATATACCGGAGCAAGAGTTAATAGAC      732
6261_YML051W                TACTTTTCCAGGATAAATGCAATGGTTTTCAATAATAttCCAGAGCAAGAGCnGAtAGat      960
                            ** ***** **.***** ******.* **********  **.*********  .* **

SGD_Scer_GAL80/YML051W      GAGCGTGGTAACCGATTGGGCCAGCGAGTCCCAAAGACAGTACCGGATCATCTTTTATTC      792
FM1318/1-1308               GAATATGGTAACCGATTAGGTCAGCGAGTTCCGAAGACCGTACCCGATCACCTATTATTT      792
6261_YML051W                G-----------------------------------------------------------      961
                            *
```

FKS3/YMR306w, *Se> Sc* at 1551-1560bp

```
SGD_Scer_FKS3/YMR306W      CCTCGAGAATGGCCAGGCGCTCAGCATTTATCGAGTAGAATGATTGGCTTACTTTTTTGT      1260
FM1318/1-5358              CCTCGAGAATGGCCTGGCGCTCAACATTTATCACGAAGAATGATTGGCCTCCTTGTTTGT      1260
6261_YMR306W               --TcgAGAATGGCCTGGCGCTCaacATtnntcACGAAGAATGATTGGCCTCCTTGTTTGt      58
                             *  *********:*******    **      ..*:************ *.*** ****

SGD_Scer_FKS3/YMR306W      CTTGCAATCAATTTGGGACCTTCCGTGTATGTTCTGGGGTTTTTCGAATGGGATGTTCAT      1320
FM1318/1-5358              CTCGTAATCAATCTAGGACCATCCATTTATGTTTTGGGGTTTTTCGAATGGGATGTCCAT      1320
6261_YMR306W               ntCgTAATCAATTTAGGACCATCCATTTATGTTTTGGGGTTTTTCGAGTGGGATGTCCAT      118
                                ******* *.*****:***.* ****** *************.******** ***

SGD_Scer_FKS3/YMR306W      TCAAAATCTGCGTATATCGTGTCCATCGTCCAACTAATCATTGCATTTTTAACCACTTTT      1380
FM1318/1-5358              TCGAAATCAGCATATATCGTGTCGATCATTCAATTAATAATTGCACTTCTAACCACCCTT      1380
6261_YMR306W               tcgAAATCAGCATATATCGTGtCGATCATTCAATTAATAATTGCACTTCTAACCACCCTT      178
                              *****:**.********* * ***.* *** ****.****** ** *******  **

SGD_Scer_FKS3/YMR306W      TTTTTTGCTGTCAGACCTTTGGGCGGCTTATTTCGTCCATATTTGAATAAAGACAAAAAA      1440
FM1318/1-5358              TTTTTTGCTATCAGGCCCTTGGGCGGCCTATTTCGTCCATATCTGAATAAAGACAAAAGG      1440
6261_YMR306W               TTTTTtGCTATCAGGCCCTTGGGCGGCCTATTTCGTCCATATCTGAATAAAGACAAAAGG      238
                           ***** ***.****.** ********* ************** ***************..

SGD_Scer_FKS3/YMR306W      CATCGGAGGTACATCTCATCGCAAACCTTTACCGCTTCATTTCCTAAGCTGACAGGACGC      1500
FM1318/1-5358              CATCGAAGATACGTCTCATCTCAGACTTTTACCGCTTCGTTTCCCAAGCTGGCGGGAAGA      1500
6261_YMR306W               CATCGAAGATACGTCTCATCTCAGACTTTTACCGCTTCGTTTCCCAAGCTGGCAGGAAGA      298
                           *****.**.***.******* **.** ***********.***** ******.*.***.*.

SGD_Scer_FKS3/YMR306W      AGCAAATGGTTTTCTTATGGGTTATGGGTATTTGTGTATTTGGCGAAATATATTGAGTCC      1560
FM1318/1-5358              AGCAAATGGTTCTCTTACGGGCTATGGGTATTCGTATTTTTGGCAAAATACATTGAGTCT      1560
6261_YMR306W               AGCAAATGGTtctcTTACGGGCTATGGGTATTCGTATTTTTGGCAAAATACATTGAGTCC      358
                           **********    *** *** ********** **.*:******.***** ********
```

```
SGD_Scer_FKS3/YMR306W        TATTTTTTTTTGACCTTGTCCCTCAGGGACCCCATCAGGGTCCTCTCTATTATGGATTTG    1620
FM1318/1-5358                TACTTTTTTTTAACCTTATCCCTAAGGGACCCCATCCGGGTCCTGTCTATAATGGATCTA    1620
6261_YMR306W                 TAtTTTTTTTTGACCTTGTCCCTCAGGGACCCCATCAGGGTCCTCTCTATTATGGATTTG    418
                             ** ********.*****.*****.************.******* *****:****** *.

SGD_Scer_FKS3/YMR306W        TCCAGATGTCAAGGTGAATATTTGTTGGGTCCTATTCTATGTAAATGGCAAGCCAAAATT    1680
FM1318/1-5358                TCTAGATGTCAAGGTGAGTATTTATTGGGCCCTATACTGTGCAAATGGCAGGCCAAAATT    1680
6261_YMR306W                 tCCAGATGTCAAGGTGAATATTTGTTGGGTCCTATTCTATGTAAATGGCAAGCCAAAATT    478
                              * **************.*****.***** *****:**.** ********.*********

SGD_Scer_FKS3/YMR306W        ACATTAGTTCTCATGCTGCTTTCTGACTTGGGCCTGTTTTTTCTCGACACTTACCTTTGG    1740
FM1318/1-5358                ACGCTGGTGCTGATGTTACTTTCCGATTTGGGTTTATTCTTTCTCGACACTTACCTTTGG    1740
6261_YMR306W                 ACATTAGTTCTCATGCTGCTTTCTGACTTGGGCCTGTTTTTTCTCGACACTTACCTTTGG    538
                             **. *.** ** *** *.***** ** *****  *.** *********************

SGD_Scer_FKS3/YMR306W        TACATTATTTGCAACTGTATTTTTTCCATTGTACTGTCATTTTCTCTTGGTACTTCAATT    1800
FM1318/1-5358                TACATTATTTGTAATTGTGTTTTTTCCATCATACTGTCATTTTCTCTTGGTACTTCAATT    1800
6261_YMR306W                 TACATTATTTGCAACTGTATTTTTTCCATTGTACTGTCATTTTCCCTTGGTACTTCAATT    598
                             *********** ** ***.********** .************* ***************

SGD_Scer_FKS3/YMR306W        CTCACGCCATGGAAGAATGTATACTCTCGATTGCCAAAAAGGATATATTCCAAAATCCTT    1860
FM1318/1-5358                CTTACTCCCTGGAAGAACGTATATTCTCGAATGCCCAAGAGAATATATTCCAAAATCCTG    1860
6261_YMR306W                 CTCACGCCATGGAAGAATGTATACTCTAGATTGCCAAAAAGGATATATTCCAAAATCCTT    658
                             ** ** **.******** ***** ***.**:****.**.**.*****************

SGD_Scer_FKS3/YMR306W        GCTACTTCAGAGATGGATGTAAAATTTAAAGCAAAAATACTGATATCGCAGGTTTGGAAT    1920
FM1318/1-5358                GCTACTTCAGAAATGGATGTGAAATTTAAAGCAAAAATATTAATATCGCAAGTATGGAAT    1920
6261_YMR306W                 GCTACTTCAGAGATGGATGTAAAATTTAAAGCAAAAATACTGATATCGCAGGTTTGGAAT    718
                             ***********.********.****************** *.********.**:******

SGD_Scer_FKS3/YMR306W        GCCATTGTTATATCAATGTATAGGGAACATCTTCTCTCCATTGAACATTTACAAAGACTC    1980
FM1318/1-5358                GCCATCGTTATCTCTATGTATAGAGAACATCTTCTTTCCATTGAGCATTTACAGCGGCTA    1980
6261_YMR306W                 GCCATTGTTATATCAATGTATAGGGAACATCTTCTCTCCATTGAGCATTTACAAAGACTC    778
                             ***** *****.**:********.*********** ********.********..*.**.

SGD_Scer_FKS3/YMR306W        TTGTTTCAGCAAGTTGACTCCTTAATGGGAGACACAAGAACCCTGAAATCGCCTACATTT    2040
FM1318/1-5358                CTGTTCCAACAAGTGGATTCCCTAATGGGTGATACAAGAACTTTAAAATCCCCGACATTT    2040
6261_YMR306W                 TTGTTTCAGCAAGTTGACTCCTTAATGGGAGACACAAGAACCCTGAAATCGCCTACATTT    838
                              **** **.***** ** *** *******:** ********  *.***** ** ******

SGD_Scer_FKS3/YMR306W        TTCGTTGCACAAGATGATTCAACGTTCAAGTCAATGGAATTTTTTCCATCAAATTCAGAG    2100
FM1318/1-5358                TTTGTCGCCCAAGATGATTCAACTTTCAAATCAATGGAATTCTTTCCCACTAAATCAGAA    2100
6261_YMR306W                 TTCGTTGCACAAGATGATTCCACGTTCAAGTCAATGGAATTTTTTCCATCAAATTcagAG    898
                             ** ** **.***********.** *****.*********** *****.:*:**:*   *.

SGD_Scer_FKS3/YMR306W        GCAAAAAGAAGGATATCCTTTTTTGCCCAATCCCTGGCGACCCCCATTTCAGAACCTGTT    2160
FM1318/1-5358                GCGAAAAGAAGGATATCTTTCTTTGCTCAATCGTTGGCAACTCCTATCTCAGAACCTGTT    2160
6261_YMR306W                 GCAAAAAGAAGGATAtCCTTTTTTGcCCAatcCC-tGgCGACCCCCAtT-----------    945
                             **.************ * ** **  * .   *   * *.** ** *
```

ECM3/YOR092w, *Sc*> *Se* at 1722-1740bp

```
SGD_Scer_ECM3/YOR092W        AGATTGAAAATTGGAAAATTATACCCTGGTTTCTGGAAATCCGCAGTGGTATTAGTCTTT    1548
FM1318/1-1854                AGATTGAAAATTGGGAAATTGTATCCGGGTTTCTGGAAGGCTGCTGTGATCTTAGTGTTC    1560
6261_YOR092W                 ----------------------------------tggaatcCGCAGTGGTATTAGTCTTT    26
                                                                       **:***.*.***** **

SGD_Scer_ECM3/YOR092W        CTCAGACAATGTATCATGCCGATCTTTGGTGTCTTGTGGTGTGACCGTCTAGTGAAAGCG    1608
FM1318/1-1854                ATTAGACAATGTATCATGCCCATTTTTGGTGTCTTATGGTGCGATCGTCTGGTGAAAGCA    1620
6261_YOR092W                 CTCAGACAATGTATCATGccnnnctTTGGTGTCTTGTGGTGTGACCGTCTAGTGAAGGCA    86
                             .* ***************       **********.***** ** *****.*****.**.

SGD_Scer_ECM3/YOR092W        GGATGGCTAAATTGGGAAAACGACAAGATGTTATTGTTTGTTACCGCCATTACTTGGAAC    1668
FM1318/1-1854                GGTTGGTTGAACTGGGAAGACGACAAGATGTTACTGTTTGTTACTGCGATAACTTGGAAT    1680
6261_YOR092W                 GGATGGCTAAATTGGGAAAACGACAAGATGTTATTGTTTGTTACCGCTATTACTTGGAAC    146
                             **:*** *.** ******.************** ********** ** **:********
```

```
SGD_Scer_ECM3/YOR092W      TTACCAACAATGACCACCTTAATCTACTTCACTGCAAGTTATACCCCTGAGGACGAAACT      1728
FM1318/1-1854              TTGCCAACCATGACTACCCTAATCTATTTCACAGCGAGTTTCACCCCTGAAGATGAAACT      1740
6261_YOR092W               TTACCAACAATGACCACCTTAATCTACTTCACTGCAAGTTATACCCCTGAGGACGAAACT      206
                           **.*****.***** *** ******* *****:**.****: ********.** ******

SGD_Scer_ECM3/YOR092W      GAACCCGTTCAAATGGAATGTACCTCTTTCTTTTTGATGCTTCAATATCCTTTGATGGTC      1788
FM1318/1-1854              GAACCCGTTCAGATGGAATGCACTTCTTTCTTCTTGATGCTTCAGTATCCTCTGATGGTC      1800
6261_YOR092W               GAACCCGTTCAGATGGAATGCACTTCTTTCTTCTTGATGCTTCAGTATCCTCTGATGGTC      266
                           ***********.******** ** ******** ***********.****** ********

SGD_Scer_ECM3/YOR092W      GTTAGTTTACCATTTTTGGTGTCTTATTTCATCAAAGTTCAAATGAAATTATAA------      1842
FM1318/1-1854              GTTAGTTTNCCATTTTTGGTGTCTTATTTCATAAAGGTACAAATGAAACTATGA------      1854
6261_YOR092W               GTTAGTTTACCATTTTTGGTGTCTTATTTCATAAAGGTACAAATGAAACTATGATTTTCT      326
                           ******** **********************.**.**:********* ***.*
```

INP53/YOR109w, *Se> Sc* at 1137-1149bp

```
SGD_Scer_INP53/YOR109W     AGAGTACAAATTACAAGATCATTTGAAGCCACCCAACCGGTATTTGACAAACATATCATG      960
FM1318/1-3327              AGGGTACAAATTACTAGATCATTTGAAGCTACTCAGCCAGTGTTTGACAAGCACATTATG      960
6261_YOR109W               ------------------------------------------TTTGACAAGCACATTATG      18
                                                                     ********.** ** ***

SGD_Scer_INP53/YOR109W     AAATCAGTGGAAAAGTACGGACCTGTGCATGTCGTTAATTTGTTATCAACGAAATCTTCT      1020
FM1318/1-3327              AAGTCAGTAGAAAAATACGGCCCCGTTCACGTTGTCAACCTGTTATCAACAAAATCTTCA      1020
6261_YOR109W               AAGTCAGTAGAAAAATACGGCCCCgtTCACGTTGTCAACCTGtTATCAACAAAATCTTCA      78
                           **.*****.*****.*****.**    ** ** ** **  ** *******.********:

SGD_Scer_INP53/YOR109W     GAAATTGAACTTTCAAAACGATACAAGGAGCATTTAACTCATTCAAAAAAATTGAACTTC      1080
FM1318/1-3327              GAAATAGAACTTTCAAAGCGATACAAAGAGCATCTAACGCACTCAAAAAAGTTGAATTTC      1080
6261_YOR109W               GAAATAGAACTTTCAAAGCGATACAAAGAGCATCTAACGCACTCAAAAAAGTTGAATTTC      138
                           *****:***********.********.****** **** ** ********.***** ***

SGD_Scer_INP53/YOR109W     AACAAAGATATATTTTTGACAGAATTCGATTTTCATAAAGAAACTTCGCAAGAAGGCTTT      1140
FM1318/1-3327              AATAAAGATGTATTCTTAACAGAGTTCGATTTTCACAAAGAGACTTCTCAGGAAGGGTTT      1140
6261_YOR109W               AATAAAGATGTATTCTTAACAGAGTTCGATTTTCACAAAGAGACTTCTCAGGAAGGGTTT      198
                           ** ******.**** **.*****.*********** *****.***** **.***** ***

SGD_Scer_INP53/YOR109W     TCCGGTGTCAGAAAACTCATTCCATTAATATTGGACTCTCTTTTATCTTCTGGCTATTAT      1200
FM1318/1-3327              TCCGGTGTTAGGAAAGTTATTCCACTAATAATGGACTCTCTTCTTTCATCTGGCTATTAC      1200
6261_YOR109W               TCCGGTGTCAGAAAACTTATTCCATTAATATTGGACTCTCTTTTATCTTCTGGCTATTAT      258
                           ******** **.*** * ****** *****:*********** *:**:***********

SGD_Scer_INP53/YOR109W     TCTTACGATGTTAGAGAAAAAAAGAACATATCTGAACAACATGGCATATTTAGGACCAAC      1260
FM1318/1-3327              TCCTATGATGTCCGAGAAAGAAAAAATATATCCGAGCAACACGGTATATTTAGAACTAAT      1260
6261_YOR109W               TCTTACGATGTTAGAGAAAAAAAGAACATATCTGAACAACATGGCATATTTAGGACCAAC      318
                           ** ** *****  .******.***.** ***** **.***** ** ********.** **

SGD_Scer_INP53/YOR109W     TGTTTAGATTGTTTGGATAGAACAAATTTAGCTCAGCAAATTATTTCTTTGGCTGCTTTT      1320
FM1318/1-3327              TGTTTAGATTGTTTGGATAGGACAAATTTAGCTCAGCAGGTCATTTCGTTAGCTGCTTTA      1320
6261_YOR109W               TGTTTAGATTGTTTGGATAGAACAAATTTAGCTCAGCAAATTATTTCTTTGGCTGCTTTT      378
                           ********************.*****************..* ***** **.********:

SGD_Scer_INP53/YOR109W     AGAACTTTTCTCGAAGATTTCCGATTGATTAGTTCAAATTCGTTCATCGACGATGATGAT      1380
FM1318/1-3327              AGAACTTTTCTAGAAGATTTTAGATTGATTAGTTCAAACTCCTTCATTGATGACGATGAT      1380
6261_YOR109W               AGAACTTTTCTCGAAGATTTCCGATTGATTGGTTCAAATTCGTTCATCGACGATGATGAT      438
                           ***********.******** .*********.******* ** ***** ** ** ******

SGD_Scer_INP53/YOR109W     TTCGTTTCTAAACATAACACCCTGTGGGCTGATCACGGTGATCAAATTTCCCAAATATAT      1440
FM1318/1-3327              TTCGTTTCCAAGCACAATACCCTCTGGGCGGACCACGGTGATCAGGTCTCCCAAATATAT      1440
6261_YOR109W               TTCGTTTCTAAACATAACACCCTGTGGGCTGATCACGGTGATCAAATATCCCAAATATAT      498
                           ******** **.** ** ***** ***** ** ***********..* ************
```

```
SGD_Scer_INP53/YOR109W      ACTGGTACTAATGCTTTGAAGTCCTCATTTTCAAGAAAAGGTAAAATGTCACTTGCTGGG      1500
FM1318/1-3327               ACTGGTACCAATGCTTTAAAGTCTTCATTTTCAAGAAAGGGTAAGATGTCACTTGCCGGG      1500
6261_YOR109W                ACTGGTACTAATGCTTTGAAGTCCTCCTTTTCAAGAAAAGGTAAAATGTCACTTGCTGGG      558
                            ******** ********.***** **.***********.*****.*********** ***

SGD_Scer_INP53/YOR109W      GCATTATCAGACGCCACAAAATCGGTCAGCAGAATATATATTAACAATTTCATGGATAAA      1560
FM1318/1-3327               GCCTTATCAGACGCAACAAAGTCTGTCAGCAGAATATATATCAACAACTTTATGGATAAG      1560
6261_YOR109W                GCATTATCAGACGCCACAAAATCGGTCAGCAGAATATATATTAACAATTTCATGGATAAA      618
                            **.***********.*****.** ***************** ***** ** ********.

SGD_Scer_INP53/YOR109W      GAAAAGCAACAAAATATCGATACTTTGTTGGGAAGGTTACCGTATCAGAAAGCAGTGCAA      1620
FM1318/1-3327               GAAAAGCAGCAGAATATTGATACTTTGTTGGGAAGGTTACCATACCAGAAAGCAGTACAA      1620
6261_YOR109W                GAAAAGCAACAAAATATCGATACTTTGTTGGGAAGGTTACCGTATCAGAAAGCAGTGCAa      678
                            ********.**.***** **********************.** ***********.**

SGD_Scer_INP53/YOR109W      CTTTATGATCCCGTAAACGAATACGTAAGTACGAAATTACAAAGCATGTCTGATAAGTTC      1680
FM1318/1-3327               TTATATGATCCTGTGAATGAATACGTCAGCACCAAGCTACAGAGCATGTCTGATAAATTT      1680
6261_YOR109W                CTTTATGATCCCGTAnACGAATACGTAAGTACGAnATTACAAAGCATGTCTGATAAGTTC      738
                             *:******** **. * ********.** ** *  . ****.**************.**

SGD_Scer_INP53/YOR109W      ACATCAACCTCCAACATTAACTTGTTAATAGGATCATTCAATGTTAATGGCGCAACCAAA      1740
FM1318/1-3327               ACATCCTCCTCGAACATCAGCTTGCTAATAGGTTCTTATAATGTTAATGGGACCACCAAA      1740
6261_YOR109W                ACATCAACCTCCAACATTAACTTGTTAGTAGGATCATTCAATGTTAATGGAGcnnCCAAg      798
                            *****.:**** ***** *.**** **.****:**:*: ***********  .   ****

SGD_Scer_INP53/YOR109W      AAAGTTGATTTATCAAAGTGGTTATTTCCAATCGGTGAAAAATTTAAACCCGATATTGTT      1800
FM1318/1-3327               AAAGCAGATTTGTCAAAGTGGTTATTTCCGATAGGCGAAAAATTCAAACCAGACATCGTT      1800
6261_YOR109W                nnaGTTGATTTATCnnaGTGgttATTTCCAAT----------------------------      830
                               * :*****.**   ***   ******.**
```

RGA1/YOR127w, *Sc> Se* at 250-255bp

```
SGD_Scer_RGA1/YOR127W      -----------------------------------------------ATGGCATCAACTG      13
FM1318/1-3024              -----------------------------------------------ATGGCGTCAACCT      13
6261_YOR127W               AgGATAGCTGAttcAGGTACTAGTGGtGGAnngngcgGCAtaTTAAAATggcATCAACTg      60
                                                                          **   .*****

SGD_Scer_RGA1/YOR127W      CTCCCAATGAACAATTTCCATCCTGCGTACGATGCAAAGAATTTATTACCACGGGGCATG      73
FM1318/1-3024              TGCCCAACGAACAATTTCCCTCGTGTGTGCGGTGCAAAGATTCTATAACCACTGGGCACG      73
6261_YOR127W               nnCCCAATGaacaaTTTCcntCCTGCGTACGAtgcAAaGAATTtATTACCACGGGGCATG      120
                             ***** *     ****   * ** **.**.   ** **:*  **:***** ***** *

SGD_Scer_RGA1/YOR127W      CATATGAGTTGGGTTGTGATAGATGGCACACACATTGTTTCGCTTGTTACAAATGTGAGA      133
FM1318/1-3024              CGTATGAGCTAGGTTGTGATAGATGGCACACGCATTGTTTCTCATGCTATAAATGTGAGA      133
6261_YOR127W               CATaTGAGTTGGGTTGTGATAGATGGCACAcacATTGTTTCGCTTGTTACAAATGTGAGA      180
                           *.* **** *.*******************   ******** *:** ** **********

SGD_Scer_RGA1/YOR127W      AACCATTAAGCTGCGAATCTGATTTTTTAGTCCTTGGAACAGGTGCTTTGATCTGCTTTG      193
FM1318/1-3024              AACCATTAAGTTGTGAATCTGATTTTCTAGTACTTGGAACTGGTGCCTTAATTTGTTTCG      193
6261_YOR127W               AACCATTAAGCTGCGAATCTGAtTTTTTAGTCCTTGGAACAGGTGCTTTGATCTGCTTTG      240
                           ********** ** ******** *** ****.********:***** **.** ** ** *

SGD_Scer_RGA1/YOR127W      ATTGTTCCGATTCTTGTAAAAATTGCGGTAAAAAGATTGATGATTTGGCCATAATACTGT      253
FM1318/1-3024              ATTGTTCCGATTCATGCAAAAATTGTGGTAAGAAAATTGACGATCTGGCCATTATATTGT      253
6261_YOR127W               ATTGTTCCGATTCTTGTAAAAATTGCGGTAAAAAGATTGATGATTTGGCCATAATACTGT      300
                           *************:** ******** *****.**.***** *** *******:*** ***

SGD_Scer_RGA1/YOR127W      CCTCTTCAAATGAGGCCTATTGTTCAGATTGTTTTAAATGCTGTAAATGTGGTGAAAATA      313
FM1318/1-3024              CTTCGTCAAATGAAGCTTATTGTTCAGATTGTTTCAAATGTTGCAAGTGTGGTGATAATA      313
6261_YOR127W               CTTCGTCAAATGAAGCTTATTGTTCAGATTGTTTCAAATGTTGCAAGTGTGGTGATAATA      360
                           * ** ********.** ***************** ***** ** **.********:****
```

```
SGD_Scer_RGA1/YOR127W      TTGCTGACCTACGGTACGCGAAAACCAAGCGAGGTTTATTCTGTTTAAGCTGTCACGAAA     373
FM1318/1-3024              TTGCTGATTTGCGATACGCAAAGACCAAGCGGGGCTTATTCTGTTTAAATTGCCACGAGA     373
6261_YOR127W               TTGCTGATTTGCGATACGCAAAGACCAAGCGGGGCTTATTCTGTTTAAATTGCCACGAGA     420
                           *******  *.**.*****.**.********.** ************. ** *****.*

SGD_Scer_RGA1/YOR127W      AGCTATTAGCCAAACGAAAATACTACGAAGAGAAGAAAAGGCGACTCAAAAAGAATTTAC     433
FM1318/1-3024              AGCTATTAGCCAAAAGGAAATATTACGAGGAGAAAAAAAGACGACTTAAAAAAAATTTGC     433
6261_YOR127W               AGCTATTAGCCAAAAGGAAATATTACGAGGAGAAAAAAAGACGACTTaAAAAAAATTTGC     480
                           **************.*.***** *****.*****.*****.*****  ****.*****.*

SGD_Scer_RGA1/YOR127W      CAAGTTTACCCACACCTGTGATTGATAATGGCCATACTGATGAGGTCTCAGCTTCTGCAG     493
FM1318/1-3024              CCAGTCTTCCTACTCCCGTGCTTGACAATGATTCTATTGATGTAACTTCAATTACGGCAG     493
6261_YOR127W               CCAGTCTTCCTACTCCCGTGCTTGACAATGATTCTATTGATGTAACTTCAATTACGGCAG     540
                           *.*** *:** **:** ***.**** ****.   .** *****:..  ***. *:* ****

SGD_Scer_RGA1/YOR127W      TCCTCCCAGAAAAAACATTTAGCAGACCTGCATCACTAGTTAATGAGATTCCTTCAGGTT     553
FM1318/1-3024              TTGCACCCAAAAGGTCATCTAGTAGACCTGTATCACCGGTTAAGAAAATATCCTTAGAGT     553
6261_YOR127W               TTGCACCCAAAAGGTCATCTAGTAGACCTGTATCACCGGTTAAGAAAATATCCTTAGAGT     600
                           *   .**..***..:*** *** ******* ***** .***** .*.**: * * **. *

SGD_Scer_RGA1/YOR127W      CTGAACCTT------CCAAGGACATAGAAACCAATTCGAGTGATATTGTTCCGCATTTTA     607
FM1318/1-3024              CCGAATCTATGAAAGATATAGCAATAGAAACCAACTCGAGCGATATCATTCCGCATTTCA     613
6261_YOR127W               CCGAATCTATGAAAGATATAGCAATAGAAACCAACTCGAGCGATATCATTCCGCATTTCA     660
                           * *** **:        . *:.*..*********** ***** ***** .********* *

SGD_Scer_RGA1/YOR127W      TCACTGGGTATAACGATAGCGATGACAACTCTGGAAGTTCAAAATTCGGTTCAAATGTGT     667
FM1318/1-3024              TCACTGGGTATGATGATAGCGACGATAATTCTGGAAGTTCGAAATTCGGTTCTAATATTT     673
6261_YOR127W               TCACTGGGTATGATGATAGCGACGATAATTCTGGAAGTTCGAAATTCGGTTCTAATATTT     720
                           ***********.* ******** ** ** ***********.***********:***.* *

SGD_Scer_RGA1/YOR127W      CCATAGATGTTATAGGACCGGAAGAAAATAGCACGGAGCATGTAAATGATGATGTTAAAG     727
FM1318/1-3024              CAATAGACATTATAGAACCACAGCAAGATAGCGCGGAGCATGCAAAAGATGAGAAAGTAG     733
6261_YOR127W               CAATAGACATTATAGAACCACAGCAAGATAGCGCGGAGCATGCAAAAGATGAGAAAGTAG     780
                           *.***** .******.***. *. **.*****.********* ***:***** .::.:**

SGD_Scer_RGA1/YOR127W      AGGAAGCAGAAGCACCTTCAGCGAATATGTCACTCAATGTTGCTACGGATCCA-------     780
FM1318/1-3024              AAGAGGTGAAGGTGCATTCTAGNNNNNNNNNNNNNNNNNNNNNNATTGGATGCTACTCCAA     793
6261_YOR127W               AAGAGGTGAAGGTGCATTCTAGAAACGCATCGCTTGACATAGCATTGGATGCTACTCCAA     840
                           *.**.* ..*.* .*.***:.          .   .     .   :: **** *:

SGD_Scer_RGA1/YOR127W      -----------ACCCTAAGTTGTAAAGAACCTCCTAGCCATTCGAGGAATTTGTTAAATA     829
FM1318/1-3024              GTCATAAGGTCTTGTTGGGCAACACGGAACCTCCGAGCCGCTCTAAGATTTTATTAAACA     853
6261_YOR127W               GTCATAAGGTCTTGTTGGGCAACACGGAACctCCGAGCCGCTCTAAGATTTTATTAAACA     900
                                      :   *..* :. *..****  ** ****. ** *.**:***.***** *

SGD_Scer_RGA1/YOR127W      AAACACCGTTGAGAAATTCTTCGGGTCAGTATCTCGCAAAATCTCCAAGCTCCTATAGAC     889
FM1318/1-3024              AAACACCATTAAGGAATTCATCCGGACAATATGTCGCAAAATCTCCAAGTTCTTATAGAC     913
6261_YOR127W               AAACACCATTAAGGAATTCATCCGGACAATATGTCGCAAAATCTCCAAGTTCTTATAGAC     960
                           *******.**.**.*****:** **:**.*** **************** ** *******

SGD_Scer_RGA1/YOR127W      AGGGAATAATTGTTAACGATAGTCTGGAAGAGAGCGATCAAATTGATCCTCCAAATAACA     949
FM1318/1-3024              AGGGTATAGTTGTTAATGATAGCTTTGAGGAAAATAACCAGGTCGAACCTCCAANNGACG     973
6261_YOR127W               AGGGTATAGTTGTTAATGATAGCTTtGAGGAAAaTAACCAGGTCGAACctCCAAAtGACG     1020
                           ****:***.******* *****  * **.**.*  .* **..* **:*  ****  .**.

SGD_Scer_RGA1/YOR127W      GTTCACGAAATGCAAGTGAATTGTTGACCTCGGTATTGCATAGCCCAGTTTCTGTTAATA     1009
FM1318/1-3024              GTTCCCGAACCGCAAGTGAGCTGTTGAGCTCGGTATTACATAGCCCTGTTTCTGTTAATA     1033
6261_YOR127W               gtCCCGAACCGCAA----------------------------------------------     1034
                              *. .*..  .*
```

HSP82/YPL240c, Se>*Sc* at 1578-1608bp *

```
SGD_Scer_HSP82/YPL240C      TGGTTATCTTTCGTCAAGGGTGTTGTTGACTCTGAGGATTTACCATTGAATTTGTCCAGA      1140
MIT_Sbay_c60_24336          ---ATGTCATTCGTCAAGGGTGTTGTTGACTCCGAAGATTTACCATTGAACTTGTCCAGA      57
6261_YPL240C                TGGATGTCCTTTGTCAAGGGTGTTGTTGACTCTGAAGACTTACCATTGAACCTGTCCAGA      123
                               :*.** ** ******************* **.** ***********  ********

SGD_Scer_HSP82/YPL240C      GAAATGTTACAACAAAATAAGATCATGAAGGTTATTAGAAAGAACATTGTCAAAAAGTTG      1200
MIT_Sbay_c60_24336          GAAATGTTGCAACAAAACAAGATCATGAAGGTCATCAGAAAGAACATCGTTAAGAAGATG      117
6261_YPL240C                GAAATGCTACAACAAAACAAGATCATGAAGGTTATCAGAAAGAACATCGTCAAGAAGGTA      183
                            ****** *.******** ************** ** *********** ** **.*** *.
```

```
SGD_Scer_HSP82/YPL240C      ATTGAAGCCTTCAACGAAATTGCTGAAGACTCTGAACAATTTGAAAAGTTCTACTCGGCT      1260
MIT_Sbay_c60_24336          ATTGAATCCTTCAACGAAATCGCTGAAGACTCTGAACAATTCGAAAAGTTCTACTCTGCC      177
6261_YPL240C                ATTGAAGCTTTCAACGAAATTGCTGAAGACTCTGAACAATTCGAAAAGTTCTACTCTGCC      243
                            ****** * *********** ******************** ************** **

SGD_Scer_HSP82/YPL240C      TTCTCCAAAAATATCAAGTTGGGTGTACATGAAGATACCCAAAACAGGGCTGCTTTGGCT      1320
MIT_Sbay_c60_24336          TTCGCTAAGAACATCAAATTGGGTGTTCATGAAGACACTCAAAACAGAGCTGCCTTGGCC      237
6261_YPL240C                TTCGCTAAGAACATCAAATTGGGTGTCCACGAAGACACTCAAAACAGAGCTGCCTTGGCT      303
                            *** * **.** *****.******** ** ***** ** ********.***** *****

SGD_Scer_HSP82/YPL240C      AAGTTGTTACGTTACAACTCTACCAAGTCCGTAGATGAGTTGACTTCCTTAACTGATTAC      1380
MIT_Sbay_c60_24336          AAATTGCTACGTTACAACTCCACCAAGTCCGTTGACGAATTGACTTCTTTGACTGATTAT      297
6261_YPL240C                AAGTTACTACGTTACAACTCCACCAAGTCCGTCGACGAATTAACTTCTTTGACCGATTAC      363
                            **.**. ************* *********** ** **.**.***** **.** *****

SGD_Scer_HSP82/YPL240C      GTTACCAGAATGCCAGAACACCAAAAGAACATCTACTACATCACTGGTGAATCTCTAAAG      1440
MIT_Sbay_c60_24336          ATTACCAGAATGCCAGAACACCAAAAGAACATCTACTATATCACAGGTGAGTCTTTGAAG      357
6261_YPL240C                ATCACCAGAATGCCAGAACACCAAAAGAACATTTACTACATCACAGGTGAATCTCTAAAG      423
                            .* ***************************** ***** *****:*****.*** *.***

SGD_Scer_HSP82/YPL240C      GCTGTCGAAAAGTCTCCATTTTTGGATGCCTTGAAGGCTAAAAACTTCGAGGTTTTGTTC      1500
MIT_Sbay_c60_24336          GCTGTTGAAAAATCCCCATTCTTAGACGCTTTGAAAGCTAAGAACTTTGAAGTTTTGTTC      417
6261_YPL240C                GCCGTTGAAAAATCCCCATTCTTGGACGCTTTGAAGGCTAAGAACTTTGAAGTTTTGTTC      483
                            ** ** *****.** ***** **.** ** *****.*****.***** **.*********

SGD_Scer_HSP82/YPL240C      TTGACCGACCCAATTGATGAATACGCCTTCACTCAATTGAAGGAATTCGAAGGTAAAACT      1560
MIT_Sbay_c60_24336          TTGACTGATCCAATCGATGAATACGCCTTCACTCAATTAAAGGAATTCGAAGGTAAGACT      477
6261_YPL240C                TTGACTGATCCAATCGATGAATACGCTTTCACCCAATTGAAGGAATTCGAAGGTAAGACT      543
                            ***** ** ***** *********** ***** *****.*****************.***

SGD_Scer_HSP82/YPL240C      TTGGTTGACATTACTAAAGATTTCGAATTGGAAGAAACTGACGAAGAAAAAGCTGAAAGA      1620
MIT_Sbay_c60_24336          TTAGTCGATATCACCAAGGATTTCGAGCTGGAAGAAACTGACGAAGAGAAAGCTGAAAGA      537
6261_YPL240C                TTGGTCGATATCACCAAGGATTTCGAACTGGAAGAAACTGACGAAGAAAAAGCTGAAAGA      603
                            **.** ** ** ** **.********. *******************.************

SGD_Scer_HSP82/YPL240C      GAGAAGGAGATCAAAGAATATGAACCATTGACCAAGGCCTTGAAAGAAATTTTGGGTGAC      1680
MIT_Sbay_c60_24336          GAAAAGGAAGTTAAAGAATTCGAACCATTGACCAAGGCCTTGAAAGACATCTTGGGTGAA      597
6261_YPL240C                GAGAAGGAGATCAAAGAATATGAACCATTGACCAAGGCCTTGAAAGAAATTTTGGGTGAC      663
                            **.*****..* *******: **************************.** ********.

SGD_Scer_HSP82/YPL240C      CAAGTGGAGAAAGTTGTTGTTTCTTACAAATTGTTGGATGCCCCAGCTGCTATCAGAACT      1740
MIT_Sbay_c60_24336          CAAGTTGAAAAGGTTGTTGTCTCTTACAAACTAGTGGATGCCCCAGCTGCCATTAGAACT      657
6261_YPL240C                CAAGTGGAGAAAGTTGTTGTTTCTTACAAATTGTTGGATGCCCCAGCTGCTATCAGAACT      723
                            ***** **.**.******** ********* *. **************** ** ******

SGD_Scer_HSP82/YPL240C      GGTCAATTTGGTTGGTCTGCTAACATGGAAAGAATCATGAAGGCTCAAGCCTTGAGAGAC      1800
MIT_Sbay_c60_24336          GGCCAATTCGGTTGGTCCGCTAACATGGAAAGAATCATGAAGGCTCAAGCTTTGAGAGAC      717
6261_YPL240C                GGTCAATTTGGTTGGTCTGCTAACATGGAAAGAATCATGAAGGCTCAAGCCTTGAGAGAC      783
                            ** ***** ******** ******************************** *********

SGD_Scer_HSP82/YPL240C      TCTTCCATGTCCTCCTACATGTCTTCCAAGAAGACTTTCGAAATTTCTCCAAAATCTCCA      1860
MIT_Sbay_c60_24336          TCTTCCATGTCCTCTTACATGTCCTCCAAGAAGACTTTCGAAATCTCTCCAAAATCTCCA      777
6261_YPL240C                TCTTCCATGTCCTCCTACATGTCTTCCAAGAAGACTTTCGAAATTTCTCCAAAATCTCCA      843
                            ************** ******** ******************** ***************

SGD_Scer_HSP82/YPL240C      ATTATCAAGGAATTGAAAAAGAGAGTTGACGAAGGTGGTGCTCAAGACAAGACTGTCAAG      1920
MIT_Sbay_c60_24336          ATTATCAAGGAATTGAAAAAGAGAGTTGATGAAGGCGGTGCTCAAGATAAGACTGTCAAG      837
6261_YPL240C                ATTATCAAGGAATTGAAAAAGAGAGTTGACGAAGGT------------------------      879
                            ***************************** *****
```

DBVPG 6257

YDR338c, *Se> Sc* at 715-723bp

```
SGD_Scer_YDR338C      GATGAGGAACCTGACCTTTACTATCATGATGAAGAAGATGGCGAACTGAGCAGAACGATT  300
FM1318/1-2088         GAAGAAGAACCTGATTTATATTACCACGACGAGGAGGATGGTGAGCTTAGTAGAACCATT  300
6257_YDR338C          ---------cCTGATTTATATTACCACGACGAGGAGGATGGCGAGCTTAGTAGAACCATT  51
                                ****  *:** ** ** ** **.**.***** **.** ** ***** ***

SGD_Scer_YDR338C      TCTCTACCATCAAGGGTATCAGAAACTCCTGAGCTTTCGCCACAAGATGTTGATTGGATT  360
FM1318/1-2088         TCTTTACCTTCAAGAGTGTCAGAGACTCCTGAACTTTCTCCACAAGACGTTGACTGGATA  360
6257_YDR338C          TCTTTACCTTCAAGAGTGTCAGAGACTCCTGAACTTTCTCCACAAGACGTTGACTGGATA  111
                      *** ****:*****.**.*****.********.***** ******** ***** *****:

SGD_Scer_YDR338C      CTACATGAACATGAACGACGATACTCATCGGTATGCAACTCTGATAATGAGGAAGCAAGT  420
FM1318/1-2088         CTACATGAACATGAACGACGATACTCGTCAGTGTATAACTCTGATAATGAGGAGGAGTGT  420
6257_YDR338C          CTACATGAACATGAACGACGATACTCGTCAGTGTATAACTCTGATAATGAGGAGGAGTGT  171
                      **************************.**.**.*. *****************.*..:**

SGD_Scer_YDR338C      CAAAGCAACACACCAGATAGAATACAAGAATATTCTGGAAGAGAACTAGAATATGACGAA  480
FM1318/1-2088         CAAAGCAACATACCCGATAGAACACAAGGATATCCCGGAAGAGAACTGGAATATGACGAA  480
6257_YDR338C          CAAAGCAACATACCCGATAGAACACAAGGATATCCCGGAAGAGAACTGGAATATGACGAA  231
                      ********** ***.******* *****.**** * ***********.************

SGD_Scer_YDR338C      TTTATGAATAGACTCCAAGCTCAGAAACAGAAATTAACTCGAAGTGCGGTAACAGACGCT  540
FM1318/1-2088         TTTATGAACAGGCTTCAAGCCCAGAAACAGAAACAAAACCAAAATGCAACAATGGATGCA  540
6257_YDR338C          TTTATGAACAGGCTTCAAGCCCAGAAACAGAAACAAAACCAAAATGCAACAATGGATGCA  291
                      ******** **.** ***** ************ :**. *.**.***.. ** .** **:

SGD_Scer_YDR338C      AAAGGCACCTCACATCACAGAAGACCATCATTCGTATCTGTGACTAGCCGAGGCTCAGTT  600
FM1318/1-2088         AAAGGTATCTCACATCGTAGAAGGCCATCATTCGTATCTGTAAACAGTCGAGGTTCGGTG  600
6257_YDR338C          AAAGGTATCTCACATCGTAGAAGGCCATCATTCGTATCTGTAAACAGTCGAGGTTCGGTG  351
                      ***** * ********. *****.****************.*.  ** ***** **.**

SGD_Scer_YDR338C      CCCACAATTTACCAAGAGATCGACGAGAACGATTCTGAGGCACTAGCAGAATTGGCTCAC  660
FM1318/1-2088         CCCACGATATATCAAGACCTAGATGAGAATGATCCAGAAGCACTAGCTGCATTGGCTCAT  660
6257_YDR338C          CCCACGATATATCAAGACCTAGATGAGAATGATCCAGAAGCACTAGCTGCATTGGCTCAT  411
                      *****.**:** ***** .*.** ***** *** *:**.********:*.*********

SGD_Scer_YDR338C      AGCCACGTCACCTTCAAATCAGAAGCAAGAGTTTTGGCATCTTATTCCTTCCCTCTAATT  720
FM1318/1-2088         AGTAATGTGACCTTCAAATCAGAAGCAAAAGTATTAGCATCTTACTCTTTCCCTTTAATT  720
6257_YDR338C          AGTAATGTGACCTTCAAATCAGAAGCAAAAGTATTAGCATCTTACTCTTTCCCTTTAATT  471
                      ** .* ** *******************.***:**.******** ** ****** *****

SGD_Scer_YDR338C      TTCACATTCTTATTGGAACAGATTTTCCCTATGGTATGTTCATTAACTGTGGGCCACTTA  780
FM1318/1-2088         TTTACATTTTTACTGGAACAAATTTTCCCTATGGTTTGTTCATTAACCGTGGGCCATCTA  780
6257_YDR338C          TTCACATTCTTATTGGAACagaTTTTCCCTATGGTATGTTCATTAACTGTGGGCCActtA  531
                      ** ***** *** ******   *************:*********** ********   *

SGD_Scer_YDR338C      GGCAAAAATGAACTAGCAGCTGTGTCCTTAGCATCCATGACTTCTAATATAACACTAGCG  840
FM1318/1-2088         GGTAAGAATGAGCTAGCTGCTGTATCGTTAGCTTCTATGACTTCCAATATCACATTAGCC  840
6257_YDR338C          GgCAAAAATGAACTAGcagCTGTGTCCTTAGcatCCATGACTTCTAATATAaCactAGcg  591
                      *  **.*****.****   ****.** ****   * ******** *****. *   **

SGD_Scer_YDR338C      ATATTCGAGGGTATTGCCACTAGTCTAGATACTCTATGCCCTCAAGCGTATGGCTCTGGG  900
FM1318/1-2088         ATATTTGAAGGTATTGCCACTAGTCTCGATACTCTTTGTCCTCAAGCATACGGTTCTGGA  900
6257_YDR338C          aTATTcgaggGTATTGCCACTAGTCTagATACTCTATGCCCTCAAGCGTATGGCTCTGgn  651
                       ****     ****************  *******:** ********.** ** ****

SGD_Scer_YDR338C      AGATTCTACAGTGTAGGAGTTCACCTTCAACGTTGCATTGCTTTTTCATTGGTCATATAT  960
FM1318/1-2088         AGGTTCTACAGTGTCGGGGTTCATTTTCAACGTTGTATTGCTTTTTCGATGGTCATATAT  960
6257_YDR338C          nnntTCTACAGTGtagnagTTCacctTCAACGTTGCATTGCTTTTtcnnnggTCATATAt  711
                          *********      ***    ********* *********       *******

SGD_Scer_YDR338C      ATACCTTTTGCTGTCATGTGGTGGTATTCTGAACCTCTTCTTTCTTATATCATCCCTGAG  1020
FM1318/1-2088         GTTCCGTTTGCGTTCTTGTGGTGGTATTCTGAGCCTCTTCTTTCTTATATCATTCCTGAA  1020
6257_YDR338C          ataccTTTTGCTGTCAtGt-----------------------------------------  730
                            *****  **: *
```

KEM1/YGL173c, *Se> Sc* at 462-477bp

```
SGD_Scer_XRN1/YGL173C      --------------ATGGGTATTCCAAAATTTTTCAGGTACATCTCAGAAAGATGGCCCA      46
FM1318/1-4548              --------------ATGGGTATTCCAAAATTTTTCAGGTACATCTCAGAAAGATGGCCCA      46
6257_YGL173C               TATACTAGTACAGCATGGGTATTCCGAAATTTTTCAGGTACATCTCAGAAAGATGGCCCA      360
                                         ***********.**********************************

SGD_Scer_XRN1/YGL173C      TGATTTTACAGCTTATTGAGGGAACACAGATTCCTGAGTTTGATAACTTATACCTGGATA      106
FM1318/1-4548              TGATTCTACAACTTATTGAAGGGACTCAGATTCCCGAGTTTGATAACCTATATCTGGATA      106
6257_YGL173C               TGATTCTACAACTTATTGAAGGGACTCAGATTCCTGAGTTTGATAACCTATATCTGGATA      420
                           ***** ****.********.**.**:******** ************ **** *******

SGD_Scer_XRN1/YGL173C      TGAATTCGATTTTACATAATTGTACGCATGGTAACGACGATGATGTAACCAAGCGATTAA      166
FM1318/1-4548              TGAATTCGATTTTACATAACTGTACACATGGTAACGACGATGACGTGACGAAGCGATTAA      166
6257_YGL173C               TGAATTCGATTTTACATAACTGTACACATGGTAACGACGATGACGTGACGAAGCGATTAA      480
                           ******************* *****.***************** **.** **********

SGD_Scer_XRN1/YGL173C      CTGAAGAAGAGGTTTTTGCAAAAATCTGTACGTATATCGATCACCTTTTTCAAACAATCA      226
FM1318/1-4548              CTGAAGAAGAGGTTTTTGCAAAGATCTGCACGTATATTGATCATCTTTTTCAAACTATCA      226
6257_YGL173C               CTGAAGAAGAGGTTTTTGCAAAGATCTGCACGTATATTGATCATCTTTTTCAAACTATCA      540
                           **********************.***** ******** ***** ***********:****

SGD_Scer_XRN1/YGL173C      AGCCCAAGAAGATTTTCTACATGGCTATTGATGGTGTGGCCCCTCGTGCCAAGATGAATC      286
FM1318/1-4548              AGCCCAAGCAAATTTTTTACATGGCTATCGATGGTGTGGCCCCCCGTGCGAAGATGAATC      286
6257_YGL173C               AGCCCAAGCAAATTTTTTACATGGCTATCGATGGTGTGGCCCCCCGCGCGAAGATGAATC      600
                           ********.*.***** *********** ************** ** ** **********

SGD_Scer_XRN1/YGL173C      AACAAAGAGCTCGTAGATTCAGAACCGCTATGGATGCAGAAAAAGCCTTGAAGAAGGCTA      346
FM1318/1-4548              AACAGAGAGCTCGTAGATTTAGAACTGCCATGGACGCCGAAAAAGCTATGAAAAAAGCTA      346
6257_YGL173C               AACAGAGAGCTCGTAGATTTAGAACTGCCATGGACGCCGAAAAAGCTATGAAAAAAGCTA      660
                           ****.************** ***** ** ***** **.******** :****.**.****

SGD_Scer_XRN1/YGL173C      TTGAGAATGGTGACGAGATTCCTAAGGGTGAGCCATTTGATTCGAATTCTATTACTCCAG      406
FM1318/1-4548              TTGAAAATGGTGACGAAATTCCTAAAGGTGAGCCGTTCGACTCAAACTGTATTACTCCAG      406
6257_YGL173C               TTGAAAATGGTGACGAAATTCCTAAAGGTGAGCCGTTCGACTCAAACTGTATTACTCCAG      720
                           ****.***********.********.********.** ** **.** * ***********

SGD_Scer_XRN1/YGL173C      GTACGGAGTTTATGGCCAAATTGACCAAAAACTTACAATATTTTATTCACGACAAGATTT      466
FM1318/1-4548              GTACTGAATTTATGGCTAAACTGACAAAAAAATTTACAGTATTTCATTCATGACAAAATTT      466
6257_YGL173C               GTACTGAATTTATGGCTAAACTGACAAAAAAATTTACAGTATTTCATTCATGACAAAATTT      780
                           **** **.******** *** ****.***** *****.***** ***** *****.****

SGD_Scer_XRN1/YGL173C      CTAACGATTCCAAATGGAGGGAAGTGCAAATCATATTTTCTGGCCATGAAGTTCCAGGTG      526
FM1318/1-4548              CTAACGATTCAAAGTGGAGAGAAGTGCAAATCATATTTTCTGGCCATGAAGTTCCAGGTG      526
6257_YGL173C               CTAACGATTCCAAATGGAGGGAAGTGCAAATCATATTTTCTGGCCATGAAGTTCCAGGTG      840
                           **********.**.*****.****************************************

SGD_Scer_XRN1/YGL173C      AAGGTGAACACAAGATCATGAACTTTATAAGGCATTTAAAATCCCAAAAGGATTTCAACC      586
FM1318/1-4548              AAGGTGAGCACAAAATTATGAATTTCATAAGGCATTTAAAATCCCAGAAGGATTTCAACC      586
6257_YGL173C               AAGGTGAACACAAGATCATGAACTTTATAaGgCaTTTAAAATCCCAAAAGGATTTCAACC      900
                           *******.*****.** ***** ** *** * * ************.*************

SGD_Scer_XRN1/YGL173C      AGAATACGAGACATTGTATTTACGGTCTTGACGCAGATTTGATTATGCTGGGTTTGTCTA      646
FM1318/1-4548              AAAATACGAGACACTGTATTTATGGTCTGGATGCGGATTTGATTATGCTTGGTTTATCTA      646
6257_YGL173C               AGAATACGAGACAtnGtntTAC--------------------------------------      922
                           *.***********  *   *:.
```

IRR1/YIL026c, *Se> Sc* at 1551-1571bp

```
SGD_Scer_IRR1/YIL026C    TATCTTTTCCAGGACTATTTGACCCAACAGGCTGTTAATTTAGAAAAGAATTATTTGGCG    954
FM1318/1-3447            TTTCTTTTCCAAGACTACTTGACTGAACAAGCTGTAAACTTGGAGAAGAATTACTTAGCC    945
6257_YIL026C             -------------------------------------------------------TAGCC    5
                                                                                *.**

SGD_Scer_IRR1/YIL026C    AAGTTATCCAAGCAGTTATCACTAGAAGAGAAGAAAAAAAGGCCCAATAACAAGACTTTA    1014
FM1318/1-3447            AAATTGACTAGACAGTTGTCACTAGAAGAAAAGAAAAAAAGGCCCAATAAGAAAACTTTA    1005
6257_YIL026C             AAATTGACTAGACAGTtGTCACTAGAagAAAagAAAAAAAggCCCAatAAGAAAACTTTA    65
                         **.**.:* *..**** .********  *.*  *******  ****  ** **.******

SGD_Scer_IRR1/YIL026C    GAAAAGCTAGAGAGCACTATCGCCGAAACTCAAGGCAGCAAAGTCGTTATTGATAGCATT    1074
FM1318/1-3447            GAAAAGCTCGAAAGCACCATTGCTGAAACTCAAGGTAGTAAAGTTGTCATTGAAGGTGTT    1065
6257_YIL026C             GAAAAGCtcGAaAGCACcATTGCTGAAACtCAAGGTAGTAAAGTTGTCATTGAAGGTGTT    125
                         *******  ** ***** ** ** ***** ***** ** ***** ** *****:.* .**

SGD_Scer_IRR1/YIL026C    ATCGATAACATCGTCAAACTATGTTTTGTGCATAGGTATAAGGACGTGTCTGATTTGATT    1134
FM1318/1-3447            ATTGATAATATTGTGAAGTTGTGTTTCGTGCACAGGTACAAGGACATATCCGATTCAATT    1125
6257_YIL026C             ATTGATAATAttgTGAAGTTGTGTTTCGTGCACAGGTACAagGACAtntCCGATTCAATT    185
                         ** ***** *   * **. *.***** ***** ***** *  ***.   * **** .***

SGD_Scer_IRR1/YIL026C    CGTTCAGAATCCATGTTGCATCTATCCATCTGGATTAAAAACTATCCCGAATATTTCCTC    1194
FM1318/1-3447            CGTTCTGAGTCCATGTTGCACTTATCGATCTGGATTAAAAACTATCCAGAATATTTCCTC    1185
6257_YIL026C             CGTTCTGAGTCCATGTTGCACTtaTCGATCTGGATTAAaAACTATCCAGAATATTTCCTC    245
                         *****:**.***********    ** *********** ********.************

SGD_Scer_IRR1/YIL026C    AAGGTTACATTTTTAAAATATTTTGGCTGGCTGCTGAGCGATAACTCTGTATCAGTCAGA    1254
FM1318/1-3447            AAGGTCACATTTTTGAAATATTTTGGCTGGCTACTCAGCGATAATTCCGTCTCTGTCAGG    1245
6257_YIL026C             AAGGTCACATnTnTGAAATATTTTGGCTGGCTACTCAGCGATAATTCCGTCTCTGTCAGG    305
                         ***** **** * *.*****************.** ******** ** **.**:*****.

SGD_Scer_IRR1/YIL026C    TTACAAGTTACGAAGATTTTACCGCATTTAATAATCCAAAATCATAACAGTAAATCCACT    1314
FM1318/1-3447            TTACAAGTTGCCAAGATTCTTCCACATTTGATCATCCAAAATCATAACAGCAAATCCAGT    1305
6257_YIL026C             TTACAAGTTGCCAAGATTCTTCCACATTTGATCATCCAAAATCATAACAGCAAATCCAGT    365
                         *********.* ****** *:**.*****.**.***************** ******* *

SGD_Scer_IRR1/YIL026C    GATAATTCCGCTATACGCCAAGTATTTGAACGCTTCAAAACTAAGATCCTGGAAGTGGCA    1374
FM1318/1-3447            GATAATTCCGCGATCCGCCAAGTCTTTGAAAGGTTTAAATCTAAGATCCTGGAGGTCGCC    1365
6257_YIL026C             GATAATTCCGCGATCCGCCAAGTCTTTGAAAGGTTTAAATCTAAGATCCTGGAGGTCGCC    425
                         *********** **.********.******.* ** ***:*************.** **.

SGD_Scer_IRR1/YIL026C    ATCCGTGACGTTAATCTTGATGTAAGGATCCATAGTATCCAAGTTCTAACGGAGGCGTCA    1434
FM1318/1-3447            ATCCATGACGTCAATCTTGATGTCAGAATTCATAGTGTTCAAATTCTAACTGAAGCGTCA    1425
6257_YIL026C             ATCCATGACGTCAATCTTGATGTCAGAATTCATAGTGTTCAAATTCTAACTGAAGCGTCA    485
                         ****.****** ***********.**.** ******.* ***.******* **.******

SGD_Scer_IRR1/YIL026C    TCATTGGGCTATTTAGATGATTCTGAGATTCTTATCATTTCTAGTTTAATGTTCGATGAA    1494
FM1318/1-3447            TCACTGGGCTATTTGGATGATTTTGAGATTCTAATGATTTCTAGCTTAATGTTTGATGAA    1485
6257_YIL026C             TCACTGGGCTATTTGGATGATTTTGAGATTCTAATGATTTCTAGCTTAATGTTTGATGAA    545
                         *** **********.******* *********:** ******** ******** ******

SGD_Scer_IRR1/YIL026C    GAGTTCGACCCATTTAAAACATCCTCATTCAATAAAAGATCCAAATTTCTATCCACGGTG    1554
FM1318/1-3447            GAATTCGACCCCTTCAAAACATCCTCATTCAATAAAAGGTCCAAATTTTTATCTACTGTA    1545
6257_YIL026C             GAATTCGACCCTTTCAAAACATCCTCATTCAATAAAAGGTCCAAATTTTTATCTACTGTA    605
                         **.******** ** ***********************.********* **** ** **.

SGD_Scer_IRR1/YIL026C    GCCAAATTCTTAGCAAGAGTAATAAAAGAGAAATTTGACGAATTCATCAAGACGCATGAA    1614
FM1318/1-3447            GCCAAGTTCTTAGCAAGAGTAATAAGCGAAAAACATGAGGAATTCACCAAGACCCATGAA    1605
6257_YIL026C             GCCAAGTTCTTAGCAAGAGTAATAAAAGAGAAATTTGACGAATTCATCAAGACGCATGAA    665
                         *****.*******************..**.*** :*** ******* ****** ******

SGD_Scer_IRR1/YIL026C    GACTTGCCCAAAGAAGTCGACGGGTTAGAAGTTGGACCCGTTGTTCAAGTCGGTATATTT    1674
FM1318/1-3447            GAGTTGCCTGAAGAAGTCGATGGATTAGCTGTTGCTCCAGTCGTGCAAGTTGGGATCTTT    1665
6257_YIL026C             GACTTGCCCAAAGAAGTCGACGGGTTAGAAGTTGGACCCGTTGTTCAAGTCGGTATATTT    725
                         ** ***** .********** **.****.:**** :**.** ** ***** ** **.***

SGD_Scer_IRR1/YIL026C    ATCAAGATTCTGAATGACTCTTTAATTTATCACTTGAAGGATTGCGCTGAAGTTGATTCA    1734
FM1318/1-3447            ATCAAGATCTTGAGCAACTCCTTGATTTATCATTTAAAAGATTCGGCGGAAGTTGATTCA    1725
6257_YIL026C             ATCAAGATTCTGAATGACTCTTTAATTTATCACTTGAAGGATTGCGCTGAAGTTGATTCA    785
                         ********  ***. .**** **.******** **.**.****  ** ************

SGD_Scer_IRR1/YIL026C    AGGACAAAGATCCGTATGCTAACACAAGCAGCAGAGTTTTTGTCTCCTTACATTTCCACT    1794
FM1318/1-3447            AAAACTAAGATTCGTATGCTAACGCAGGCCGCCGAGTTTTTATCTCCATACATTTCATCA    1785
6257_YIL026C             AGGACAAAGATCCGTATGCTAACACAAGCAGCAGAGTTTTTGTCTCCTTACATTTCCACT    845
                         *..**:***** ***********.**.**.**.********.*****:********.:*:
```

```
SGD_Scer_IRR1/YIL026C      CACTTGAAAACTATTTGCAATCTGCTGATCTCTGACACTGAATCAAATGAACTGATCCAA      1854
FM1318/1-3447              CACTTGAAAACTATCTGTGATCTTTTGATCTCGGATACTGAATCGAACGAATTGATCCAG      1845
6257_YIL026C               CACTTGAAAACTATTTGCAATCTGCTGATCTCTGACACTGAATCAAATGAACTGATCCAA      905
                           ************** ** .****  ******* ** ********.** *** *******.

SGD_Scer_IRR1/YIL026C      AAGTTACAAAACTCGGCCAATAATAACAGCGATGACGAGGATGTTGACGATGAGGAATTG      1914
FM1318/1-3447              ACACTACAAAACTCGACTGACAATAACAACGATGACGACGAGGAAGATGGTCAAGAACTA      1905
6257_YIL026C               AAGTTACAAAACTCGGCCAATAATAACAGCGATGACGATGATGTTGACGATGAGGAAtTg      965
                           *.. ***********.* .* *******.********* ** *::** *.* *.*** *

SGD_Scer_IRR1/YIL026C      GACATCACTCCATTGTTTCCCATTGACAGAAATAGCACTATTTTATATCTGAACGTATTC      1974
FM1318/1-3447              GACATCACTCCGCTGTTTCCCATTGACAGAAACAGCACTATATTATATCTTAACGTATTC      1965
6257_YIL026C               gACatcActCCAtGtTCCC-----------------------------------------      984
                            **   *  **.   *  *
```

TDH2/YJR009c, *Se> Sc* at 714-735bp*

```
SGD_Scer_TDH2/YJR009C      AAGGAATTGGACACTGCTCAAAAGCACATTGACGCTGGTGCCAAGAAGGTTGTCATCACT      360
WashU_Sbay_Contig534.6     AAGGAATTGGACACTGCTCAAAAGCACATTGACGCTGGTGCCAAGAAGGTTGTCATCACT      312
6257_YJR009C               -----------------------GCACATTGACGCTGgtGCCAAGAAGGTTGTCATCACT      37
                                                  **************  *********************

SGD_Scer_TDH2/YJR009C      GCTCCATCTTCCACCGCCCCAATGTTCGTCATGGGTGTTAACGAAGAAAAATACACTTCT      420
WashU_Sbay_Contig534.6     GCTCCATCTTCCACCGCCCCAATGTTCGTTATGGGTGTTAACGAAGACAAATACACTTCT      372
6257_YJR009C               GCTCCATCTTCCACCGccCCAATGTTCGTTATGgGTGTTAACGAAGACAAATACACTTCT      97
                           ****************  *********** *** *************.************

SGD_Scer_TDH2/YJR009C      GACTTGAAGATTGTTTCCAACGCTTCTTGTACCACCAACTGTTTGGCTCCATTGGCCAAG      480
WashU_Sbay_Contig534.6     GACTTGAAGATTGTCTCCAACGCTTCCTGTACCACTAACTGTTTGGCTCCATTGGCCAAG      432
6257_YJR009C               GACTTGAAGATTGtCTCCAACGCTTCCTGTACCACTAACTGTTtgGCTCCATTGGCCAAG      157
                           *************  *********** ******** *******  ***************

SGD_Scer_TDH2/YJR009C      GTTATCAACGATGCTTTCGGTATTGAAGAAGGTTTGATGACCACTGTTCACTCCATGACC      540
WashU_Sbay_Contig534.6     GTTATCAACGATGCTTTCGGTATTGAAGAAGGTTTGATGACCACTGTCCACTCCATGACC      492
6257_YJR009C               GTTATCAACGATGCTTTCGGTATTGAAGAAGGTTTGATGACCACTGTCCACTCCATGACC      217
                           *********************************************** ************

SGD_Scer_TDH2/YJR009C      GCCACCCAAAAGACTGTTGACGGTCCATCCCACAAGGACTGGAGAGGTGGTAGAACCGCT      600
WashU_Sbay_Contig534.6     GCCACTCAAAAGACTGTCGATGGTCCATCCCACAAGGACTGGAGAGGTGGTAGAACCGCT      552
6257_YJR009C               GCCACTCAAAAGACTGTCGATGGTCCATCCCACAAGGACTGGAGAGGTGGTAGAACCGCT      277
                           ***** *********** ** ***************************************

SGD_Scer_TDH2/YJR009C      TCCGGTAACATCATCCCATCCTCTACCGGTGCTGCTAAGGCTGTCGGTAAGGTCTTGCCA      660
WashU_Sbay_Contig534.6     TCCGGTAACATCATCCCATCCTCCACCGGTGCCGCCAAGGCTGTCGGTAAGGTCTTGCCA      612
6257_YJR009C               TCCGGTAACATCATCCCATCCTCCACCGGTGCCGCCAAGGCTGTCGGTAAGGTCTTGCCT      337
                           *********************** ******** ** ***********************:

SGD_Scer_TDH2/YJR009C      GAATTGCAAGGTAAGTTGACCGGTATGGCTTTCAGAGTCCCAACCGTCGATGTTTCCGTT      720
WashU_Sbay_Contig534.6     GAATTGCAAGGTAAGTTGACCGGTATGGCTTTCAGAGTCCCAACCGTCGATGTCTCCGTT      672
6257_YJR009C               GAATTACAAGGTAAGTTGACCGGTATGGCTTTCAGAGTCCCAACCGTCGATGTCTCCGTT      397
                           *****.*********************************************** ******

SGD_Scer_TDH2/YJR009C      GTTGACTTGACTGTCAAGTTGAACAAGGAAACCACCTACGATGAAATCAAGAAGGTTGTC      780
WashU_Sbay_Contig534.6     GTTGACTTGACTGTTAAGTTGAACAAGGAAACCACCTACGATGAAATCAAGAAGGTTGTC      732
6257_YJR009C               GTTGACTTGACTGTCAAGTTGAACAAGGAAACCACCTACGATGAAATCAAGAAGGTTGTC      457
                           ************** *********************************************

SGD_Scer_TDH2/YJR009C      AAGGCTGCCGCTGAAGGTAAGTTGAAGGGTGTCTTGGGTTACACTGAAGACGCTGTTGTC      840
WashU_Sbay_Contig534.6     AAGGCTGCCGCTGAAGGTAAGTTAAAGGGTGTTTTGGGTTACACTGAAGACGCTGTTGTC      792
6257_YJR009C               AAGGCTGCCGCTGAAGGTAAGTTGAAGGGTGTCTTGGGTTACACTGAAGACGCTGTTGTC      517
                           ***********************.******** ***************************
```

```
SGD_Scer_TDH2/YJR009C       TCCTCTGACTTCTTGGGTGACTCTAACTCTTCCATCTTCGATGCTGCCGCTGGTATCCAA     900
WashU_Sbay_Contig534.6      TCCTCTGACTTCTTGGGTGACGCTAACTCTTCCATCTTCGATGCTTCCGCTGGTATCCAA     852
6257_YJR009C                TCCTCTGACTTCTTGGGTGACTCTAACTCTTCCATCTTCGATGCTGCCGCTGGTATCCAA     577
                            ********************* *********************** **************

SGD_Scer_TDH2/YJR009C       TTGTCTCCAAAGTTCGTCAAGTTGGTTTCCTGGTACGACAACGAATACGGTTACTCTACC     960
WashU_Sbay_Contig534.6      TTGTCTCCAAAGTTCGTCAAGTTGGTCTCCTGGTACGATAACGAATACGGTTACTCTACC     912
6257_YJR009C                TTGTCTCCAAAGTTCGTCAAGTTGGTTTCCTGGTACGACAACGAATACGGTTACTCTACC     637
                            ************************** *********** *********************

SGD_Scer_TDH2/YJR009C       AGAGTTGTCGACTTGGTTGAACACGTTGCCAAGGCTTAA---------------------     999
WashU_Sbay_Contig534.6      AGAGTTGTCGACTTGGTTGAACACGTTGCCAAGGCTTAA---------------------     951
6257_YJR009C                AGAGTTGTCGACTTGGTTGAACACGTTGCCAAGGCTTAAATTTAACTCCTTAAGTACTT     697
```

PRI2/YKL045w, *Sc> Se* at 877-888bp

```
SGD_Scer_PRI2/YKL045W       CAGTTTATCTCAAATGAAGAAAAGGCCGAATTATCTCATCAGTTGTATCAAACAGTTTCT     600
FM1318/1-1587               CAGTTTATTTCCAACGAAGAAAAGGCGGAATTGTCACACCAGTTGTACCAAACCGTCTCA     600
6257_YKL045W                -------------ATGAAGAAAAGGCGGAATTATCTCATCAGTTGTATccnncagtTTCC     47
                                         * *********** *****.**:** ********          **

SGD_Scer_PRI2/YKL045W       GCGTCTCTACAGTTCCAATTGAATTTAAACGAGGAACATCAAAGAAAACAGTATTTCCAA     660
FM1318/1-1587               GCTTCTTTACAGTTCCAATTGAATCTTACTGAAGAACACCAAAGAAGGCAGTACTTCCAA     660
6257_YKL045W                GCGTCTCTACAGTTCCAATTGAATTTAAACGAGGAACATCAAAGAAAACAGTATTTTCAA     107
                            ** *** ***************** *:*. **.***** *******..***** ** ***

SGD_Scer_PRI2/YKL045W       CAGGAAAAATTCATAAAATTACCTTTCGAAAATGTGATAGAACTGGTAGGAAACCGTTTA     720
FM1318/1-1587               CAAGAGAAATTCATAAAACTACCGTTCGAAAACGTGATAGAGCTAGTGGGTAACCGCCTA     720
6257_YKL045W                CAGGAAAAATTCATAAAATTACCTTTCGAAAATGTGATAGAACTGGTAGGAAACCGTTTA     167
                            **.**.************ **** ******** ********.**.**.**:*****  **

SGD_Scer_PRI2/YKL045W       GTGTTTTTGAAGGACGGGTACGCATATTTACCACAATTCCAGCAATTGAATTTACTTTCT     780
FM1318/1-1587               GTGTTTTTGAAAAACGGCTATGCTTATTTGCCGCAGTTCCAACAGCTGAATTTGCTATCC     780
6257_YKL045W                GTGTTTTTGAAGGACGGGTACGCATATTTACCACAATTCCAGCAATTGAATTTACTTTCT     227
                            ***********..**** ** **:*****.**.**.*****.**. *******.**:**

SGD_Scer_PRI2/YKL045W       AATGAGTTTGCTAGCAAATTGAACCAGGAGTTAATAAAAACGTACCAGTACTTACCAAGA     840
FM1318/1-1587               AATGAGTTTGCTAGTAAATTGAACGAGGAGCTATTGAAGACCTACCAGCATCTTCCAAGA     840
6257_YKL045W                AATGAGTTTGCTAGCAAATTAAACCAGGaGTTAATAAAAACGTACCAGTACTTACCAAGA     287
                            ************** *****.*** *** * **:*.**.** ****** *  *:******

SGD_Scer_PRI2/YKL045W       CTGAATGAGGATGACCGGTTGTTACCAATTCTAAATCATCTTTCGTCGGGGTACACTATC     900
FM1318/1-1587               CTGAACGAAGATGACAGGTTGCTGCCAATTTTAAACCATCTTTCGTCAGGGTACACAATT     900
6257_YKL045W                CTGAATGAGGATGACCGGTTGTTACCAATTCTGAATCATCTTTCGTCAGGATACACAATT     347
                            ***** **.******.***** *.****** *.** ***********.**.*****:**

SGD_Scer_PRI2/YKL045W       GCGGATTTCAACCAGCAAAAGGCAAACCAATTCAGTGAGAACGTAGATGATGAGATTAAT     960
FM1318/1-1587               GCAGACTTTAACCAGCAAAAGGCAAACCAATTCGGTGAAAATGTAGACGATGAAATAAAT     960
6257_YKL045W                GCAGACTTTAACCAGCAAAAGGCAAACCAATTCGGTGAAAATGTAGACGATGAAATAAAT     407
                            **.** ** ************************.****.** ***** *****.**:***

SGD_Scer_PRI2/YKL045W       GCGCAAAGTGTCTGGTCTGAAGAGATTAGCTCAAACTATCCGCTATGTATCAAAAACCTG     1020
FM1318/1-1587               GCACAAAGTGTGTGGTCCGAGGAGATCAGCTCGAACTATCCATTGAGTATCAAAAACTTA     1020
6257_YKL045W                GCACAAAGTGTGTGGTCCGAGgAgATCAGCTCGAATTATCCATTGAGTATCAAAAACTTA     467
                            **.******** ***** **. * ** *****.** *****. *.:*********** *.

SGD_Scer_PRI2/YKL045W       ATGGAGGGTCTTAAAAAGAACCATCATTTGAGGTATTATGGGAGACAACAACTGAGTCTG     1080
FM1318/1-1587               ATGGAGGGTTTGAAGAAAAACCATCATTTGAGATATTACGGTAGGCAACAACTAAGCCTG     1080
6257_YKL045W                ATGGAGGGTTTGAAGAAAAACCATCATTTGAGATATTACGGTAGGCAACAACTAAGCCTG     527
                            ********* * **.**.**************.***** ** **.********.** ***
```

```
SGD_Scer_PRI2/YKL045W      TTTTTGAAAGGTATCGGCCTGAGCGCTGATGAAGCGTTAAAATTTTGGTCTGAGGCATTT      1140
FM1318/1-1587              TTTCTAAAGGGAATTGGGTTGAGCGCCGACGAAGCTTTGAAATTTTGGTCAGAAGCTTTC      1140
6257_YKL045W               TTTCTAAAGGGAATTGGGTTGAGCGCCGACGAAGCTTTGAAATTTTGGTCAGAAGCTTTC      587
                           *** *.**.**:** **  ******* ** ***** **.***********:**.**:**

SGD_Scer_PRI2/YKL045W      ACAAGAAATGGGAACATGACAATGGAGAAGTTCAATAAAGAATACCGTTACAGCTTCAGG      1200
FM1318/1-1587              ACAAGAAACGGCAACATGACGATGGAAAAGTTCAATAAAGAGTACCGCTACAGTTTTAGA      1200
6257_YKL045W               ACAAGAAACGGCAACATGACGATGGAAAAGTTCAATAAAGAGTACCGCTACAGTTTTAGA      647
                           ******** ** ********.*****.**************.***** ***** ** **.

SGD_Scer_PRI2/YKL045W      CATAATTACGGTCTTGAAGGTAACAGAATCAACTACAAACCATGGGACTGTCACACTATC      1260
FM1318/1-1587              CACAACTACGGCCTCGAAGGTAACAGAATCAACTACAAACCGTGGGACTGTCACACCATT      1260
6257_YKL045W               CACAACTACGGCCTCGAAGGTAACAGAATCAACTACAAACCGTGGGACTGTCACACCATT      707
                           ** ** ***** ** **************************.************** **

SGD_Scer_PRI2/YKL045W      CTTTCCAAGCCCAGACCTGGGCGCGGAGATTATCATGGATGCCCTTTCCGTGATTGGAGT      1320
FM1318/1-1587              CTATCTAAGCCCAGACCCGGCCGCGGCGATTATCACGGATGCCCATTCCGTGACTGGAGC      1320
6257_YKL045W               CTATCTAAGCCCAGACCCGGCCGCGGCGATTATCACGGATGCcCATTCCGTGACTGGAGC      767
                           **:** *********** ** *****.******** ****** *:******** *****

SGD_Scer_PRI2/YKL045W      CACGAGAGACTATCTGCAGAATTGCGCTCTATGAAGTTGACCCAAGCGCAGATCATCAGT      1380
FM1318/1-1587              CACGACAGACTATCTGCAGAACTGCGTTCCATGAAACTCACCCAAGCACAAATCATAAGT      1380
6257_YKL045W               CACGACAGACTATCTGCAGAACTGCGTTCCATGAAACTCACcCAAGCACAAATCATAAGT      827
                           ***** *************** **** ** *****. * ** *****.**.*****.***

SGD_Scer_PRI2/YKL045W      GTTCTGGATTCCTGCCAGAAAGGTGAATACACAATTGCTTGCACTAAAGTGTTTGAAATG      1440
FM1318/1-1587              GTCCTAGATTCGTGCCAAAAAGGCGAGTACACAATCGCTTGCACCAAAGTATTCGAAATG      1440
6257_YKL045W               GTCCTagaTTCGTGCCAAAnagGCGagTACACAATCGCTTGCACCAAAGTATTCGAAATA      887
                           ** **   *** *****.*   * *  ******** ******** *****.** *****.

SGD_Scer_PRI2/YKL045W      ACACACAACTCTGCATCAGCGGATTTGGAAATTGGCGAGCAAACGCATATCGCGCATCCT      1500
FM1318/1-1587              ACACACAATTCCGCCTCAGCAGACTTGGAGATCGGCGAACAAACTCATATCGCACATCCT      1500
6257_YKL045W               ACACACAATTCCgCCTCAGCAgaCTTGGanaTCGGCGAACAAACTCATATCGcaCATCCT      947
                           ******** **  *.*****.   ****   * *****.***** *******  ******

SGD_Scer_PRI2/YKL045W      AACCTATACTTCGAAAGATCAAGGCAACTGCAAAAGAAACAGCAGAAGCTGGAAAAGGAA      1560
FM1318/1-1587              AACCTCTATTTTGAAAGGTCAAGGCAACTGCAAAAGAAACAACAGAAACCGGAAAAGGAA      1560
6257_YKL045W               AaccTctaTTTTGAAAGGTCaAGGCAACTGCAAAagaAACAACaGAAACCGgaAAAGGAA      1007
                           *    *    ** *****.** *************    ****.* ***.* *  *******

SGD_Scer_PRI2/YKL045W      AAACTATTCAATAATGGTAATCATTAG  1587
FM1318/1-1587              AAACTTTCCAATAGTGCCAATAACCAG  1587
6257_YKL045W               AAACTTTCCAA----------------  1018
                           *****:* ***
```

ALD2/YMR170c, *Se> Sc* at 84-90bp*

```
SGD_Scer_ALD2/YMR170C      ------------------------------------------------------------      0
MIT_Sbay_c933_18910        ------------------------------------------------------------      0
6257_YMR170C               TTTGGAAAATAGCttatcttnttcATCTATCATCTATCATTTTTTtctCGCTTTTGTTtC      60

SGD_Scer_ALD2/YMR170C      ------------------------------------------------------------      0
MIT_Sbay_c933_18910        ------------------------------------------------------------      0
6257_YMR170C               GAGTGACCCGACCCTTAtctAATTTGCCGCCAGAGGCAAGCAGATCGCTATCGCCGTGCT      120

SGD_Scer_ALD2/YMR170C      ------------------------------------------------------------      0
MIT_Sbay_c933_18910        ------------------------------------------------------------      0
6257_YMR170C               TTGCGtctTACTtCTCCACGCCCTTTGAAGCACGGCGGCCTTTAGATACTAATAATATAC      180

SGD_Scer_ALD2/YMR170C      ------------------------------------------------------------      0
MIT_Sbay_c933_18910        ------------------------------------------------------------      0
6257_YMR170C               AATCTAAAACCCTnTACTATACACCCCTCtCCAATGTGCTATCTCCAAGTCGTTTAGGGG      240
```

```
SGD_Scer_ALD2/YMR170C      ------------------------------------------------------------      0
MIT_Sbay_c933_18910        ------------------------------------------------------------      0
6257_YMR170C               TTGGGGACGTGATTATGCTTAATGATCAATGCTCACGTAATTGAAGCACAGTCAATAGGA      300


SGD_Scer_ALD2/YMR170C      ------------------------------------------------------------      0
MIT_Sbay_c933_18910        ------------------------------------------------------------      0
6257_YMR170C               CTTATATAAAAGCGCGAGGCCAGTGAAAATAGTTTCAAATTACTCTCCTTTCTTTCGCCT      360


SGD_Scer_ALD2/YMR170C      ----------------------------------------ATGCCTACCTTGTATACTGA      20
MIT_Sbay_c933_18910        ----------------------------------------ATGCCTAATTTATATACAGA      20
6257_YMR170C               CACTTTTATCAACAAAATCCACAAACAAAACTAAAGCGCCATGCCTGATTTATACACAGA      420
                                                                   ******.. **.** **:**

SGD_Scer_ALD2/YMR170C      TATCGAAATCCCACAATTGAAAATCTCTTTAAAGCAACCGCTAGGGTTGTTTATCAACAA      80
MIT_Sbay_c933_18910        CCTCGAAATCCCACAACTGAAAATTTCTGTTAGACAACCACTTGGGTTGTTCATCAACAA      80
6257_YMR170C               CCTCAAAATCCCACAATTGAAAATCTCTGTTAAACAACCACTTGGGTTGTTCATCAACAA      480
                            .**.*********** ******* *** *:*..*****.**:******** ********

SGD_Scer_ALD2/YMR170C      TGAGTTTTGTCCATCATCAGATGGAAAGACCATCGAAACTGTGAACCCAGCTACTGGCGA      140
MIT_Sbay_c933_18910        CGAATTTTGCCCTTCATCAGACGGCAAAACTATCGAAACTGTAAACCCAAGTACCGGTGA      140
6257_YMR170C               CGAATTTTGTCCATCATCAGATGGAAAAACCATCGAAACTGTGAACCCAGCTACTGGCGA      540
                            **.***** **:******** **.**.** ***********.******. *** ** **

SGD_Scer_ALD2/YMR170C      ACCGATAACATCCTTCCAAGCAGCTAACGAAAAGGATGTAGACAAAGCTGTGAAAGCTGC      200
MIT_Sbay_c933_18910        GGCTATAACCTCTTTCCAAGCCGCTAGCGAAAAGGATGTTGATAAGGCGGTCAAAGCAGC      200
6257_YMR170C               ACCGATAACATCCTTCCAAGCAGCTAACGAAAAGGATGTAGACAAAGCTGTGAAAGCTGC      600
                           . * *****.** ********.****.************:** **.** ** *****:**

SGD_Scer_ALD2/YMR170C      CAGGGCTGCTTTTGATAACGTTTGGTCGAAGACATCTTCTGAGCAACGTGGTATTTATCT      260
MIT_Sbay_c933_18910        CAGAGATGCTTTTGAGAATGTCTGGTCGAAGACATCTGCTGAGCAACGTGGCATATATCT      260
6257_YMR170C               CAGGGCTGCTTTTGATAACGTTTGGTCGAAGACATctTCTGAGcaaCGTGGTATTTATCT      660
                           ***.*.********* ** ** *************   *****   ***** **:*****

SGD_Scer_ALD2/YMR170C      TTCAAACTTATTAAAACTTATTGAGGAGGAGCAAGACACACTTGCCGCATTAGAGACTTT      320
MIT_Sbay_c933_18910        CTCAAACCTACTGAAACTCATCGAAGAAGAACAGGAAACGCTAGCCGCCCTGGAGACTTT      320
6257_YMR170C               TTCAAACTTATTAAAACTTATTGAGGAGGAGcAaGACACACTTGCCGCGTTAGAGACTTT      720
                            ****** ** *.***** ** **.**.**. * **.**.**:*****  *.********

SGD_Scer_ALD2/YMR170C      AGACGCTGGAAAGCCTTACCATTCAAATGCCAAAGGTGATTTGGCACAAATTTTACAGCT      380
MIT_Sbay_c933_18910        AGACGCTGGTAAACCTTTCCATTCTAATGCTATGGGAGATTTAGCTCAAATCATGCAACT      380
6257_YMR170C               AGACGCTGGAAAGCCTTATCATTCAAATGCCAAAGGTGATTTGGCACAAATTTTacAGCT      780
                           *********:**.****: *****:***** *:.**:*****.**:***** :*  *.**

SGD_Scer_ALD2/YMR170C      TACCAGATATTTTGCTGGGTCCGCTGATAAGTTTGACAAAGGTGCAACCATACCATTGAC      440
MIT_Sbay_c933_18910        TACAAGATATTTTGCCGGATCTGCCGATAAGTATAACAAGGGTGATACTATTCCATTATC      440
6257_YMR170C               TACCAGATATTTTGCTGGGTCCGCTGATaAGTTTGAcaaGGGTGCAACCATACCATTGAC      840
                           ***.*********** **.** ** *** ***:*.*   .****.:** **:*****.:*

SGD_Scer_ALD2/YMR170C      TTTTAACAAGTTTGCATATACTCTAAAAGTTCCTTTTGGCGTTGTTGCTCAAATCGTTCC      500
MIT_Sbay_c933_18910        TTCTGAAAAGTTTGCGTACACTTTGAAGGTTCCATTTGGTGTTGTTGCGCAAATCATTCC      500
6257_YMR170C               TTTTAaCaaGTT------------------------------------------------      852
                           ** *. .  ***
```

YMR196w, *Se> Sc* at 2790-2811bp

```
SGD_Scer_YMR196W      GACTTCAAAGTAGAGTGTCCCGTAGGTTCAGGAGATTATTTGAATCTTGCTGAAGTTGCC   2610
FM1318/1-3297         GATTTTAAGGTCGAGTGTCCGGTAGGTTCGGGTGATTATTTAAATCTAGCAGAAGTTGCC   2640
6257_YMR196W          ---------------------------------------------------------TTG   3


SGD_Scer_YMR196W      GAAGAACTTGGATATCGTATGATTCACTTATTTGTTCCAGACGAAAACGGGGAGCGCGCC   2670
FM1318/1-3297         GAAGAACTTGGGTATCGTATGATTCACTTGTTTGTACCAGATGAAAATGGCGAGCGTGCC   2700
6257_YMR196W          CCGANACTTGGGTATCGTATGATTCACTTGTTTGTACCAGATGAAAATGNNNAGCGTGCC   63
                       ... ******.*****************.*****:***** ***** *   **** ***
```

```
SGD_Scer_YMR196W       ATTCATTATGGTGATCACTCTAAGTTTCTGTCTTCTGATCCATATTTCAGGGATTATGTG  2730
FM1318/1-3297          GTTCATTATGGTGATCACTCTAAATTCTTATCCACTGATCCATATTTCAAGGATTATGTA  2760
6257_YMR196W           GTTCATTATGGTGATCACTCTAAATTCTTATCCACTGATCCATATTTCAAGGATTATGTA  123
                       .**********************.**  *.** :***************.*********.

SGD_Scer_YMR196W       CCATTTTTTGAATACTTTGACGGTGATACTGGAAGAGGGCTTGGCGCTTCACACCAATGT  2790
FM1318/1-3297          CCATTCTTTGAATACTTCGATGGTGACACAGGAAGAGGCCTTGGTGCTTCTCACCAGTGT  2820
6257_YMR196W           CCATTCTTTGAATACTTCGATGGTGACTCAGGAAGAGGGCTTGGTGCTTCACACCAATGT  183
                       ***** *********** ** ***** :*:******** ***** *****:*****.***

SGD_Scer_YMR196W       GGTTGGACTGCTCTTGTGGCCAAATGGATAAGTGATGTAGGTATATCCTGTGTAAGACTA  2850
FM1318/1-3297          GGTTGGACTGCTCTTGTGGCTAAATGGATCAGTGATGTAGGAATATCCTGTGTGAGGTTG  2880
6257_YMR196W           GGTTGGACTGCTCTTGTGGCCAAATGGATAAGTGATGTAGGTATATCCTGTGTAAGACTA  243
                       ******************** ********.***********:***********.**. *.

SGD_Scer_YMR196W       CCTCGTACGCCAAGATCATCTGTGGCAACGACCGCTTCAACAGAGAGCTCTGAGCAAGGT  2910
FM1318/1-3297          CCTCGTACTCCAAGATCATCTGTAGCCACCACTGCGTCCACGGAAAGCTCTGAACAAGAA  2940
6257_YMR196W           CCTCGTACGCCAAGATCATCTGTGGCAACGACCGCTTCAACAGAGAGCTCTGAGCAAGGT  303
                       ******** **************.**.** ** ** **.**.**.********.****.:

SGD_Scer_YMR196W       CCCAAAATGAAGAGAATGGCAAGACGTAAGAGTGCAAAGTCTTTGGTAAACTACACTGCC  2970
FM1318/1-3297          CATAAAATGAAAAGAATGGCAAGACGCAAGAGTGCAAAATCCTTAGTGAACTACACTGCC  3000
6257_YMR196W           CCCAAAATGAAGAGAATGGCAAGACGTAAGAGTGCAAAGTCTTTGGTAAACTACACTGCC  363
                       *. ********.************** ***********.** **.**.************

SGD_Scer_YMR196W       ACCATTTTGGACTTAACCGAAGAAGAAAAGCGCCATCATAGGATAGGGGGCACCCATTCT  3030
FM1318/1-3297          ACTATTTTGGACTTAACTGAAGAAGAAAAGCGCCATCACAGAATAGGAGGAACCCATTCT  3060
6257_YMR196W           ACCATTTTGGACTTAACCGAAGAAGAAAAGCGCCATCATAGGATAGGGGGCACCCATTCT  423
                       ** ************** ******************** **.*****.**.*********

SGD_Scer_YMR196W       GGGTTGACACCACAAAGCAGCATTTCAAGTGACAAGGCTAGACATTTGATGGAGGAAATG  3090
FM1318/1-3297          GGATTGACGCCACAAAGTAGTATTTCAAGTGATAAGGCAAAAAAACTCATGGAGGAAATG  3120
6257_YMR196W           GGGTTGACACCACAAAGCAGCAATTCAAGTGACAAGGCTAGACATTTGATGGAGGAAATG  483
                       **.*****.******** ** *:********* *****:*.*.*: * ************

SGD_Scer_YMR196W       AATGAAGAGGAAGGTATTCACGAAACTGTGGTACCTGAAGATCGTCACAACTTTGAAACC  3150
FM1318/1-3297          AATGATGAGGAAGGTATTCATGAAGTTGTGGTGCGAGAAGATCGTCATGAATTCGAAACA  3180
6257_YMR196W           AATGAAGAGGAAGGTATTCACGAAACTGTGGTACCTGAAGATCGTCACAACTTTGAAACC  543
                       *****:************** ***. ******.* :*********** .*.** *****.

SGD_Scer_YMR196W       AAGCTTATAGGCAAGCTAAAAGATAAGGTGAAAAATATGAAAGTAACTGACAAGGCTAAA  3210
FM1318/1-3297          AAATTGATAAGCAAGCTAAAGGATAAAGTGAAAAATATGAAGGTAAGCGATAAGACTAAA  3240
6257_YMR196W           AAGCTTATAGGCAAGCTAAAAGATAAGGTGAAAAATATGAAAGTAACTGACAAGGCTAAA  603
                       **. * ***.**********.*****.**************.****  ** ***.*****

SGD_Scer_YMR196W       GATGAGGATATAGACCCAATGGACCCAATGAGTCCGTTGAATAAG-GATGTGTCTTGA--  3267
FM1318/1-3297          GACGAAGATATTGATCCAATGGATCCAATGTGTCCGTTGAATAAAGATTTATCTTGA---  3297
6257_YMR196W           GATGAGGCTATAGACCCAATGGACCCGATGAGTCCGTTTGAATAANGATGTGTCTTGATT  663
                       ** **.*.***:** ******** **.***:******* .*::*. .:* :   * .

SGD_Scer_YMR196W       ------------------------          3267
FM1318/1-3297          ------------------------          3297
6257_YMR196W           AACTGCGGTGAACCTTTCAATATC          687
```

HSP82/YPL240c, Se>*Sc* at 1578-1608bp*

```
SGD_Scer_HSP82/YPL240C     AAGTTGTTACGTTACAACTCTACCAAGTCCGTAGATGAGTTGACTTCCTTAACTGATTAC     1380
MIT_Sbay_c60_24336         AAATTGCTACGTTACAACTCCACCAAGTCCGTTGACGAATTGACTTCTTTGACTGATTAT     297
6257_YPL240C               -----------------------------------------------------------C     1


SGD_Scer_HSP82/YPL240C     GTTACCAGAATGCCAGAACACCAAAAGAACATCTACTACATCACTGGTGAATCTCTAAAG     1440
MIT_Sbay_c60_24336         ATTACCAGAATGCCAGAACACCAAAAGAACATCTACTATATCACAGGTGAGTCTTTGAAG     357
6257_YPL240C               ATCACCAGAATGCCAGAACAcCAAAAGAACATTTACTACATCACAGGTGAATCTCTAAAG     61
                           .* **************** *********** ***** *****:*****.*** *.***

SGD_Scer_HSP82/YPL240C     GCTGTCGAAAAGTCTCCATTTTTGGATGCCTTGAAGGCTAAAAACTTCGAGGTTTTGTTC     1500
MIT_Sbay_c60_24336         GCTGTTGAAAAATCCCCATTCTTAGACGCTTTGAAAGCTAAGAACTTTGAAGTTTTGTTC     417
6257_YPL240C               GCCGTTGAAAAATCCCCATTCTTGGACGCTTTGAAGGCTAAGAACTTTGAAGTTTTGTTC     121
                           ** ** *****.** ***** **.** ** *****.*****.***** **.*********
```

```
SGD_Scer_HSP82/YPL240C      TTGACCGACCCAATTGATGAATACGCCTTCACTCAATTGAAGGAATTCGAAGGTAAAACT     1560
MIT_Sbay_c60_24336          TTGACTGATCCAATCGATGAATACGCCTTCACTCAATTAAAGGAATTCGAAGGTAAGACT     477
6257_YPL240C                TTGACTGATCCAATCGATGAATACGCTTTCACCCAATTGAAGGAATTCGAAGGTAAGACT     181
                            ***** ** ***** *********** ***** *****.*****************.***

SGD_Scer_HSP82/YPL240C      TTGGTTGACATTACTAAAGATTTCGAATTGGAAGAAACTGACGAAGAAAAAGCTGAAAGA     1620
MIT_Sbay_c60_24336          TTAGTCGATATCACCAAGGATTTCGAGCTGGAAGAAACTGACGAAGAGAAAGCTGAAAGA     537
6257_YPL240C                TTGGTCGATATCACCAAGGATTTCGAACTGGAAGAAACTGACGAAGAAAAAGCTGAAAGA     241
                            **.** ** ** ** **.********. *******************.************

SGD_Scer_HSP82/YPL240C      GAGAAGGAGATCAAAGAATATGAACCATTGACCAAGGCCTTGAAAGAAATTTTGGGTGAC     1680
MIT_Sbay_c60_24336          GAAAAGGAAGTTAAAGAATTCGAACCATTGACCAAGGCCTTGAAAGACATCTTGGGTGAA     597
6257_YPL240C                GAGAAGGAGATCAAAGAATATGAACCATTGACCAAGGCCTTGAAAGAAATTTTGGGTGAC     301
                            **.*****..* *******: **************************.** ********.

SGD_Scer_HSP82/YPL240C      CAAGTGGAGAAAGTTGTTGTTTCTTACAAATTGTTGGATGCCCCAGCTGCTATCAGAACT     1740
MIT_Sbay_c60_24336          CAAGTTGAAAAGGTTGTTGTCTCTTACAAACTAGTGGATGCCCCAGCTGCCATTAGAACT     657
6257_YPL240C                CAAGTGGAGAAAGTTGTTGTTTCTTACAAATTGTTGGATGCCCCAGCTGCTATCAGAACT     361
                            ***** **.**.******** ********* *. **************** ** ******

SGD_Scer_HSP82/YPL240C      GGTCAATTTGGTTGGTCTGCTAACATGGAAAGAATCATGAAGGCTCAAGCCTTGAGAGAC     1800
MIT_Sbay_c60_24336          GGCCAATTCGGTTGGTCCGCTAACATGGAAAGAATCATGAAGGCTCAAGCTTTGAGAGAC     717
6257_YPL240C                GGTCAATTTGGTTGGTCTGCTAAcaTGGAAAGAATCATGAAGGCTCAAGCCTTGAGAGAC     421
                            ** ***** ******** *****  ************************* *********

SGD_Scer_HSP82/YPL240C      TCTTCCATGTCCTCCTACATGTCTTCCAAGAAGACTTTCGAAATTTCTCCAAAATCTCCA     1860
MIT_Sbay_c60_24336          TCTTCCATGTCCTCTTACATGTCCTCCAAGAAGACTTTCGAAATCTCTCCAAAATCTCCA     777
6257_YPL240C                TCTTCCATGTCCTCCTACATGTCTTCCAAGAAGACTTTCGAAATTTCTCCAAAATCTCCA     481
                            ************** ******** ******************** ***************

SGD_Scer_HSP82/YPL240C      ATTATCAAGGAATTGAAAAAGAGAGTTGACGAAGGTGGTGCTCAAGACAAGACTGTCAAG     1920
MIT_Sbay_c60_24336          ATTATCAAGGAATTGAAAAAGAGAGTTGATGAAGGCGGTGCTCAAGATAAGACTGTCAAG     837
6257_YPL240C                ATTATCAAGGAATtgAAAAAGAGAGTtGaCGAAGGTGGTGCTCAAGACAAGACtGtCAAG     541
                            *************  *********** *  ***** *********** ***** * ****

SGD_Scer_HSP82/YPL240C      GACTTGACTAAGTTATTATATGAAACTGCTTTGTTGACTTCCGGCTTCAGTTTGGACGAA     1980
MIT_Sbay_c60_24336          GATTTGACCAACTTATTATTCGAAACCGCTCTGTTAACTTCTGGTTTCAGTCTGGAAGAA     897
6257_YPL240C                GactgaCtaagTt-----------------------------------------------     554
                            *      .
```

GPH1/YPR160W, *Sc*> *Se* at1449-1464bp

```
SGD_Scer_GPH1/YPR160W      TTGTATCCAAACGATAACTTTGCTCAAGGTAAGGAGTTGAGGTTGAAACAGCAGTACTTC      1071
FM1318/1-2718              CTGTATCCAAACGACAACTTTGCCCAAGGTAAAGAACTGAGATTGAAACAGCAATACTTC      1080
6257_YPR160W               -----------------------------------GTTGAGGTTGAAACAGCAGTACTTC      25
                                                              . ****.***********.******

SGD_Scer_GPH1/YPR160W      TGGTGTGCTGCATCCTTACACGACATCTTAAGAAGATTCAAAAAATCCAAGAGGCCATGG      1131
FM1318/1-2718              TGGTGTGCTGCATCCCTGCACGATATCCTAAGAAGATTCAAAAAATCCAAGAGGTCATGG      1140
6257_YPR160W               TGGTGTGCTGCATcCttACACGACATCTTAAGAAGATTCAAAAAATCCAAGAGGCCATGG      85
                           ************* *  .***** *** ************************** *****

SGD_Scer_GPH1/YPR160W      ACTGAATTTCCTGACCAAGTGGCTATTCAGTTGAATGATACTCATCCAACTTTAGCCATC      1191
FM1318/1-2718              ACCGAGTTCCCTGAACAAGTGGCTATTCAATTGAACGATACTCATCCAACTTTAGCTATC      1200
6257_YPR160W               ACTGAATTTCCTGACCAAGTGGCTATTCAGTTGAATGATACTCATCCAACTTTAGCCATC      145
                           ** **.** *****.**************.***** ******************** ***

SGD_Scer_GPH1/YPR160W      GTTGAATTACAGAGAGTTTTGGTCGATCTAGAAAAACTAGATTGGCACGAGGCTTGGGAC      1251
FM1318/1-2718              CTTGAATTGCAAAGAGTCTTGGTTGATTTGGAAAAATTGGACTGGCACGAAGCTTGGGAC      1260
6257_YPR160W               GTTGAATTACAGAGAGTTTTGGTCGATCTAGAAAAACTAGATTGGCACGAGGCTTGGGAC      205
                            *******.**.***** ***** *** *.****** *.** ********.*********

SGD_Scer_GPH1/YPR160W      ATCGTGACCAAGACTTTTGCTTATACTAACCACACTGTTATGCAAGAGGCCCTGGAAAAA      1311
FM1318/1-2718              ATTGTCACCAAGACCTTTGCTTATACCAACCACACCGTTATGCAGGAAGCCTTGGAAAAA      1320
6257_YPR160W               ATCGTGACCAAGACTTTTGCTTATACTAACCACACTGTTATGCAAGAGGCCCTGGAAAAA      265
                           ** ** ******** *********** ******** ********.**.*** ********
```

```
SGD_Scer_GPH1/YPR160W     TGGCCCGTCGGCCTCTTTGGCCATTTGCTACCCAGACATTTGGAAATTATATATGATATC     1371
FM1318/1-2718             TGGCCCGTTGGCTTGTTCGGCCATTTGTTGCCCAGACATCTGGAAATCATTTACGATATT     1380
6257_YPR160W              TGGCCCGTCGGCCTCTTTGGCCATTTGCTACCCAGACATTTGGAAATTATATATGATATC     325
                          ******** *** * ** ********* *.********* ******* **:** *****

SGD_Scer_GPH1/YPR160W     AACTGGTTCTTCTTGCAAGATGTGGCCAAAAAATTCCCCAAGGATGTTGATCTTTTGTCT     1431
FM1318/1-2718             AATTGGTTCTTCTTGCAAGATGTTGCAAAGAAATTCCCCAAGGATGTTGATCTTTTGTCT     1440
6257_YPR160W              AACTGGTTCTTCTTGCAAGATGTGGCCAAAAAATTCCCCAAGGATGTTGATCTTTTGTCT     385
                          ** ******************** **.**.******************************

SGD_Scer_GPH1/YPR160W     CGTATATCCATCATCGAAGAAAACTCTCCAGAAAGACAGATCAGAATGGCCTTTTTGGCT     1491
FM1318/1-2718             CGTATATCCATCATCGAGGAAAACTCTCCAGAGAGACAGATCAGAATGGCCTTTTTGGCT     1500
6257_YPR160W              CGTATATCCATCATCGAAGAAAACTCTCCAGAGAGACAGATCAGAATGGCCTTTTTGGCT     445
                          *****************.**************.***************************

SGD_Scer_GPH1/YPR160W     ATTGTTGGTTCACACAAGGTTAATGGTGTTGCTGAATTGCACTCTGAATTAATCAAAACG     1551
FM1318/1-2718             ATTGTTGGTTCTCATAAAGTCAACGGTGTTGCGGAATTGCACTCTGAATTAATTAAGACC     1560
6257_YPR160W              ATTGTTGGTTCTCATAAAGTCAACGGTGTTGCGGAATTGCACTCTGAATTAATTAAGACC     505
                          ***********:** **.** ** ******** ******************** **.**

SGD_Scer_GPH1/YPR160W     ACCATATTTAAAGATTTTGTCAAGTTCTATGGTCCATCAAAGTTTGTCAATGTCACTAAC     1611
FM1318/1-2718             ACCATCTTCAAAGATTTCGTCAAATTCTACGGTGCATCAAAGTTTGTCAACGTTACTAAC     1620
6257_YPR160W              ACCATCTTCAAAGATTTCGTCAAATTCTACGGTGCATCAAAGTTTGTCAACGTTACTAAC     565
                          *****.** ******** *****.***** *** **************** ** ******

SGD_Scer_GPH1/YPR160W     GGTATCACACCAAGGAGATGGTTGAAGCAAGCTAACCCTTCATTGGCTAAACTGATCAGT     1671
FM1318/1-2718             GGTATCACACCAAGAAGATGGTTGAAGCAAGCTAACCCTAACTTGGCTAGATTGATTAGC     1680
6257_YPR160W              GGTATCACACCAAGAAGATGGTTGAAGCAAGCTAACCCTAACTTGGCTAGATTGATTAGC     625
                          **************.************************:..*******.* **** **

SGD_Scer_GPH1/YPR160W     GAAACCCTTAACGATCCAACAGAGGAGTATTTGTTGGACATGGCCAAACTGACCCAGTTG     1731
FM1318/1-2718             AAAACTCTTAACGATCCTACAGAGGACTATCTACTAGACATGACAAAGTTAACTCAACTG     1740
6257_YPR160W              AAAACTCTTAACGATCCTACAGAGGACTATCTACTAGACATGACAAAGTTAACTCAACTG     685
                          .**** ***********:******** *** *. *.******.*.**. *.** **. **

SGD_Scer_GPH1/YPR160W     GGAAAATATGTTGAAGATAAGGAGTTTTTGAAAAAATGGAACCAAGTCAAGCTTAATAAT     1791
FM1318/1-2718             GCAAAGCACCTTGAGGATAAGAAGTTTTTGAAAGAGTGGAATCAAGTCAAACTCAATAAT     1800
6257_YPR160W              GCAAAGCACCTTGAGGATAAGAAGTTTTTGAAAGAGTGGAATCAAGTCAAACTCAATAAT     745
                          * ***. *  ****.******.***********.*.***** ********.** ******

SGD_Scer_GPH1/YPR160W     AAGATCAGATTAGTAGATTTAATCAAAAAGGAAAATGATGGAGTAGACATCATTAACAGA     1851
FM1318/1-2718             AAGATCAGATTGGTGGACCTAATCAAAAAAGAAAATGGTGGTGAAGACATCATTAACAGA     1860
6257_YPR160W              AAGATCAGATTGGTGGACCTAATCAAAAAaGAAAATGGTGGTGAAGACATCATTAACAGA     805
                          ***********.**.**  ********** *******.***:*:****************

SGD_Scer_GPH1/YPR160W     GAGTATTTGGACGACACCTTGTTTGATATGCAAGTTAAACGTATTCATGAATATAAGCGT     1911
FM1318/1-2718             GAGTATCTAGACGATACTTTGTTTGATATGCAAGTTAAACGTATTCACGAGTATAAACGT     1920
6257_YPR160W              GAGTATCTAGACGATACTtTGTTTGATATGCAAGTTAAACGTATTCACGAGTATAAACGT     865
                          ****** *.***** **  **************************** **.*****.***

SGD_Scer_GPH1/YPR160W     CAACAGCTAAACGTCTTTGGTATTATATACCGTTACCTGGCAATGAAGAATATGCTGAAG     1971
FM1318/1-2718             CAACAACTAAACGTCTTTGGTATTATTTACCGTTACTTAGCAATGAAAAATATGCTAGAG     1980
6257_YPR160W              CAACAACTAAACGTCTTTGGTATTATTTACCGTTACTTAGCAATGAAAAATATGCTAgag     925
                          *****.********************:********* *.********.********.

SGD_Scer_GPH1/YPR160W     AACGGTGCTTCGATCGAAGAAGTTGCCAAGAAATATCCACGCAAGGTTTCAATCTTTGGT     2031
FM1318/1-2718             AACGGTGCTTCCATCGAGGAAGTGGCCAAGAAATATCCACGTAAGGTTTCTATCTTCGGT     2040
6257_YPR160W              aACGGTGCTTCCATCGAGGAAGTGGCCAanaAATATCCACGTAAGGTTTCTATCTTCGgt     985
                           ********** *****.***** ****   ********** ********:***** *

SGD_Scer_GPH1/YPR160W     GGTAAGAGTGCTCCTGGTTACTACATGGCTAAGCTGATCATAAAATTGATCAACTGTGTT     2091
FM1318/1-2718             GGTAAGAGTGCACCCGGTTACTACATGGCTAAGTTGATCATCAAACTGGTCAACTCTGTC     2100
6257_YPR160W              GGTAanantGCaCCCGGTTACTACATGGCTAAgtTGatcATCAAaCTGGTCAACTCTGTc     1045
                          ****     ** ** *****************  **   **.**  **.****** ***

SGD_Scer_GPH1/YPR160W     GCTGACATTGTTAATAACGACGAGTCAATTGAGCATTTGTTGAAGGTTGTCTTTGTTGCT     2151
FM1318/1-2718             GCTGAAATTGTTAACAACGACGAATCAATCGACGACTTATTGAAAGTTGTCTTCATTGCT     2160
6257_YPR160W              ncTgaAATTGTtaaCAACGACGAATCAATcGACGaCTTAT--------------------     1085
                            *  .*****    ********.***** **    **.*
```

QCR2/YPR191w, *Se> Sc* at 574-579bp

```
SGD_Scer_QCR2/YPR191W      TATGCAACCAAGGATGGTGTGGCCCATCTTTTAAACAGATTCAACTTTCAAAACACGAAC      180
FM1318/1-1107              TATGCAACCAAGGACGGTGTAGCCCATCTTTTGAACAGGTTCAACTTCCAGAATACAAAT      180
6257_YPR191W               --------------------AGCCcATCTTTTGAACAGGTTCAACTTCCaGaACACAAAT      40
                                               .*** *******.*****.******** *  .  * **.**

SGD_Scer_QCR2/YPR191W      ACTAGATCAGCTTTGAAATTAGTCAGAGAATCCGAATTATTAGGGGGAACTTTTAAGTCT      240
FM1318/1-1107              GCTAGGTCTGCGTTGAGATTGGTCAGAGAATCCGAATTATTAGGGGGAAATTTTAAGTCC      240
6257_YPR191W               GCTAGGTCTGCGTTGAGATTGGTCAGAGAATCCGAATTATTAGGGGGAAATTTTAAGTCC      100
                           .****.**:** ****.***.***************************.*********

SGD_Scer_QCR2/YPR191W      ACCTTGGATAGGGAATACATCACTTTGAAAGCTACCTTTTTGAAGGACGACCTTCCCTAC      300
FM1318/1-1107              ACTTTGGATAGGGAATACATCACTCTAAAAGCTACATTCTTGAGGGACGACCTTCCTTAC      300
6257_YPR191W               ACTTTGGATAGGGAATACATCACTCTAAAAGCTACATTCTTGAGGGACGACCTTCCTTAC      160
                           ** ********************* *.********.** ****.************ ***

SGD_Scer_QCR2/YPR191W      TACGTCAATGCCCTAGCAGACGTGCTATACAAGACTGCCTTCAAACCTCACGAGCTCACC      360
FM1318/1-1107              TATGTCAATGCCTTGGCAGATGTGTTGTATAAGACTGCCTTCAAACCCCACGAGCTGTCT      360
6257_YPR191W               TACGTCAATGCCTTGGCAGATGTGTTGTATAAGACTGCCTTCAAACCCCACGAGCTGTCT      220
                           ** ********* *.***** *** *.** ***************** ******** :*

SGD_Scer_QCR2/YPR191W      GAATCTGTTTTGCCTGCTGCTAGATACGATTATGCGGTCGCTGAACAATGTCCGGTAAAG      420
FM1318/1-1107              GAATCTGTTTTGCCTGCTGCCAGATACGATTATGCGGTTGCTGAGCAGTGCCCCGTAAAA      420
6257_YPR191W               GAATCTGTTTTGCCTGCTGCCAGATACGATTATGCGGTTGCTGAGCAGTGCCCCGTAAAA      280
                           ******************** ***************** *****.**.** ** *****.

SGD_Scer_QCR2/YPR191W      AGCGCCGAAGACCAATTGTATGCCATTACATTCAGAAAGGGTTTAGGAAACCCATTGTTA      480
FM1318/1-1107              AGCGCAGAAGAACAGTTATTCGCTATTACATTCAGAAAGGGCTTGGGAAACCCATTGTAT      480
6257_YPR191W               AGCGCAGAAGAACAGTTATTCGCTATTACATTTAGAAAGGGCTTGGGAAATCCATTGTAT      340
                           *****.*****.**.**.*: ** ******** ******** **.***** *******::

SGD_Scer_QCR2/YPR191W      TACGATGGTGTGGAAAGAGTCAGTTTGCAAGATATCAAGGACTTTGCGGACAAAGTCTAT      540
FM1318/1-1107              TACGACGGCGTGGAAAAAGTCAGCTTGCAAGATATCAAGGATTACGCTGACAAAGTCTAC      540
6257_YPR191W               TACGACGGTGTGGAAAAAGTCAGCTTGCAAGATATCAAGGATTACGCTGaCAAAGTCTAC      400
                           ***** ** *******.****** **************** *: ** * *********

SGD_Scer_QCR2/YPR191W      ACCAAGGAGAACCTTGAAGTTAGCGGTGAAAATGTTGTTGAGGCCGATTTGAAAAGATTT      600
FM1318/1-1107              ACTAAAGAGAATCTTGAAATTACAGGTGAAAATATTGTCGAGGCCGATTTGAAAAGGTTT      600
6257_YPR191W               ACTAAAGAGAATCTTGAAATTACAGGTGAAAATATTGTTGAGGCCGATTTGAAAAGATTT      460
                           ** **.***** ******.*** .*********.**** *****************.***

SGD_Scer_QCR2/YPR191W      GTTGACGAGTCACTGTTAAGCACTTTGCCTGCAGGTAAGTCATTGGTGAGTAAATCCGAA      660
FM1318/1-1107              GTTGACGATTCCTTGTTGGCCACCTTGCCCACAGGCAAATCGTTGGTAAGCAAATCCGAA      660
6257_YPR191W               GTTGACGAGTCACTGTTAAGCACTTTGCCTGCAGGTAAGTCATTGGTGAGTAAATCCGAA      520
                           ******** **. ****.. *** ***** .**** **.**.*****.** *********

SGD_Scer_QCR2/YPR191W      CCAAAATTCTTTTTGGGTGAAGAAAACAGGGTAAGGTTTATCGGTGACTCCGTTGCCGCC      720
FM1318/1-1107              CCAAAATCTTTCCTAGGTGAAGAAAACAGACTAAGATTCCTCGGTGAGTCCGTTGCTGCC      720
6257_YPR191W               CCAAAATCCTTTTTGGGTGAAGAAAACAGGGTAAGGTTTATCGGTGACTCCGTTGCCGCC      580
                           *******  **  *.**************.  ****.** .******* ******** ***

SGD_Scer_QCR2/YPR191W      ATTGGTATCCCGGTAAACAAAGCCTCCCTAGCTCAATATGAAGTATTGGCCAACTATTTG      780
FM1318/1-1107              ATCGGTGTTCCTGTGAACAAGGCATCTTTAGCCCAATACGAAGTCTTGGCCAGCTATTTG      780
6257_YPR191W               ATTGGTATCCCGGTAAACAAAGCCTCCCTAGCTCAATATGAAGTATTGGCCAACTATTTG      640
                           ** ***.* ** **.*****.**.**  **** ***** *****.*******.*******

SGD_Scer_QCR2/YPR191W      ACCTCTGCCCTATCCGAGCTTTCCGGTTTAATCAGCTCGGCTAAACTTGATAAATTCACT      840
FM1318/1-1107              ACCTCCGCACTTTCCGATGTATCCGGCTTGGTCAACACTGCCAAAGTAGAAAAATTCAGT      840
6257_YPR191W               ACCTCTGCCCTATCCGACCTTTCCGGCTTAGTCAGCTCGGCTAAACTTGATAAATTCACT      700
                           ***** **.**:*****  *:***** **..***.*:* ** *** *:**:******* *

SGD_Scer_QCR2/YPR191W      GACGGCGGCCTATTTACTCTGTTTGTAAGAGACCAGGACAGCGCCGTGGTATCTTCCAAC      900
FM1318/1-1107              GACGGTGGTCTCTTCACTTTGACTGTCAGAAATCAAGACAGCTCTGTGGTGTCTGNAAAC      900
6257_YPR191W               GACGGCGGCCTGTTTACTCTGTTTGTAAGAgACCAAGACAGCGCCGTGGTATCTTCCAAC      760
                           ***** ** ** ** *** **: ***.*** * **.****** * *****.***  .***

SGD_Scer_QCR2/YPR191W      ATCAAGAAAATTGTTGCGGATTTGAAGAAGGGCAAGGACTTATCCCCTGCAATAAATTAC      960
FM1318/1-1107              ATTAAGAAGATTGTTGCAGACTTGAAAAAGGGTAAGGATTTAGCACCTGCTGTAAATTAC      960
6257_YPR191W               ATCAAGAAAATTGTTGCGGATTTGAAgaagGGCAAGGACTTATCCCCTGCaatAATTACA      820
                           ** *****.********.** *****    ** ***** *** *.*****   **:*:..

SGD_Scer_QCR2/YPR191W      ACAAAGTTAAAGAATGCCGTCCAAAATGAATCTGTTTCCAGCCCAATTGAACTAAATTTT      1020
FM1318/1-1107              ACTCAATTGAAGAACGCTGTACAGAATGGNNNNNNNNNNNNNNNNNNNTGTTTTGAAACTC      1020
6257_YPR191W               CAAAgtTAA---------------------------------------------------      829
                           ..:.  *:.
```
