# Supplementary material for: Sequencing and Characterisation of Rearrangements in Three S. pastorianus Strains Reveals the Presence of Chimeric Genes and Gives Evidence of Breakpoint Reuse
Source: PLoS One. 2014 Mar 18;9(3):e92203. doi: 10.1371/journal.pone.0092203 (PMC3958482; doi:10.1371/journal.pone.0092203)

S. cerevisiae subgenome clustering

DBVPG 6033

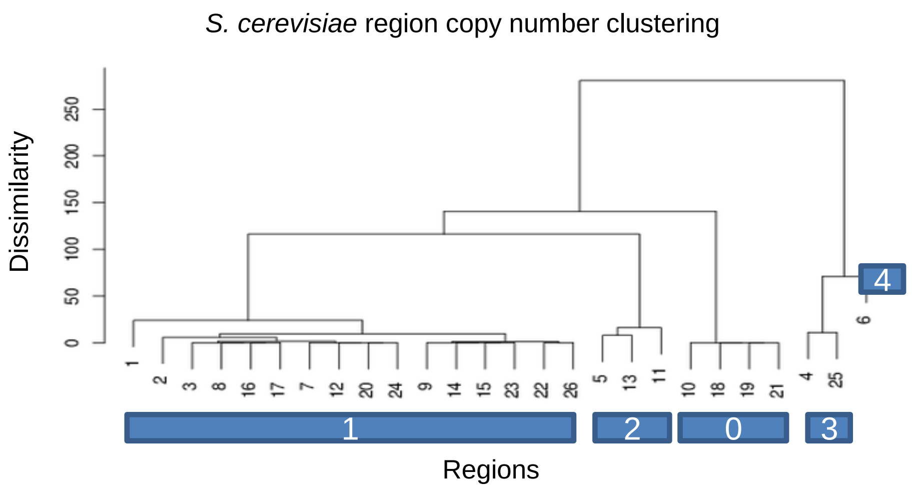

DBVPG 6261

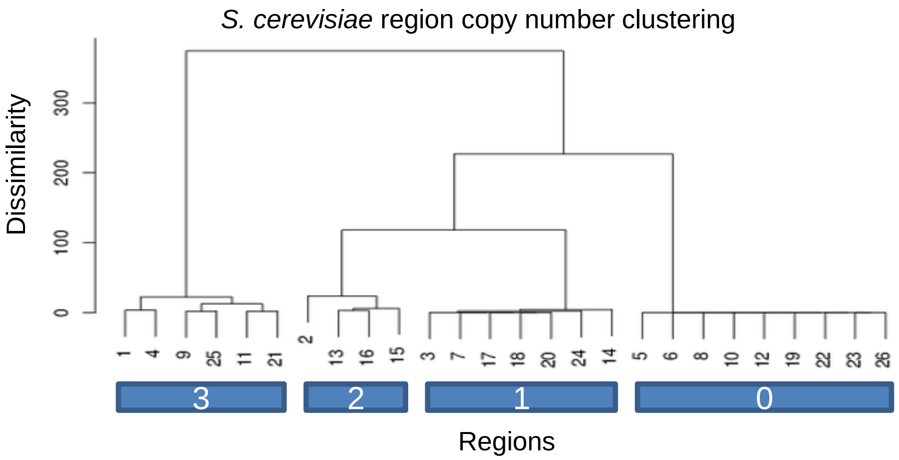

DBVPG 6257

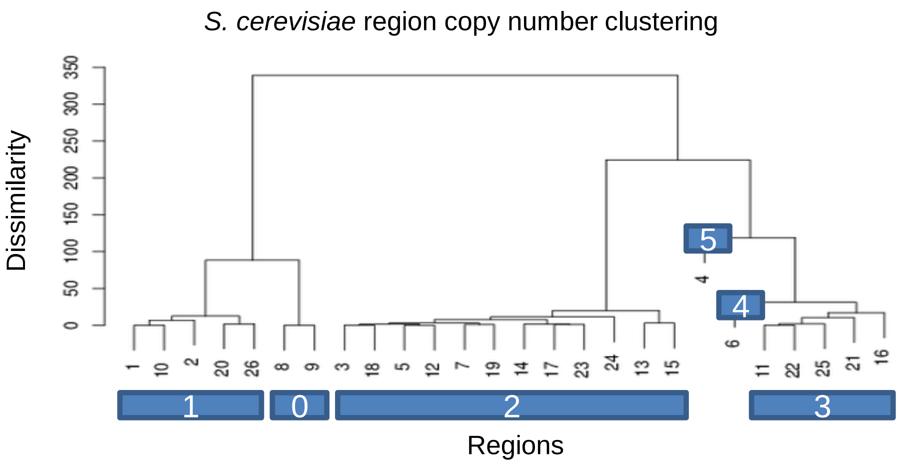

S. eubayanus subgenome clustering

DBVPG 6033

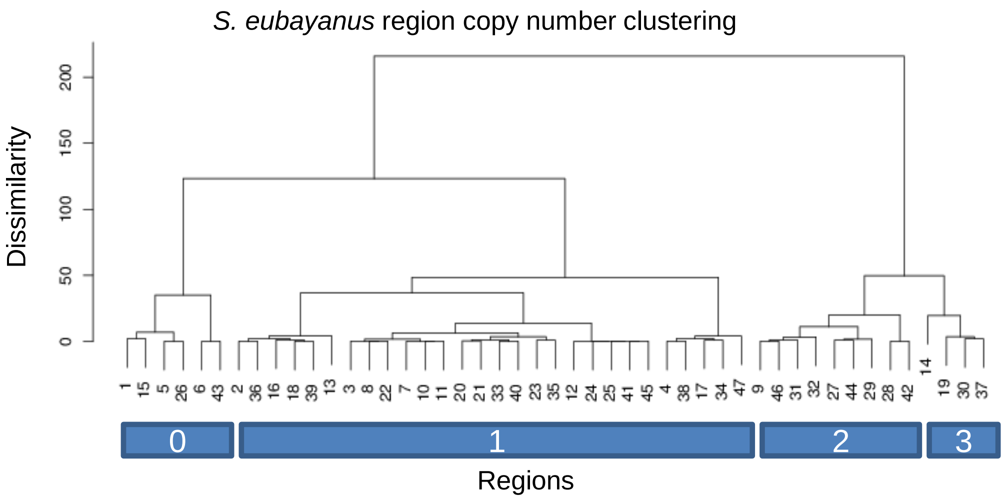

DBVPG 6261

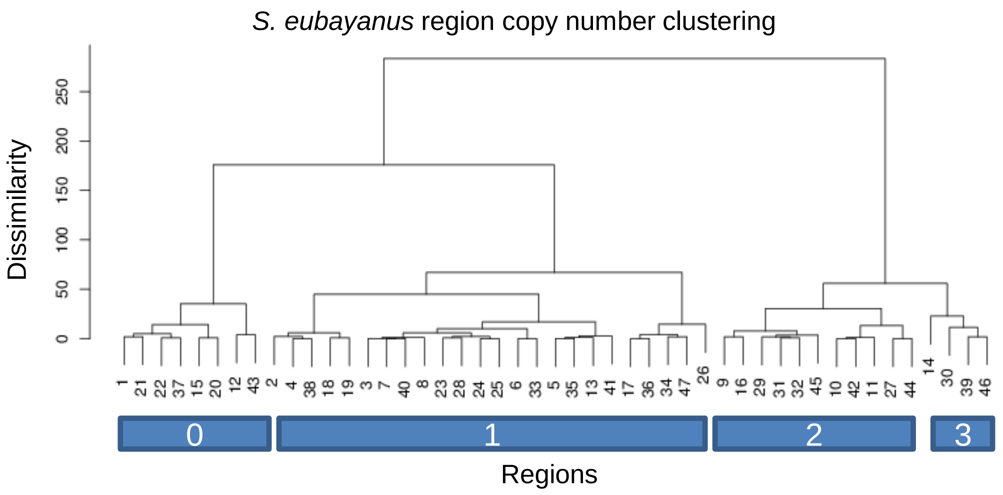

DBVPG 6257

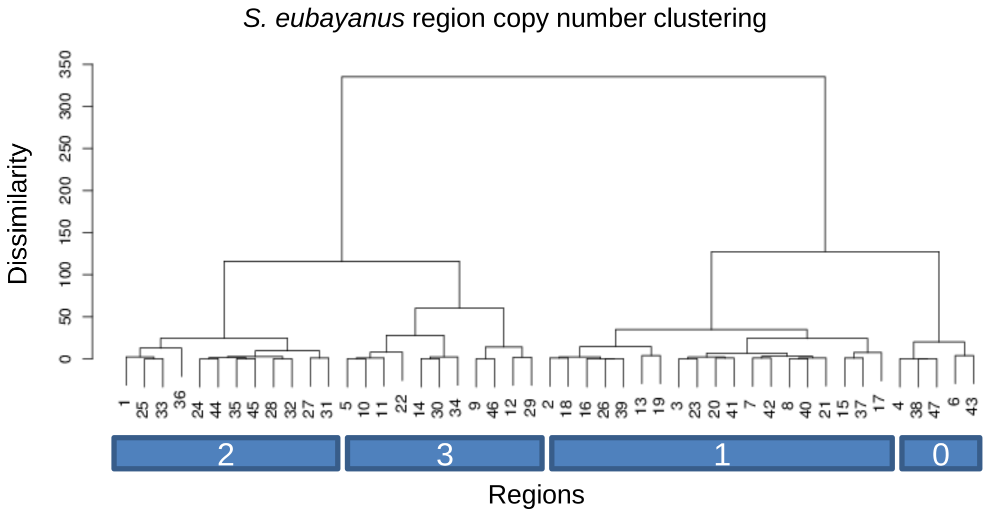

Supplement: Figure S3 — Hierarchical cluster analysis of read depth of S. pastorianus chromosomes. The median read depth for 26 regions covering the S. cerevisiae-like chromosomes (shown in Figure 1) and 47 regions across the S. eubayanus-like chromosomes (sample contigs across each chromosome) were clustered independently and for each strain of S. pastorianus. Results from each hierarchical cluster analysis using Ward’s method are shown as a dendrogram. The blue boxes indicate the copy number assigned to that cluster. (PDF) [file pone.0092203.s003.pdf]
