# Supplementary material for: Sequencing and Characterisation of Rearrangements in Three S. pastorianus Strains Reveals the Presence of Chimeric Genes and Gives Evidence of Breakpoint Reuse
Source: PLoS One. 2014 Mar 18;9(3):e92203. doi: 10.1371/journal.pone.0092203 (PMC3958482; doi:10.1371/journal.pone.0092203)
Supplement: Table S1 — Breakpoints which were not successfully amplified by PCR. (DOC) [file pone.0092203.s004.doc]

**Table S1. Breakpoints which were not successfully amplified by PCR.**

| **DBVPG strain** | **Genome position*** | **Systematic name** | **Standard name** | **Notes** |
| --- | --- | --- | --- | --- |
| 6033 | chrIII:201,200 | Intergenic | Intergenic | Encompasses MATALPHA |
|  | chrX:710,200 | Intergenic | Intergenic | ARS 1022 |
|  | chrXIII:123,200 | Intergenic | Intergenic | *S. uvarum* transposon |
|  | chrXV:575,150 | YOR133w | EFT1 | - |
| 6261 | chrVIII:505,000 | YHR203c | RPS4B | - |
|  | chrX:702,580 | YJR145c | RPS4A | - |
| 6257 | chrIII:201,200 | Intergenic | Intergenic | Encompasses MATALPHA |
|  | chrX:531,500 | Intergenic | Intergenic | LTR |
|  | chrX:543,330 | Intergenic | Intergenic | LTR |
|  | chrX:710,200 | Intergenic | Intergenic | ARS |
|  | chrXII:599,000 | Intergenic | Intergenic | TY1 LTR |
|  | chrXIII:870,900 | YMR302c | PRP12 | - |
|  | chrXIV:82,000 | YNL293c | MSB3 | TY1-LTR |
|  | chrXIV:132,000 | YNL271c | BNI1 | TY1-LTR |

* Approximation based on reads mapped to *S. cerevisiae* sequence UCSC SacCer2 June 2008.
