# Supplementary material for: Sequencing and Characterisation of Rearrangements in Three S. pastorianus Strains Reveals the Presence of Chimeric Genes and Gives Evidence of Breakpoint Reuse
Source: PLoS One. 2014 Mar 18;9(3):e92203. doi: 10.1371/journal.pone.0092203 (PMC3958482; doi:10.1371/journal.pone.0092203)
Supplement: Table S2 — Analysis of the breakpoint region in each strain of S. pastorianus . (DOC) [file pone.0092203.s005.doc]

**Table S2. Analysis of the breakpoint region in each strain of *S. pastorianus*.**

| **DBVPG Strain** | **Genome position^a^** | **Systematic name** | **Standard name** | **Gene sc:su nt identity^b^** | **Microhomology in overlap region ^c^** | **Local homology^d^** | **Nearest genomic feature^e^** |
| --- | --- | --- | --- | --- | --- | --- | --- |
| 6033 | chrII:780898-780904 | YBR289w | SNF5 | 79%* | 5bp | No | 11.3kb to ARS |
|  | chrIV:1116213-1116246 | YDR324c | UTP4 | Incomplete seq | 5bp and 26bp | 38bp (54bp downstream of bpr) | 6kb from ARS |
|  | chrVII:179643-179658 | YGL173c | KEM1 | 84%* | 14bp | 47bp (9bp downstream of bpr) | 6kb from ARS |
|  | chrVIII:433729-433738 | YHR165c | PRP8 | 84% | 8bp | 45bp (129bp upstream of bpr) | 14kb FROM ARS |
|  | chrVIII:451249-451261 | Intergenic | Intergenic | N/A | 9bp | No | 3.4kb from ARS |
|  | chrXIII:843622-843635 | YMR287c | MSU1 | 79%* | 12bp | No | 5.9kb from Ty1 LTR |
|  | chrXVI:97018-97048 | YPL240c | HSP82 | Incomplete seq | 29bp | 32bp (151bp downstream of bpr) and 44bp (238bp downstream of bpr) | 19.4kb from ARS |
|  | chrXVI:482999-483013 | YPL036w | PMA2 | 84%* | 13bp | No | 28.9kb from ARS |
|  | chrXVI:906846-906880 | Intergenic | Intergenic | N/A | 33bp | 32bp (1bp upstream of breakpoint) | 25.6kb from Ty4 |
| 6261 | chrIV:1115814-1115829 | YDR324c | UTP4 | Incomplete seq | 14bp | 37bp (290bp upstream of bpr) | 5.6kb from ARS |
|  | chrV:507240-507255 | YER164w | CHD1 | 82% | 14bp | No | 7.5kb from ARS |
|  | chrVII:179643-179658 | YGL173c | KEM1 | 84%* | 14bp | 47bp (9bp downstream of bpr) | 6kb from ARS |
|  | chrXI:60182-60196 | YKL203c | TOR2 | 82% | 13bp | No | 4.3kb from ARS |
|  | chrXI:285492-285507 | YKL080w | VMA5 | 88% | 14bp | 32bp (66bp downstream of bpr) | 16.5kb from TY1 LTR |
|  | chrXIII:172148-172154 | YML051w | GAL80 | 84% | 5bp | 52bp (172bp upstream of bpr) | 3.3kb from tRNA |
|  | chrXIII:882708-882717 | YMR306w | FKS3 | 83% | 8bp | 32bp (159bp downstream of bpr) | 15kb from ARS |
|  | chrXV:496849-496867 | YOR092w | ECM3 | 83%* | 17bp | No | 9.3kb from tRNA |
|  | chrXV:526415-526427 | YOR109w | INP53 | 82% | 11bp | No | 39kb from tRNA |
|  | chrXV:561420-561425 | YOR127w | RGA1 | 76% | No | No | 5kb from ARS |
|  | chrXVI:97018-97048 | YPL240c | HSP82 | Incomplete seq | 29bp | 32bp (151bp downstream of bpr) and 44bp (238bp downstream of bpr) | 19.4kb from ARS |
| 6257 | chrIV:1148739-1148747 | YDR338c | YDR338c | 83% | 7bp | No | 2.3kb from tRNA and TY1 LTR |
|  | chrVII:179643-179658 | YGL173c | KEM1 | 84%* | 14bp | 47bp (9bp downstream of bpr) | 6kb from ARS |
|  | chrIX:306348-306368 | YIL026c | IRR1 | 80% | 19bp | No | 5.4kb from TY1 LTR and tRNA |
|  | chrX:453940-453961 | YJR009c | TDH2 | 96%* | 20bp | 47bp (adjacent upstream of bpr) and 67bp (adjacent downstream of bpr) | 0.6kb from ARS |
|  | chrXI:354012-354024 | YKL045w | PRI2 | 82% | 11bp | No | 24.5kb from ARS |
|  | chrXIII:602992-602998 | YMR170c | ALD2 | 81%* | 5bp | No | 8.7kb from ARS |
|  | chrXIII:657834-657854 | YMR196w | YMR196w | 84% | 8bp and 5bp | No | 8.4kb from ARS |
|  | chrXVI:97018-97048 | YPL240c | HSP82 | Incomplete seq | 29bp | 32bp (151bp downstream of bpr) and 44bp (238bp downstream of bpr) | 19.4kb from ARS |
|  | chrXVI:862750-862765 | YPR160w | GPH1 | 84% | 14bp | 47bp (adjacent upstream of bpr) and 38bp (adjacent downstream of bpr) | 2.3kb from tRNA |
|  | chrXVI:919949-919955 | YPR191w | QCR2 | 82%* | No | No | 13.2kb from ARS and Ty4 |
|  |  |  | **Mean** | **82.6%** |  |  | **11.41kb** |
|  |  |  | **Stdev** | **2.7%** |  |  | **9.2kb** |

^a^Based on *S. cerevisiae* sequence UCSC SacCer2 June 2008

^b^Nucleotide identity between *S. cerevisiae* and S. eubayanus homologues (FM318, http://hittinger.genetics.wisc.edu/index.html) or *S. uvarum* homologues (SGD, http://www.yeastgenome.org).

^c^Regions of microhomology between 5-30bp observed between *S. cerevisiae* and *S. uvarum* homologues at the breakpoint site using Clustal Omega.

^d^Regions of local homology over 30bp observed between *S. cerevisiae* and *S. uvarum* homologues at or within a few hundred bp of the breakpoint site using Clustal Omega. Bpr: breakpoint region

^e^Nearest repetitive feature to the site of breakpoint formation annotated in the *S. cerevisiae* genome. Feature distance obtained using the UCSC Genome Browser (http://genome.ucsc.edu/).

**S. eubayanus* sequence is incomplete for this gene. We obtained an identity between *S. cerevisiae* and *S. uvarum* (SGD, http://www.yeastgenome.org)
