# Supplementary material for: Sequencing and Characterisation of Rearrangements in Three S. pastorianus Strains Reveals the Presence of Chimeric Genes and Gives Evidence of Breakpoint Reuse
Source: PLoS One. 2014 Mar 18;9(3):e92203. doi: 10.1371/journal.pone.0092203 (PMC3958482; doi:10.1371/journal.pone.0092203)
Supplement: Table S5 — Primer sequences for amplification of breakpoint regions. (DOC) [file pone.0092203.s008.doc]

**Table S5. Primer sequences for amplification of breakpoint regions.**

| **DBVPG strain** | **Breakpoint location** | **Primers^a^** | **Primer sequence (5'-3')** | **Tm (^o^C)** | **Product size (kb)** |
| --- | --- | --- | --- | --- | --- |
| 6033 | chrII:780898-780904 | 6033_SNF5_F (Sc) | GATCCATGAGTGGACAACCTC | 59.8 | 1.23 |
|  |  | 6033_SNF5_R (Se) | GTACCAGTCAACGCAAGCATA | 57.9 |  |
|  | chrIV:1116213-1116246 | IVUTP4_6261_F (Sc) | AGGGCCTCCCTACGACTAAA | 59.4 | 5.46 |
|  |  | IVUTP4_6261_R (Se) | caccgtaagggctcgaatag | 59.4 |  |
|  | chrVII:179643-179658 | VII6261_172a_F (Sc) | AATGTGGCCCATGAGTAGACA | 57.9 | 1.95 |
|  |  | VII6261_172a_R (Sb) | ccgaACAGTCCTCCTGATGT | 59.4 |  |
|  | chrVIII:433729-433738 | 6033_PRP8_F (Sc) | GTTAACGAAGTTGGCACCCTA | 60.3 | 1.37 |
|  |  | 6033_PRP8_R (Se) | ACAACACTTGAGTGAAGCTTGG | 58.4 |  |
|  | chrVIII:451249-451261 | 6033_ENO2_F (Se) | Agaccacttttgtcgaccttttc | 58.9 | 2.10 |
|  |  | 6033_ENO2_R (Sc) | ATGCATATCACTGTGGTCGTG | 57.9 |  |
|  | chrXIII:843622-843635 | 6033_MSU1_F (Sc) | TTGGGTGTACCGAGTATCCTG | 59.8 | 0.92 |
|  |  | 6033_MSU1_R (Se) | GCTGCTCTCCCCTACATCAGT | 61.8 |  |
|  | chrXVI:97018-97048 | Hsp82_6261_F (Sc) | AAGTTGGTTCGTCCAAACTGA | 55.9 | 2.89 |
|  |  | Hsp82_6261_R (Se) | TATCGCGTCTTGGTCTTCTTG | 57.9 |  |
|  | chrXVI:482999-483013 | 6033_PMA2_F (Se) | ATAGTGACGGCGTGCATCTT | 57.3 | 1.67 |
|  |  | 6033_PMA2_R (Sc) | AAATCCGACAGATGCGTTTAAT | 54.7 |  |
|  | chrXVI:906846-906880 | 6033_GDB1_F (Sc) | CAAGCCTGGAGTTCTGCTTGT | 59.8 | 1.8 |
|  |  | 6033_GDB1_R (Se) | AGGATTTCTACTCGCGCTTTG | 57.9 |  |
| 6261 | chrIV:1115814-1115829 | IVUTP4_6261_F (Sc) | AGGGCCTCCCTACGACTAAA | 59.4 | 5.46 |
|  |  | IVUTP4_6261_R (Se) | caccgtaagggctcgaatag | 57.3 |  |
|  | chrV:507240-507255 | VCHD1_6261_F (Sc) | gccaagtacgtggtctgaaaa | 57.9 | 2.03 |
|  |  | VCHD1_6261_R (Se) | TGCGACTCTCCTAAGTCTGGA | 59.8 |  |
|  | chrVII:179643-179658 | VII6261_172a_F (Sc) | AATGTGGCCCATGAGTAGACA | 57.9 | 1.95 |
|  |  | VII6261_172a_R (Se) | ccgaACAGTCCTCCTGATGT | 59.4 |  |
|  | chrXI:60182-60196 | XITOR2_6261_F (Se) | Cacgcttatcggagttttgc | 57.3 | 2.0 |
|  |  | XITOR2_6261_R (Sc) | GCTAATGCAAGGGGTATCTCC | 59.8 |  |
|  | chrXI:285492-285507 | XI_VMA5_6261_F (Sc) | GACCTTAATCGGGGGTAGGG | 61.4 | 0.85 |
|  |  | XI_VMA5_6261_R (Se) | Ccataacgcaacacggattc | 57.3 |  |
|  | chrXIII:172148-172154 | 3rdGAL80_6261_F (Se) | CTACTGGgtacagggcaacac | 61.8 | 2.05 |
|  |  | NEWGAL80_6261_R (Sc) | GTCGTTTGCTCTAGTTCCACTGTA | 61.0 |  |
|  | chrXIII:882708-882717 | FKS3_6261_F (Se) | CTAAGCTGTCGCTTTCAGACG | 59.8 | 2.19 |
|  |  | FKS3_6261_R (Sc) | AATGGGGTACCAAAACGGTAA | 55.9 |  |
|  | chrXV:496849-496867 | XV6261_093C_F (Sc) | TCTTTTAGGCGCTACTCTTGGT | 58.4 | 1.23 |
|  |  | XV6261_093C_R (Sb) | Taaggcaggcggattgttag | 57.3 |  |
|  | chrXV:526415-526427 | XV6261_109W_F (Se) | AGGCACCTCTCTGATCAACC | 59.4 | 1.18 |
|  |  | XV6261_109W_R (Sc) | TATTCCCAGCCATACCCCTA | 57.3 |  |
|  | chrXV:561420-561425 | XV6261_127a_F (Sc) | ACACTTTGGGGACCTGCTG | 58.8 | 1.8 |
|  |  | XV6261_127a_R (Se) | GCCCCTCCGAATCTTTCATA | 57.3 |  |
|  | chrXVI:97018-97048 | Hsp82_6261_F (Sc) | AAGTTGGTTCGTCCAAACTGA | 55.9 | 2.89 |
|  |  | Hsp82_6261_R (Se) | tatcgcgtcttggtCTTCTTG | 57.9 |  |
| 6257 | chrIV:1148739-1148747 | 6257_338_F (Sc) | TAAAAGCATGGCACTTCCAATAC | 63.8 | 1.74 |
|  |  | 6257_338_R (Se) | acagtcgatttcggaagtaacg | 51.2 |  |
|  | chrVII:179643-179658 | VII6261_172a_F (Se) | AATGTGGCCCATGAGTAGACA | 57.9 | 1.95 |
|  |  | VII6261_172a_R (Sc) | ccgaACAGTCCTCCTGATGT | 59.4 |  |
|  | chrIX:306348-306368 | 6257_IRR1_F (Sc) | ACAGACCGTGGAATACGTTCA | 57.3 | 1.19 |
|  |  | 6257_IRR1_R (Se) | aatagtcactggcaacctggtc | 60.3 |  |
|  | chrX:453940-453961 | 6257_Tdh2_F (Sc) | GGTCCTTCAGGCATCAAAT | 54.5 | 3.42 |
|  |  | 6257_Tdh2_R (Se) | cgtaagcctgggtagtcataat | 58.4 |  |
|  | chrXI:354012-354024 | 6257_PRI2_F (Sc) | GCTAACCTCCACCGATCAAA | 57.3 | 2.13 |
|  |  | 6257_PRI2_R (Se) | gctggacgaaaggaagattg | 57.3 |  |
|  | chrXIII:602992-602998 | 6257_ALD2_F (Sc) | TAACCGTGTTACCGGCTGCT | 54.4 | 1.86 |
|  |  | 6257_ALD2_R (Se) | agtgggggtacgcaaaaTCA | 57.4 |  |
|  | chrXIII:657834-657854 | 6257_196W_F (Se) | Aggtcgagtgtccggtaggt | 61.4 | 2.46 |
|  |  | 6257_196W_R (Sc) | GTTCCCTGGCATGAATACGA | 57.3 |  |
|  | chrXVI:97018-97048 | Hsp82_6261_F (Sc) | AAGTTGGTTCGTCCAAACTGA | 55.9 | 2.89 |
|  |  | Hsp82_6261_R (Se) | TATCGCGTCTTGGTCTTCTTG | 57.9 |  |
|  | chrXVI:862750-862765 | 6257_GPH1_F (Sc) | CAACAACGCGCAGAGTCTAT | 57.3 | 2.5 |
|  |  | 6257_GPH1_R (Se) | cTGGGACcccaatcaagata | 57.3 |  |
|  | chrXVI:919949-919955 | 6257_QCR2_F (Se) | aCGCTCCcagtaaagtgtcc | 59.4 | 0.925 |
|  |  | 6257_QCR2_R (Sc) | GATTCATTTTGGACGGCATT | 53.2 |  |

^a^Species specificity of each primer is stated in parentheses. Sc: *S. cerevisiae*. Se: *S. eubayanus*.
